# The Associations of General, Central, Visceral Obesity, and Body Fat Percentage with Cognitive Impairment in the Elderly: Meta-analysis and Mendelian Randomization Study

**DOI:** 10.1016/j.advnut.2025.100550

**Published:** 2025-11-03

**Authors:** Duoduo Lu, Xiang Ji, Yu An, Huiyuan Liu, Gang Zheng, Yashu Liu, Honghao Yang, Yuhong Zhao, Lu Zhao, Yang Xia

**Affiliations:** 1 Department of Clinical Epidemiology, Shengjing Hospital of China Medical University, Shenyang, China; 2 Liaoning Key Laboratory of Precision Medical Research on Major Chronic Disease, Shenyang, China; 3 Medical Research Center, Beijing Institute of Respiratory Medicine and Beijing Chao-Yang Hospital, Capital Medical University, Beijing, China; 4 Department of General Surgery, Shengjing Hospital of China Medical University, Shenyang, China

**Keywords:** cognitive disorders, obesity phenotypes, body mass index, meta-analysis, Mendelian randomization

## Abstract

It is unclear whether various obesity phenotypes are differently associated with cognitive disorders. The aim of this study was to examine the associations of general obesity [measured by body mass index (BMI)], central obesity [measured by waist circumference (WC), waist-to-hip ratio (WHR)], or body fat percentage (BFP), and visceral obesity with cognitive disorders in older adults using meta-analysis and Mendelian randomization (MR) approaches. We identified observational studies published from the inception of 4 (PubMed, Embase, Medline, and Web of Science) databases until November 2024. A random-effects model was employed to construct the pooled odds ratio (OR) and 95% confidence interval (CI) for exploring the associations of general, central, and visceral obesity with cognitive impairment. The mean, median, or range age of participants is ≥60 y. Subsequently, a 2-sample MR analysis was performed using genetic variation data to investigate the potential causal relationships of general, central, and visceral obesity with cognitive impairment. A total of 70 studies comprising 2,810,410 participants were included. In meta-analysis, general obesity (measured by BMI) showed an inverse association with cognitive disorders. Compared with normal BMI, the pooled ORs (95% CIs) were 1.29 (1.21, 1.38) for underweight, 0.87 (0.84, 0.90) for overweight, and 0.88 (0.85, 0.91) for obesity. In contrast, no significant association was observed for central obesity (WC or WHR) or visceral obesity. Subgroup analyses by sex, study design, region, and disease type produced results consistent with the overall findings. In MR analyses, higher BMI, BFP, WC, and WHR were associated with a reduced risk of cognitive impairment, with WHR also inversely related to mild cognitive impairment. No causal association was observed for dementia or Alzheimer’s disease. The research results indicate that there might be a negative correlation between obesity (especially generalized obesity) and cognitive impairment in the elderly.

## Introduction

Aging is frequently accompanied by cognitive disorders, making it the primary risk factor for the development of neurodegenerative disease [1]. Owing to the increasing aging population, cognitive disorders, including dementia, Alzheimer’s disease (AD), and cognitive impairment, have become an increasingly serious issue [2]. The prevalence of dementia is expected to rise from 57 million cases in 2019 to ∼153 million by the year 2050 [3,4]. Moreover, cognitive disorders have shown positive associations with various chronic conditions, such as stroke, cardiovascular disease, peripheral vascular disease, hypertension, and diabetes in older adults [[5], [6], [7], [8]]. As a result, it is imperative to identify potential risk factors for cognitive disorders and devise efficacious preventive measures.

There is increasing evidence that obesity, as a modifiable factor, is associated with the risk of cognitive disorders. According to the WHO definitions, the term “general obesity” refers to an abnormal accumulation of fat and is commonly defined and classified using the BMI, which is a readily measurable indicator [9]. However, the measurement of BMI alone is not reliable, because high BMI alone may be a sign of fat limbs or high muscle content and is not representative, so waist circumferences (WCs) and waist-to-hip ratio (WHR) have also been studied. Additionally, the excessive accumulation of fat in the abdominal region, referred to as central obesity or abdominal adiposity, which is commonly assessed using WC and WHR that serve as key anthropometric indicators [10]. Additionally, visceral obesity is a high-fat accumulation between the abdomen and internal organs [11], which is usually measured by subcutaneous fat thickness and visceral fat thickness. Various obesity phenotypes are associated with distinct metabolic profiles, which may exert differential impacts on cognitive disorders [12].

Beyond these indices, body fat percentage (BFP) provides a direct estimate of fat mass relative to total body weight and reflects overall adiposity more precisely than BMI. This is especially relevant in older adults, who frequently experience sarcopenia and fat redistribution. Higher BFP has been linked to poorer cognitive performance, independent of BMI and cardiovascular disease risk factors [13]. Longitudinal evidence further suggests that higher fat mass increases dementia risk, whereas lower lean mass shows the opposite association [14]. However, findings remain inconsistent, partly because of differences in sex, fat distribution, and measurement methods, such as dual-energy X-ray absorptiometry and bioelectrical impedance analysis [15,16]. Taken together, incorporating BFP alongside BMI, WC, and visceral fat measures may provide a more comprehensive understanding of adiposity-cognition associations in the elderly.

The discovery of the “obesity paradox” has refreshed our previous conclusions about obesity. Obesity may not necessarily shorten the survival time of patients, and in some diseases, individuals with overweight have a slightly lower risk of death than those of normal weight. In some cases, it may even have “benefits” [17]. A comprehensive meta-analysis of 21 longitudinal cohort studies involving 5,060,687 individuals demonstrated that central obesity measured by WC was significantly associated with an increased risk of cognitive impairment [18]. Another meta-analysis, comprising 29 cohort studies and 4,978,621 individuals, revealed that a higher level of BMI appeared to be negatively associated with dementia [19]. Previous meta-analyses have mostly focused on the association between general obesity and cognition, primarily in the general population. However, they have not explored the complex association between different types of obesity and cognition in the elderly under the context of the “obesity paradox.”

Because of the inherent limitations of observational studies in causal inference, Mendelian randomization (MR) research has become an important supplementary method for causal inference [20]. At present, MR research on obesity and cognitive disorders is mostly focused on the impact of obesity on mild cognitive impairment (MCI), and there is no systematic study on the relationship between different types of obesity and different types of cognitive disorders [[21], [22], [23]].

Therefore, our main objective is to conduct a comprehensive meta-analysis to investigate the potential associations of general, central, visceral obesity, and BFP with cognitive disorders. Furthermore, we performed subgroup analyses considering sex, study design, study area, and cognitive outcomes. Finally, we conducted a 2-sample MR to evaluate the causal relationships of BMI, BFP, WC, WHR, and visceral adipose tissue (VAT) [24] with AD, cognitive impairment, dementia, and MCI, thereby providing evidence for preventive strategies against cognitive disorders.

## Methods

### Meta-analysis

#### Search strategy and selection criteria

The study selection procedure adhered to the PRISMA guidelines [25]. The protocol for this meta-analysis has been registered in PROSPERO (CRD42023449444).

We conducted a comprehensive search across 4 databases, including PubMed, Embase, Medline, and Web of Science, for relevant studies published up to 5 November, 2024. To avoid omission, we conducted additional searches in relevant meta-analyses to include the selected literature. Our search approach involved the integration of 4 distinct search themes: general obesity (measured by BMI), central obesity (measured by WC or WHR), visceral obesity (measured by subcutaneous fat thickness or visceral fat thickness), or BFP (exposure); cognitive disorders, such as cognitive impairment, dementia, AD, or MCI (outcome); older adults (study population); and observational study (study design). The detailed exploration plan can be found in Supplemental Table 1.

#### Inclusion and exclusion criteria

The inclusion criteria were as follows: *1*) The study that investigated the associations of cognitive disorders with general obesity, visceral obesity, central obesity, or BFP; *2*) Observational studies, including cohort, case-control, or cross-sectional studies; *3*) The mean, median, or range age of participants is ≥60 y; *4*) Reported the relative risks, hazard ratios, or odds ratios (ORs) with corresponding 95% confidence intervals (CIs) or adequate information for calculating these measures; and *5*) Original article published in English. The exclusion criteria were as follows: *1*) Considering death because of cognitive disorders as the outcome; *2*) Using continuous BMI, WC, or WHR as measures; *3*) Being letters to the editors, case reports, studies on animals, or reviews; or *4*) Presenting nonsummarizable risk estimates (e.g., reporting risk estimates lacking 95% CIs).

#### Data extraction and quality assessment

Two reviewers independently screened and identified eligible studies. From each included article, we extracted the following information: publication year, first author, study design, country or region, participants’ mean age, median age, or age range, duration of follow-up, sample size, exposure variables (general obesity, central obesity, visceral obesity, or BFP), outcome types (AD, MCI, dementia, or cognitive impairment), methods of outcome assessment, and covariates adjusted in the analysis.

We employed the Newcastle-Ottawa (NOS) and the Agency for Healthcare Research and Quality to evaluate the quality. The NOS was used to assess the quality of case-control and cohort studies, with a range of 0–9 scores [26]. Additionally, the Agency for Healthcare Research and Quality tool was employed to assess the quality of the cross-sectional study. There were 11 questions in total, ranging from 0 to 11 [27].

#### Statistical analysis

To evaluate the associations of general obesity, central obesity, visceral obesity, and BFP with cognitive disorders, we used pooled ORs and their 95% CIs as measurements of effect size in a random-effects model [28]. For studies reporting sex-specific but no overall estimates, subgroup log effect sizes were first pooled within study using inverse-variance fixed-effect weighting to obtain a single study-level estimate and SE; only this study-level estimate was then entered once into the between-study random-effects meta-analysis (Supplemental Table 2). The *I*^2^ statistic was used to estimate heterogeneity among included studies. The *I*^2^ metric classifies heterogeneity into 3 categories: low, moderate, and high, with corresponding cutoff percentages of 25%, 50%, and 75%, respectively [29]. To identify the sources of heterogeneity among studies, we conducted subgroup analyses based on gender (male or female), study design (cohort, cross-sectional, or case-control studies), study regions (Europe, America, Asia, or others), and type of disease (cognitive impairment, dementia, MCI, or AD). Begg’s test, funnel plot, and Egger’s test were employed to evaluate the presence of publication bias. The statistical analysis was performed using Stata (version 17.0) software. Two-sided *P* < 0.05 was considered statistically significant.

### MR analysis

#### MR design and assumptions

We performed a 2-sample MR to investigate the causal effect of obesity-related traits on cognitive outcomes. MR relies on the following 3 core assumptions: *1*) relevance: the instrumental variants (IVs) are strongly associated with the exposure; *2*) independence: the IVs are independent of any confounders between the exposure and outcome; *3*) the IVs are associated with the outcome only through the exposure. Regarding how we handle these hypotheses in our research, first of all, the relevance: we selected single nucleotide polymorphisms (SNPs) at genome-wide significance (*P* < 5 × 10^-8^) and excluded weak instruments based on *F*-statistics. Second, independence: to minimize associations between instruments and potential confounders, we restricted analyses to European ancestry, performed linkage disequilibrium (LD) clumping, and screened SNPs against genome-wide association study (GWAS) resources to exclude those linked to known confounders. The independence aspect is reflected in Supplemental Table 3. Finally, exclusion restriction: we evaluated this assumption using sensitivity analyses, which included MR-Egger regression, weighted median, leave-one-out, MR-PRESSO. The MR-Egger intercept and Cochran’s *Q* statistic were used to detect directional pleiotropy and heterogeneity (Supplemental Table 4).

#### Data sources

Summary-level GWAS data for both exposures and outcomes were obtained from publicly available resources. For exposures, we used BMI, WC, WHR, VAT, and BFP. For outcomes, we used cognitive performance, dementia [30], and AD [31]. Regarding the outcome, as there are currently no large-scale GWAS specifically targeting “cognitive impairment” or “MCI” diagnoses, we used 2 genetic proxy phenotypes that are most closely related to cognitive impairment to define the outcome. First, we used the aggregated data on cognitive functions. This dataset is based on a population sample and quantifies overall cognitive functions through a series of forward-looking memory, reaction time, and number memory tests [32]. Second, we included the aggregated data on cognitive performance. This study integrated the neuropsychological test results from multiple cohorts, forming a large-scale, comprehensive indicator of cognitive ability [33]. In the fields of neurology and geriatrics, a decline in objective cognitive test performance is the cornerstone for diagnosing cognitive impairment [34,35]. Therefore, using these 2 phenotypic genetic tools to represent the genetic susceptibility to “cognitive impairment” is reasonable. By combining these 2 datasets, we can more comprehensively and reliably capture the genetic background related to the risk of cognitive impairment. Detailed information on all datasets is provided in Supplemental Table 5.

#### Instrumental variables selection

The genome-wide significance level for instrument variables (IVs) was set at *P* < 5 × 10^−8^. Moreover, the identified SNPs were clumped for LD using a stringent clumping threshold of *R*^2^ < 0.001 within a 10,000 kb window, with LD estimated using the European samples from the 1000 Genome Project as the reference [36]. We applied a PhenoScanner search to identify all known phenotypes associated with genetic IVs (*P* < 5 × 10^−8^). If the genetic IV is associated with any other known phenotype, it would be excluded from subsequent MR analysis. The *F*-statistic is calculated as *F* = (*N*–*K*–1)/*K* × *R*^2^/(1–*R*^2^), where *K* = number of SNPs used as instruments, *N* = sample size in the discovery GWAS, and *R*^*2*^ reflects the degree to which the instrumental variable explains the exposure.

#### Statistical analysis

In this study, we selected the inverse-variance weighted (IVW) method as the primary MR approach because it is the most widely used estimator and provides the most efficient causal estimates when all instrumental variables are valid [37]. To account for potential heterogeneity across SNP-specific estimates, we applied the multiplicative random-effects IVW model whenever heterogeneity was detected. This random-effects specification provides more conservative estimates while maintaining statistical power.

To mitigate this bias, we conducted sensitivity analyses using the weighted median [38], MR-Egger [39], and MR-PRESSO [40], each of which makes distinct assumptions regarding the presence of outliers and pleiotropy. Cochran’s Q value was used to evaluate the heterogeneity among estimates from SNPs, whereas horizontal pleiotropy was assessed using the MR-Egger intercept. All estimates were reported as ORs or *β* coefficients with corresponding 95% CIs.

Statistical analyses were performed in *R* (version 4.4.3) using the packages “TwoSampleMR” and “MRPRESSO.”

## Results

### Meta-analyses

#### Study characteristics and quality assessment

**FIGURE 1:**
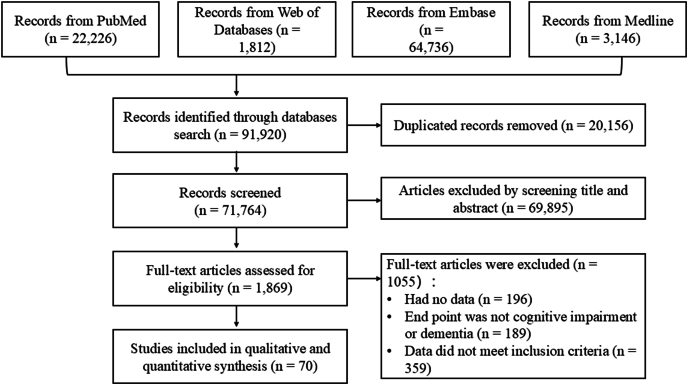
The flowchart of the study inclusion process.

The flow chart of the included studies is depicted in Figure 1. Initially, we identified a total of 91,920 potentially relevant records, of which 20,156 duplicates were excluded from the analysis. Subsequently, the titles and abstracts of the remaining 71,764 studies underwent screening. Ultimately, a total of 70 studies were deemed eligible for inclusion.

Supplemental Table 6 summarizes the characteristics of the study. A total of 2,810,410 individuals were involved in the research, with a minimum baseline mean, median, or minimum age range is ≥60 y old. Regarding gender, 1 study exclusively involved male participants [41]. Of these studies, 52 provided data on BMI [14,[41], [42], [43], [44], [45], [46], [47], [48], [49], [50], [51], [52], [53], [54], [55], [56], [57], [58], [59], [60], [61], [62], [63], [64], [65], [66], [67], [68], [69], [70], [71], [72], [73], [74], [75], [76], [77], [78], [79], [80], [81], [82], [83], [84], [85], [86], [87], [88], [89], [90], [91]], and 3 provided data on BFP [43,52,65]. As for other metrics, 12 studies reported data solely on WC [[92], [93], [94], [95], [96], [97], [98], [99], [100], [101], [102], [103]], 1 study focused only on WHR [104], 5 studies presented data on both WC and WHR [41,50,63,65,66], and 5 studies provided data regarding visceral obesity [[105], [106], [107], [108], [109]]. Of these 70 studies, 50 were cohort studies, 18 were cross-sectional studies, and the remaining 2 were case-control studies. The majority of the studies originated from Asia (*n* = 38), 16 from Europe, 13 from America, and 2 each from Oceania or mixed regions.

Supplemental Table 7 presents the evaluation of the included studies’ quality. The cohort studies received ratings ranging from 6 to 9 stars, whereas the cross-sectional studies were scored between 8 and 10 points. All included studies demonstrated medium to high levels of quality.

#### General obesity and cognitive disorders

**FIGURE 2:**
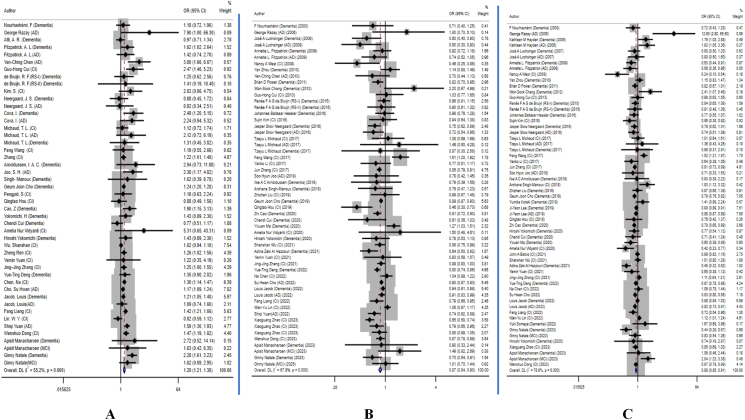
Forest plots for the association between general obesity and cognitive disorders: (A) pooled association between underweight and cognitive disorders; (B) pooled association between overweight and cognitive disorders; (C) pooled association between obesity and cognitive disorders. AD, Alzheimer’s disease; CI, confidence interval; DL, DerSimonian and Laird ; MCI, mild cognitive impairment; OR, odds ratio; RS-I, Rotterdam Study I, original cohort; RS-II, Rotterdam Study II, extended cohort.

As measured by BMI, general obesity is classified into 3 categories: underweight, overweight, and obesity. The association between underweight and cognitive disorders was investigated across 39 studies, encompassing 2,742,049 individuals. The pooled results revealed a positive association between being underweight and cognitive disorders (OR: 1.29; 95% CI: 1.21, 1.38) (Figure 2A).

All the studies were divided into 3 subgroups according to the types of study design. The pooled ORs (95% CIs) in cohort, cross-sectional, and case-control studies were 1.31 (1.22, 1.41), 1.15 (0.98, 1.36), and 2.53 (0.87, 7.36), respectively. Analyses stratified by gender showed that underweight was positively associated with cognitive disorders in both males and females. The pooled ORs (95% CIs) in males and females were 1.23 (1.12, 1.36) and 1.28 (1.17, 1.40), respectively. We categorized the studies into 4 subgroups (Asia, Europe, America, and Mixed) according to the regions where the study was conducted. The combined pooled ORs (95% CIs) for Asia, Europe, America, and mixed populations were 1.26 (1.17, 1.36); 1.20 (1.08, 1.34); 1.45 (1.15, 1.84); and 7.90 (0.97, 64.33), respectively. In the subgroup analysis based on the type of cognitive disorders, underweight was found to be significantly associated with higher prevalence of AD (OR: 1.54; 95% CI: 1.21, 1.95), cognitive impairment (OR: 1.24; 95% CI: 1.10, 1.39), and dementia (OR: 1.31; 95% CI: 1.17, 1.47).

The association between overweight and cognitive disorders was investigated across 44 studies with 2,769,447 individuals. The pooled result from the random-effects model revealed that being overweight was inversely associated with cognitive disorders (OR: 0.87; 95% CI: 0.84, 0.90) (Figure 2B).

In a subgroup analysis focusing on study design, all studies were divided into 3 subgroups based on the type of study design. The pooled ORs (95% CIs) in cohort, cross-sectional, and case-control studies were 0.86 (0.83, 0.89); 0.90 (0.78, 1.03); and 1.19 (0.66, 2.14), respectively. Subgroup analysis according to gender indicated that overweight was negatively associated with cognitive disorders in both males (OR: 0.89; 95% CI: 0.84, 0.94) and females (OR: 0.90; 95% CI: 0.89, 0.92). The combined pooled ORs (95% CIs) for Asia, Europe, America, Oceania, and mixed populations were 0.89 (0.85, 0.93); 0.89 (0.82, 0.97); 0.74 (0.66, 0.84); 0.82 (0.70, 0.96); and 1.80 (0.67, 4.86), respectively. In a subgroup analysis stratified by the type of cognitive disorder, the results showed a negative association between being overweight and dementia (OR: 0.87; 95% CI: 0.82, 0.92), AD (OR: 0.84; 95% CI: 0.77, 0.92), and cognitive impairment (OR: 0.88; 95% CI: 0.80, 0.97).

A total of 47 studies involving 2,688,425 individuals were conducted to investigate the association between obesity and cognitive disorders. The result of the random-effects model revealed that obesity was negatively associated with cognitive disorders, with an OR of 0.88(95% CI: 0.85, 0.91) (Figure 2C).

According to the research design, the study was divided into 3 subgroups, and the pooled ORs (95% CIs) in cohort, cross-sectional, and case-control studies were 0.85 (0.82, 0.88); 1.00 (0.86, 1.17); and 3.82 (0.49, 22.86), respectively. A negative correlation was found between obesity and cognitive impairment in cohort studies. Both males (OR: 0.85; 95% CI: 0.79, 0.90) and females (OR: 0.83; 95% CI: 0.79, 0.88) showed a negative association between obesity and cognitive disorders. Additionally, subgroup analysis based on the study area showed a significant negative correlation between obesity and cognitive disorders in the Asian population (OR: 0.90; 95% CI: 0.86, 0.93), European population (OR: 0.85; 95% CI: 0.78, 0.93), American population (OR: 0.78; 95% CI: 0.62, 0.99), Oceanian population (OR: 0.82; 95% CI: 0.67, 1.01), and mixed population (OR: 12.60; 95% CI: 2.80, 56.60). In subgroup analysis stratified by type of cognitive disorders showed a significant negative correlation between obesity and dementia (OR: 0.84; 95% CI: 0.79, 0.90), and AD (OR: 0.85; 95% CI: 0.80, 0.92). The corresponding subgroup results are shown in Table 1.

**TABLE 1:** Subgroup analysis results between general obesity and cognitive impairment

Factor	Underweight				Overweight				Obesity			
Subgroup analysis	Studies	OR (95% CI)	*P* value	*I*^*2*^ (%)	Studies	OR (95% CI)	*P* value	*I*^*2*^ (%)	Studies	OR (95% CI)	*P* value	*I*^*2*^ (%)
Gender												
Male	15	1.23 (1.12, 1.36)	0.094	33.40	18	0.89 (0.84, 0.94)	<0.001	69.90	21	0.85 (0.79, 0.90)	<0.001	72.50
Female	16	1.28 (1.17, 1.40)	0.003	55.30	18	0.90 (0.89, 0.92)	0.460	—	19	0.83 (0.79, 0.84)	<0.001	76.70
Study design												
Cohort study	27	1.31 (1.22, 1.41)	0.001	57.40	29	0.86 (0.83, 0.89)	<0.001	54.00	34	0.85 (0.82, 0.86)	<0.001	76.90
Cross-sectional study	8	1.15 (0.98, 1.36)	0.177	31.50	12	0.90 (0.78, 1.03)	<0.001	79.90	13	1.00 (0.86, 1.17)	0.001	63.80
Case-control study	3	2.53 (0.87, 7.36)	0.008	79.40	3	1.19 (0.66, 2.14)	0.014	76.70	2	3.82 (0.49, 29.88)	0.006	86.50
Continent												
Asia	23	1.26 (1.17, 1.36)	<0.001	62.30	25	0.89 (0.85, 0.93)	<0.001	71.80	28	0.90 (0.86, 0.93)	<0.001	82.80
Europe	9	1.20 (1.08, 1.34)	0.526	—	9	0.89 (0.82, 0.97)	<0.001	68.50	9	0.85 (0.78, 0.93)	0.022	50.60
America	5	1.45 (1.15, 1.84)	0.020	55.80	8	0.74 (0.68, 0.84)	0.133	31.30	9	0.78 (0.62, 0.99)	<0.001	75.00
Oceania	—	—	—	—	1^1^	0.82 (0.70, 0.96)	<0.001	—	1^1^	0.82 (0.67, 1.01)	<0.001	—
Mixed	1^1^	7.90 (0.97, 67.33)	<0.001	—	1^1^	1.80 (0.67, 4.86)	<0.001	—	1^1^	12.60 (2.80, 56.60)	<0.001	—
Disease type												
AD	10	1.54 (1.21, 1.95)	0.002	55.30	10	0.84 (0.77, 0.92)	0.069	43.40	10	0.85 (0.80, 0.92)	<0.001	75.90
Dementia	19	1.31 (1.17, 1.47)	0.039	38.90	22	0.87 (0.82, 0.92)	<0.001	65.30	27	0.84 (0.79, 0.90)	<0.001	79.50
Cognitive impairment	15	1.24 (1.10, 1.39)	<0.001	55.10	18	0.88 (0.80, 0.97)	<0.001	77.60	19	0.91 (0.81, 1.02)	<0.001	76.80
MCI	2	1.62 (0.94, 2.81)	0.994	—	2	1.14 (0.80, 1.62)	0.273	16.90	2	1.29 (0.53, 3.11)	0.008	86.00

Abbreviations: AD, Alzheimer’s disease; CI, confidence interval; MCI, mild cognitive impairment; OR, odds ratio

1 Indicates that it contains result from 1 study.

#### Central obesity and cognitive disorders

**FIGURE 3:**
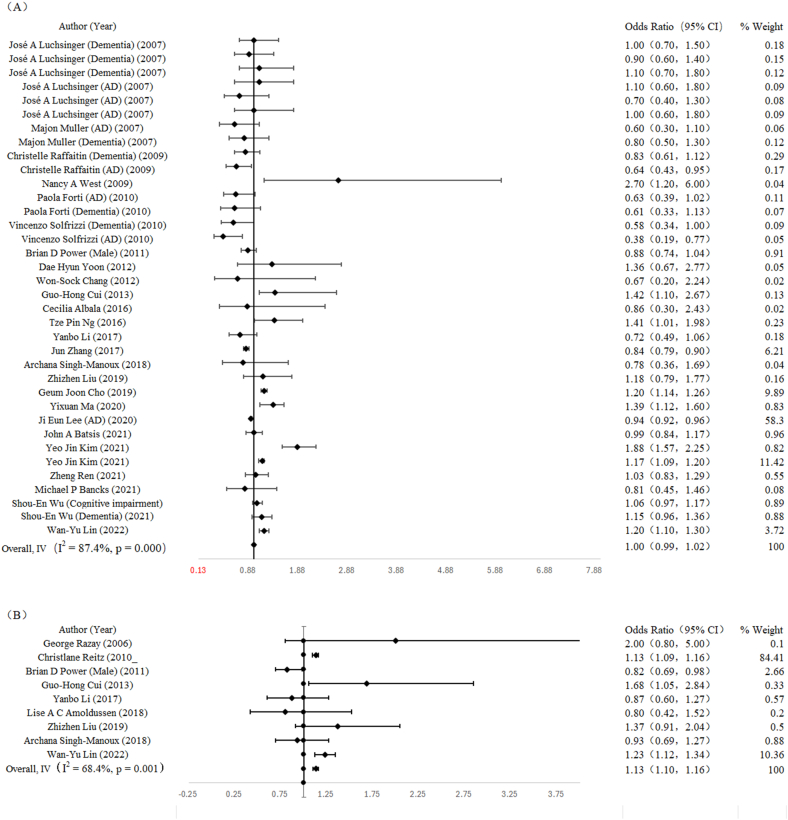
(A) Forest plots for the association between waist circumference and cognitive disorders. (B) Forest plots for the association between waist-to-hip ratio and cognitive disorders. AD, Alzheimer’s disease.

Data from 25 studies with 1,072,293 participants were analyzed to evaluate this association. The combined result, utilizing the random-effects model, revealed no significant association between WC and cognitive disorders (OR: 1.00; 95% CI: 0.99, 1.02) (Figure 3A).

According to the research design, the pooled ORs (95% CIs) in cohort, cross-sectional, and case-control studies were 0.85 (0.82, 0.88); 1.00 (0.86, 1.17); and 3.82 (0.49, 22.86), respectively. A negative correlation was found between obesity and cognitive impairment in cohort studies. Both males (OR: 0.85; 95% CI: 0.79, 0.90) and females (OR: 0.83; 95% CI: 0.79, 0.88) showed a negative association between obesity and cognitive disorders. Additionally, subgroup analysis based on the study area showed a significant negative correlation between obesity and cognitive disorders in the Asian population (OR: 0.90; 95% CI: 0.86, 0.93), European population (OR: 0.85; 95% CI: 0.78, 0.93), American population (OR: 0.78; 95% CI: 0.62, 0.99), Oceanian population (OR: 0.82; 95% CI: 0.67, 1.01), and mixed population (OR: 12.60; 95% CI: 2.80, 56.60). In subgroup analysis stratified by type of cognitive disorders showed a significant negative correlation between obesity and dementia (OR: 0.84; 95% CI: 0.79, 0.90), and AD (OR: 0.85; 95% CI: 0.80, 0.92). The corresponding subgroup results are shown in Table 2.

**TABLE 2:** Subgroup analysis results between central obesity and cognitive impairment

Factor	WC				WHR			
Subgroup analysis	Studies	OR (95% CI)	*P* value	*I*^*2*^ (%)	Studies	OR (95% CI)	*P* value	*I*^*2*^ (%)
Gender								
Male	4	1.07 (1.01, 1.14)	<0.001	90.90	3	1.04 (0.93, 1.17)	0.002	84.50
Female	3	1.05 (1.01, 1.11)	<0.001	95.60	2	1.20 (1.08, 1.34)	0.070	69.50
Study design								
Cohort study	17	1.04 (1.03, 1.05)	<0.001	93.70	5	1.11 (1.08, 1.14)	<0.001	82.80
Cross-sectional study	3	1.18 (1.09, 1.28)	0.031	66.10	3	1.21 (1.11, 1.32)	0.183	41.40
Case-control study	—	—	—	—	1^1^	2.00 (0.80, 5.00)	<0.001	—
Continent								
Asia	15	1.01 (1.00, 1.02)	<0.001	91.80	4	1.23 (1.14, 1.34)	0.099	48.80
Europe	3	0.87 (0.76, 0.99)	0.052	61.1	1^1^	1.13 (1.10, 1.17)	<0.001	—
America	1^1^	0.88 (0.74, 1.04)	<0.001	—	1^1^	0.82 (0.69, 0.98)	<0.001	—
Oceania	1^1^	0.86 (0.30, 2.45)	<0.001	84.4	1^1^	0.93 (0.69, 1.27)	<0.001	—
Mixed	6	1.14 (1.12, 1.16)	<0.001	—	1^1^	2.00 (0.80, 5.00)	<0.001	—
Disease type								
Dementia	5	0.99 (0.97, 1.00)	<0.001	81.00	2	1.13 (1.10, 1.17)	0.222	32.90
cognitive impairment	10	1.07 (1.06, 1.08)	<0.001	94.70	2	0.85 (0.73, 0.99)	0.476	—
MCI	9	0.99 (0.95, 1.04)	<0.001	85.20	4	1.23 (1.14, 1.34)	0.099	48.8

Abbreviations: CI, confidence interval; MCI, mild cognitive impairment; OR, odds ratio; WC, waist circumferences; WHR, waist-to-hip ratio

1 Indicates that it contains result from 1 study.

The association between WHR and cognitive disorders was examined in 9 studies, involving a total of 57,103 participants. The analysis revealed no significant association between WHR and cognitive disorders, as indicated by a pooled OR of 1.13 (95% CI: 1.10, 1.16) (Figure 3B).

Analyses stratified by study region or study design revealed a significant positive association between WHR and cognitive disorders in cross-sectional studies (OR: 1.22; 95% CI: 1.01, 1.48), as well as among individuals in Asian populations (OR: 1.22; 95% CI: 1.01, 1.48). For different types of cognitive disorders, WHR was positively associated with cognitive impairment (OR: 1.22; 95% CI: 1.01, 1.48), negatively associated with dementia (OR: 0.84; 95% CI: 0.73, 0.98). The subgroup analysis results between central obesity and cognitive impairment are shown in Table 2.

#### Visceral obesity and cognitive disorders

**FIGURE 4:**
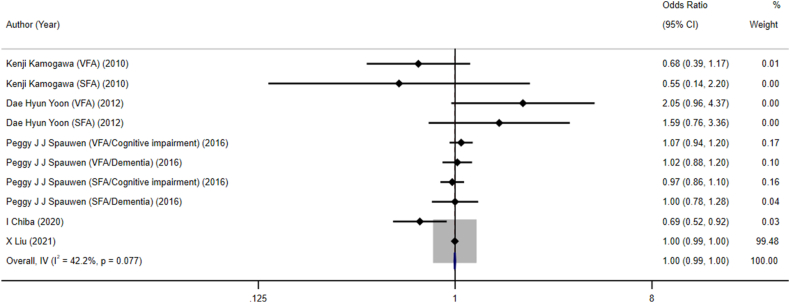
Forest plots for the association between visceral obesity and cognitive disorders. VFA, volatile fatty acids.

A combined sample size of 15,959 individuals was included in 6 studies that investigated the association of visceral obesity with cognitive disorders. The pooled results revealed no significant association between visceral obesity and cognitive disorders, with an OR (95% CI) of 1.00 (95% CI: 0.99, 1.00) (Figure 4). Additional subgroup analyses were performed considering factors such as gender, study design, study regions, and disease type; however, no significant association between visceral obesity and cognitive disorders was found (Table 3).

**TABLE 3:** Subgroup analysis results between visceral obesity and cognitive impairment

Factor	Visceral obesity			
Subgroup analysis	Studies	OR (95% CI)	*P* value	*I*^*2*^ (%)
Gender				
Male	5	0.98 (0.86, 1.11)	<0.001	64.90
Female	4	1.00 (0.96, 1.04)	<0.001	5.10
Study design				
Cohort study	1^1^	1.02 (0.95, 1.09)	<0.001	—
Cross-sectional study	1^1^	0.94 (0.71, 1.25)	<0.001	64.60
Continent				
Asia	4	0.94 (0.71, 1.25)	<0.001	64.60
Europe	1^1^	1.02 (0.95, 1.09)	<0.001	—
Disease type				
cognitive impairment	5	0.98 (0.88, 1.08)	<0.001	54.70

Abbreviations: CI, confidence interval; OR, odds ratio

1 Indicates that it contains result from 1 study.

#### BFP and cognitive disorders

**FIGURE 5:**
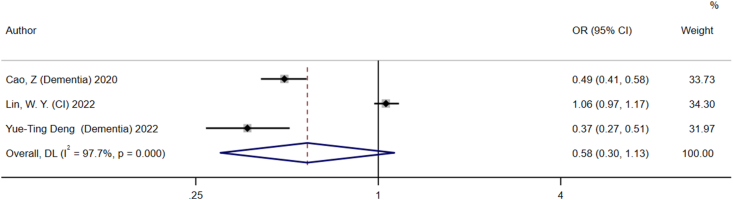
Forest plot illustrating the association between body fat percentage and cognitive impairment. CI, cognitive impairment; OR, odds ratio.

In these 3 studies, a total of 820,013 individuals were included to investigate the association between BFP and cognitive disorders. The pooled results revealed no significant association between BFP and cognitive disorders, with an OR (95% CI) of 0.58 (0.30, 1.13) (Figure 5). Additional subgroup analyses were performed considering factors such as gender, study design, study regions, and disease type; however, no significant association between BFP and cognitive disorders was found (Table 4).

**TABLE 4:** The results of the group analysis on the relationship between body fat percentage and cognitive impairment

Factor	Body fat percentage			
Subgroup analysis	Studies	OR (95% CI)	*P* value	*I*^*2*^ (%)
Study design				
Cohort study	2	0.44 (0.34, 0.58)	0.129	56.7
Cross-sectional study	1^1^	1.06 (0.97, 1.16)	<0.001	—
Continent				
Asia	2	0.72 (0.34, 1.54)	<0.001	98.30
Europe	1^1^	0.37 (0.27, 0.51)	<0.001	—
Disease type				
Dementia	2	0.44 (0.34, 0.58)	0.129	56.70%
Cognitive impairment	1^1^	1.06 (0.97, 1.16)	<0.001	—

Abbreviations: CI, confidence interval; OR, odds ratio

1 Indicates that it contains result from 1 study.

Sensitivity analysis, which involved the successive exclusion of articles, confirmed the stability of these results (Supplemental Figure 1).

#### Publication bias

Supplemental Figure 2 and Supplemental Figure 3 show that no publication bias was found in this meta-analysis. The Begge’s regression test did not reveal any significant bias in the associations between general obesity and cognitive disorders, with *P* values of 0.12, 0.54, and 0.36 for underweight, overweight, and obesity, respectively. Similarly, the association between central obesity and cognitive disorders, the *P* values for WC and WHR were 0.22 and 0.60, respectively. The association between visceral obesity and cognitive disorders was nonsignificant according to Begg’s test (*P* = 1.00). Furthermore, the association between BFP and cognitive disorders was nonsignificant according to Begg’s test (*P* = 1.00).

### MR analysis

#### Characteristics of SNPs

In our study, a total of 508 SNPs were associated with BMI, 257 SNPs were associated with BFP, 337 SNPs were associated with WC, 241 SNPs were associated with WHR, and 5 SNPs were associated with VAT. Detailed information on GWAS and SNPs as genetic tools for general obesity, central obesity, BFP, and visceral obesity can be found in Supplemental Table 5 and Supplemental Table 3.

#### MR analysis of 3 types of obesity and cognitive impairment

**FIGURE 6:**
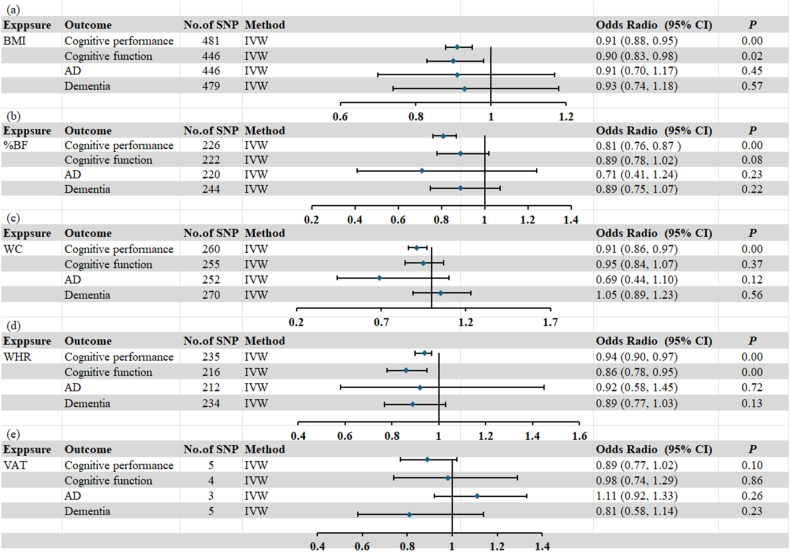
The forest plot of Mendelian randomization results indicates a causal relationship between BMI, WC, WHR, and %BF and cognitive impairment. %BF, body fat percentage; AD, Alzheimer’s disease; CI, confidence intervals; IVW, inverse-variance weighted; OR, odds ratio; SNP, single nucleotide polymorphisms; VAT, visceral adipose tissue; WC, waist circumferences; WHR, waist-to-hip ratio.

The MR analysis of the 2 groups of samples revealed that there was a causal relationship between BMI and cognitive impairment and MCI (IVW method *P* < 0.05), and it was negatively correlated. BFP had a causal relationship with cognitive impairment (IVW method, *P* < 0.05), and it was negatively correlated as well. In central obesity, WC was negatively correlated with cognitive impairment, and WHR was negatively correlated with cognitive impairment and MCI (*P* < 0.05 for all IVW methods). We did not find a causal relationship between VAT and cognitive impairment (*P* < 0.05 for all IVW methods). For detailed information on the MR results, refer to Figure 6.

#### Sensitivity analysis

The scatter plot illustrates the causal effects of SNPs on the relationship between exposure and outcome, as detailed in Supplemental Figure 4. Sensitivity analysis using the IVW method showed that all exposure and outcome MR analyses were presented as *Q-*statistics and *P* values in Supplemental Table 4. The MR-Egger intercept test showed that all analyses had *P* values exceeding 0.05, indicating a lack of horizontal pleiotropy. The impact of a single SNP on the estimation of overall causal effects is shown in the funnel plot (Supplemental Figure 5). The overall MR estimate results were not significantly influenced by any individual SNP, as determined by the leave-one-out approach (Supplemental Figure 6). The detailed results of the sensitivity analysis on MR can be found in Supplemental Figure 7.

## Discussion

In this study, integrating evidence from meta-analysis and MR, we evaluated how distinct obesity phenotypes relate to late-life cognitive disorders. The meta-analysis suggested that overweight and obesity were associated with a lower prevalence of cognitive disorders overall, whereas underweight and WHR were linked to a higher risk. By contrast, central obesity, defined by WC, visceral obesity, and BFP, showed no significant associations. Complementing these patterns, MR analyses indicated inverse associations of genetically predicted general and central adiposity with cognitive impairment, while showing no clear evidence for dementia or AD; no robust signals were observed for visceral adiposity. Together, these results suggest that although both observational and genetic findings support a potential protective relationship for late-life cognitive impairment, the associations with dementia remain uncertain.

With respect to general obesity, our findings are consistent with previous epidemiologic evidence, supporting a potential inverse association between overweight or obesity and the risk of cognitive disorders overall. Several observational studies have suggested that being overweight or obese in late life may reduce the likelihood of developing cognitive disorders [110], whereas being underweight has been associated with elevated risk [42,67,111]. A recent meta-analysis of longitudinal cohort studies published in 2022 similarly reported a protective association between higher BMI and cognitive impairment [23], although that study did not conduct stratified analyses by study characteristics. In contrast, our analysis incorporated subgroup evaluations by sex, study design, and region, providing a more nuanced understanding of how the relationship between general obesity and cognitive disorders may vary across different populations.

Regarding BFP, our meta-analysis did not find a relationship with cognitive impairment, but in the MR analysis, there was a negative correlation between cognitive impairment and MCI. We consider that this might be caused by the following reasons. First, there are potential confounding factors in the meta-analysis that affect both BFP and cognitive function. Studies have shown that long-term lack of exercise not only increases BFP but also increases the risk of cognitive impairment; an unhealthy diet structure, such as high-sugar and high-fat diets, can cause an increase in BFP, and at the same time affect the metabolism and function of the brain, thereby affecting cognition [14]. However, the 2-sample MR can effectively reduce the influence of confounding factors: the 2-sample MR uses genetic variations as instrumental variables, which are randomly assigned at the time of an individual's birth and are not affected by confounding factors such as lifestyle and environmental factors. For example, in the MR study investigating the relationship between obesity and cognitive function, by using specific genetic variations as the tool variable, the potential impact of obesity on cognitive function was discovered, which was difficult to clarify in traditional observational studies because of the interference of confounding factor [21]. This enables the 2-sample MR to more effectively reveal the potential causal relationship between BFP and cognitive impairment. In summary, the relationship between BFP and cognitive impairment requires further research to clarify their association.

The evidence for central obesity and cognitive outcomes remains inconclusive. Although some studies have reported that higher WC was associated with an increased risk of cognitive disorders [18], our meta-analysis restricted to individuals aged ≥60 y did not identify a significant association, whereas MR analyses suggested a potential protective effect. Differences in age composition may partly explain these discrepancies, as the influence of adiposity on cognitive health appears to vary across the life course [42]. In our subgroup analyses, higher WHR was positively associated with cognitive impairment, consistent with previous reports [50]; however, WHR showed an inverse association with dementia. This apparent contradiction may reflect weight loss occurring in the prodromal phase of dementia, which has been interpreted as reverse causation rather than a true protective effect [112,113]. Collectively, these results highlight that the role of central obesity may differ by outcome and age, and further longitudinal studies with repeated adiposity measures are needed to clarify these associations.

For visceral obesity, neither our meta-analysis nor MR analysis detected significant associations with cognitive disorders overall, suggesting that visceral adiposity may not exert a major causal effect on late-life cognitive health. This observation is in line with several previous studies. For instance, a cross-sectional study in China reported no significant relationship between visceral fat area and cognitive impairment [114], and a prospective cohort study in Iceland similarly found no association between visceral adiposity and dementia [115]. Other reports have produced mixed findings across different cognitive outcomes [116,117]. However, the current evidence base is limited by relatively small sample sizes and heterogeneous approaches to measuring visceral adiposity, ranging from bioimpedance to imaging-based assessments. Large-scale longitudinal studies using standardized imaging modalities are therefore needed to clarify whether visceral obesity contributes to specific types of cognitive disorders.

Our subgroup analyses further demonstrated important variations by gender, study design, geographic region, and type of cognitive disorder. Across both male and female participants, overweight and obesity were inversely associated with cognitive disorders overall, whereas underweight was consistently linked to increased risk. Notably, this protective pattern of overweight and obesity was most evident in cohort studies, whereas case-control studies showed more heterogeneous results, likely because of smaller sample sizes and methodological limitations. Regional analyses indicated that the inverse association of overweight and obesity with cognitive disorders was robust in Asian populations, but also observed in European and American cohorts, suggesting that ethnic and cultural differences in body composition and health behaviors may partly shape these relationships [118,119]. When stratified by outcome type, underweight was positively associated with dementia and AD, supporting the hypothesis that weight loss may occur during the preclinical stages of neurodegenerative disorders, possibly through dysfunction of brain regions involved in weight regulation, such as the mesial temporal cortex and hippocampus [120,121]. Conversely, overweight and obesity were inversely associated with dementia and AD, which could be partially explained by elevated circulating concentrations of leptin, a hormone secreted by adipose tissue that has been linked to reduced dementia risk [122]. Additionally, prior studies have indicated that overweight and obesity may be associated with lower mortality in older adults [123], which could further contribute to the apparent protective associations observed in our analyses. Taken together, these subgroup findings highlight that the impact of adiposity on late-life cognition is not uniform but may vary across populations and outcomes, underscoring the need for age- and disease-specific investigations.

To strengthen causal inference, we conducted a 2-sample MR analysis across distinct obesity phenotypes. Previous MR studies on obesity and cognitive impairment have shown that obesity may lead to cognitive and psychiatric disorders [21]. There is a causal relationship between obesity and brain abnormalities [22]. In recent years, research has also been conducted on the relationship between childhood obesity and AD [23]. However, in previous MR studies, the relationship between obesity types and cognitive impairment has not been systematically distinguished. Therefore, we conducted a 2-sample MR study to investigate the association between these 4 types of obesity and the risk of cognitive impairment. Our MR results suggested that genetically predicted general obesity (BMI) was inversely associated with cognitive impairment and MCI, but showed no clear relationship with dementia or AD. A similar pattern was observed for BFP, which was inversely associated with cognitive impairment but not with other outcomes. For central obesity, both WC and WHR showed inverse associations with cognitive impairment (and for WHR, also with MCI), whereas associations with dementia and AD were not evident. By contrast, VAT did not demonstrate convincing associations with any cognitive outcome. Taken together, these findings refine prior MR evidence by clarifying phenotype- and outcome-specific patterns: signals are most consistent for late-life cognitive impairment phenotypes, whereas evidence for dementia and AD remains limited. The areas of divergence between MR and our meta-analysis—particularly for central adiposity—likely reflect differences in study design and target estimands (lifelong, genetically proxied adiposity in MR compared with heterogeneous observational exposures in meta-analysis), population structure and age range, and remaining sources of bias discussed above. These considerations underscore the value of integrating MR with well-phenotyped longitudinal cohorts to reconcile phenotype definitions and endpoint specificity.

There are several mechanisms that may help to explain the association between various obesity phenotypes and cognitive disorders in older adults. Different neurobiological changes and cognitive outcomes may arise from different obesity phenotypes [16]. First, the discovery of the “obesity paradox” has revealed that in older adults, being overweight or obese is associated with a lower mortality risk compared with being underweight or having a normal weight [18]. Therefore, it appears that overweight or obesity may serve as an indicator of better health status in late life when compared with normal weight or underweight individuals [110]. Another hypothesis suggests that older individuals who are overweight or obese may have a more optimal intake of trace elements, vitamins, and other nutrients that enhance the functioning of molecular pathways involved in regulating cognitive [17]. Besides, various biological processes have elucidated that in late-life, overweight or obesity may confer protection by augmenting concentrations of insulin-like growth factor *I*, leptin hormone, and estrogen production [124,125], all of which have been demonstrated to be associated with enhanced cognitive performance. Second, central obesity and visceral obesity are associated with metabolic syndrome [125]. The impaired vascular function resulting from the different conditions of the metabolic syndrome could lead to brain changes, which may in turn cause cognitive impairment [111]. Therefore, the underlying mechanisms connecting obesity, metabolic syndrome, cognition disorders, and brain changes necessitate further investigation into diverse obesity phenotypes.

Interestingly, the discrepancies observed between our meta-analysis and MR results deserve particular attention. Meta-analyses combine heterogeneous observational studies that differ in population characteristics, follow-up durations, and definitions of obesity, which may obscure true associations with cognitive disorders. Observational studies are also subject to residual confounding, reverse causation, and measurement error, whereas MR analyses, although less affected by these issues, are not immune to horizontal pleiotropy. Moreover, MR estimates capture the lifelong effects of genetically predicted adiposity, which may represent different biological processes than those reflected by anthropometric measures in late life. Lifestyle factors and comorbidities could further modify or mask associations in traditional studies. Taken together, these differences underscore the complementary strengths of meta-analysis and MR, and highlight the importance of future research that integrates harmonized phenotyping, longitudinal designs, and multiethnic cohorts to better define the role of different obesity phenotypes in cognitive disorders.

This study has several notable strengths. First, to our knowledge, it is the first meta-analysis to comprehensively evaluate the associations of 3 major obesity phenotypes—general, central, and visceral obesity—with cognitive disorders in older adults. By incorporating an extensive body of literature, the meta-analysis not only broadened the sample base but also performed multiple subgroup analyses, thereby enhancing the robustness and interpretability of the findings. Second, we complemented the observational synthesis with a 2-sample MR analysis, which allowed us to further test causal relationships between obesity phenotypes and cognitive outcomes. In this context, instrumental variables were derived from the largest available GWAS, providing optimal sample size, instrument strength, and statistical power. Together, the integration of meta-analysis and MR constitutes a comprehensive approach, yielding more reliable and phenotype-specific insights into the relationship between adiposity and late-life cognition.

Several limitations of this study should be acknowledged. First, many observational studies included in the meta-analysis were vulnerable to biases such as selection bias, recall bias, and random error because of their uncontrolled design. Although various confounding factors have been adjusted, it is still impossible to completely eliminate the influence of residual confounding factors, especially in terms of exercise, smoking, and drinking. Second, the coexistence and overlap of different obesity phenotypes may complicate attribution of specific effects. Third, exclusion of non-English publications may have introduced language bias. Fourth, the evidence base for central obesity (especially WHR), visceral obesity, and BFP was relatively limited. For BFP in particular, the number of available studies was small and heterogeneity was high, meaning that conclusions must be drawn with caution, and additional research is needed to clarify these associations. Fifth, because our meta-analysis focused on the elderly population, these research results may not be applicable to younger or middle-aged individuals. Prior evidence, including the 2024 Lancet dementia report [126], indicates that midlife obesity increases the risk of later cognitive decline and dementia, suggesting life-course heterogeneity. Future work should incorporate age-stratified, multiancestry GWAS datasets and longitudinal cohorts spanning diverse life stages to validate and extend these findings. Sixth, regarding the MR analysis, several additional caveats apply. At present, there are no large-scale GWAS that directly use “cognitive impairment” or “MCI” as phenotypes. Therefore, we have to adopt 2 closely related surrogate phenotypes, cognitive function and cognitive performance, to jointly define the genetic susceptibility of cognitive impairment. Although this study used 2 related variables, the interpretation of the results still requires careful consideration. Future research (once there is comprehensive GWAS data specifically targeting the clinical diagnosis of cognitive impairment) will be of great value in verifying our conclusions. Seventh, in our outcome assessment, we did not include the mortality rate related to dementia because the data on the causes of death are inconsistent, and there may be cases of incorrect classification. Furthermore, GWAS have not yet produced a standardized summary statistical result regarding the mortality rate associated with dementia. This exclusion might lead to an underestimation of the burden of serious consequences. Future research should aim to fill in these gaps in this study. Eighth, our MR analyses were also restricted to individuals of European ancestry and were not stratified by age, limiting generalizability to other populations and to older adults. Differences in genetic background to cognitive decline across populations highlight the need for caution in extrapolation.

In conclusion, both meta-analysis and MR evidence suggest that overweight and obesity are associated with a lower risk of cognitive impairment in older adults, whereas underweight is linked to a higher risk. MR analyses further confirmed inverse associations of BMI, WC, and WHR with cognitive impairment, whereas no causal effect was observed for visceral obesity. Clinical significance remains to be determined. These findings highlight phenotype-specific patterns and underscore the need for further research to clarify mechanisms and life-course heterogeneity.

## Author contributions

The authors’ responsibilities were as follows – DL, XJ: conceptualization, formal analysis, visualization, and writing-original draft; YA: conceptualization, methodology, data curation, and writing-original draft; HL, GZ, HY, YZ: writing-review and editing; YL: supervision, validation, and writing-review and editing; LZ: resources; data curation, writing–review and editing, and funding acquisition; YX: conceptualization, resources, and writing-review and editing, supervision, funding acquisition; and all authors: read and approved the final manuscript.

## Ethics approval

The National Health Service National Research Ethics Service (21/NW/0157).

## Consent to participate

Written informed consent was obtained from each participant before enrollment.

## Funding

Supported by the Young Elite Scientists Sponsorship Program by China Association for Science and Technology (grant number 2020QNRC001 to Yang Xia), the LiaoNing Revitalization Talents Program (grant number XLYC2203168 to Yang Xia), the Scientific Research Project of the Liaoning Province Education Department (grant number LJKMZ20221149 to Yang Xia), and the General Program of Natural Science Foundation of Liaoning Province (grant numbers 2025-MS-221 to Yang Xia).

## Conflict of interest

The authors report no conflicts of interest.
