# Supplementary material for: The Associations of General, Central, Visceral Obesity, and Body Fat Percentage with Cognitive Impairment in the Elderly: Meta-analysis and Mendelian Randomization Study
Source: Adv Nutr. 2025 Nov 3;16(12):100550. doi: 10.1016/j.advnut.2025.100550 (PMC12677176; doi:10.1016/j.advnut.2025.100550)
Supplement: Multimedia component 1 [file mmc1.docx]

Title: The Associations of General, Central, Visceral Obesity and Body Fat Percentage with Cognitive Impairment in the Elderly: Meta-analysis and Mendelian Randomization Study

First Author: Duoduo Lu

Supplementary Table 1. Search strategy

Supplementary Table 2. Studies with sex-specific but no overall effect estimates

Supplementary Table 3. Genetic variables in MR analysis

Supplementary Table 4. Sensitivity analysis results of the correlation between BMI, %BF, WC, WHR, and cognitive impairment

Supplementary Table 5. Details of GWAS data on exposure and outcome

Supplementary Table 6. Characteristics of studies included

Supplementary Table 7. Risk of bias assessment (Newcastle-Ottawa Quality Assessment Scale criteria)

Supplementary Figure 1. Sensitivity analysis of association between General, Central, Visceral Obesity and Body Fat Percentage with Cognitive disorders

Supplementary Figure 2. Funnel plot for assessment of publication bias

Supplementary Figure 3. Egger test for identifying publication bias

Supplementary Figure 4. Scatter plot of the causal relationship between general obesity, central obesity, and visceral obesity and cognitive impairment

Supplementary Figure 5. A funnel plot of the causal relationship between general obesity, central obesity, and visceral obesity and cognitive impairment

Supplementary Figure 6. Leave-one-out sensitivity analysis of MR for general obesity, central obesity, and visceral obesity and cognitive impairment

Supplementary Figure 7. Detailed results of sensitivity analysis on MR

Supplementary Table 1. Search strategy

| PubMed: | |
| --- | --- |
| #1 | (“Body Mass Index” OR “BMI” OR “body mass indices” OR “overweight” OR “obesity” OR “underweight” OR “abdominal fatness” OR “abdominal obesity” OR “waist circumference” OR “central obesity” OR “visceral obesity” OR “central adiposity” OR “waist circumference” OR “waist-to-hip ratio” OR “waist-hip ratio” OR “visceral fat” OR “excessive fat” OR “abdominal fat” OR “visceral adipose tissue” OR “visceral adiposity” OR “central adiposity” OR “subcutaneous fat” OR “Body fat percentage”) |
| #2 | (“Cognition” OR “cognitive” OR “mild cognitive impairment” OR “cognitive decline” OR “cognitive impairment” OR “cognitive defect” OR “cognitive dysfunction” OR “cognitive disorder” OR cognit* OR “neurocognitive function” OR “brain funct*” OR dementia OR “Alzheimer’s disease” OR “Alzheimer disease” OR “Alzheimer*”) |
| #3 | “Animals” |
| #4 | #1 AND #2 NOT #3 |
| Web of Science databases: | |
| #1 | TI = (“Body Mass Index” OR “BMI” OR “body mass indices” OR “overweight” OR “obesity” OR “underweight” OR “abdominal fatness” OR “abdominal obesity” OR “waist circumference” OR “central obesity” OR “visceral obesity” OR “central adiposity” OR “waist circumference” OR “waist-to-hip ratio” OR “waist-hip ratio” OR “visceral fat” OR “excessive fat” OR “abdominal fat” OR “visceral adipose tissue” OR “visceral adiposity” OR “central adiposity” OR “subcutaneous fat” OR “Body fat percentage”) |
| #2 | TI = (“Cognition” OR “cognitive” OR “mild cognitive impairment” OR “cognitive decline” OR “cognitive impairment” OR “cognitive defect” OR “cognitive dysfunction” OR “cognitive disorder” OR cognit* OR “neurocognitive function” OR “brain funct*” OR “dementia” OR “Alzheimer’s disease” OR “Alzheimer disease” OR “Alzheimer*”) |
| #3 | TI = “Animals” |
| #4 | #1 AND #2 NOT #3 |
| EMBASE: | |
| #1 | (‘Body Mass Index’ OR ‘BMI’ OR ‘body mass indices’ OR ‘overweight’ OR ‘obesity’ OR ‘underweight’ OR ‘abdominal fatness’ OR ‘abdominal obesity’ OR ‘waist circumference’ OR ‘central obesity’ OR ‘visceral obesity’ OR ‘central adiposity’ OR ‘waist circumference’ OR ‘waist-to-hip ratio’ OR ‘waist-hip ratio’ OR ‘visceral fat’ OR ‘excessive fat’ OR ‘abdominal fat’ OR ‘visceral adipose tissue’ OR ‘visceral adiposity’ OR ‘central adiposity’ OR ‘subcutaneous fat’ OR ‘Body fat percentage’) |
| #2 | (‘Cognition’ OR ‘cognitive’ OR ‘mild cognitive impairment’ OR ‘cognitive decline’ OR ‘cognitive impairment’ OR ‘cognitive defect’ OR ‘cognitive dysfunction’ OR ‘cognitive disorder’ OR cognit* OR ‘neurocognitive function’ OR ‘brain funct*’ OR dementia OR ‘alzheimer disease’ OR ‘alzheimer’) |
| #3 | ‘Animals’ |
| #4 | #1 AND #2 NOT #3 |
| MEDLINE: | |
| #1 | TI = (“Body Mass Index” OR “BMI” OR “body mass indices” OR “overweight” OR “obesity” OR “underweight” OR “abdominal fatness” OR “abdominal obesity” OR “waist circumference” OR “central obesity” OR “visceral obesity” OR “central adiposity” OR “waist circumference” OR “waist-to-hip ratio” OR “waist-hip ratio” OR “visceral fat” OR “excessive fat” OR “abdominal fat” OR “visceral adipose tissue” OR “visceral adiposity” OR “central adiposity” OR “subcutaneous fat” OR “Body fat percentage”) |
| #2 | TI = (“Cognition” OR “cognitive” OR “mild cognitive impairment” OR “cognitive decline” OR “cognitive impairment” OR “cognitive defect” OR “cognitive dysfunction” OR “cognitive disorder” OR cognit* OR “neurocognitive function” OR “brain funct*” OR dementia OR “Alzheimer’s disease” OR “Alzheimer disease” OR “Alzheimer*”) |
| #3 | TI = “Animals” |
| #4 | #1 AND #2 NOT #3 |

Supplementary Table 2. Studies with sex-specific but no overall effect estimates

| Author | Exposure | Outcome | Subgroup effect/male | SE/male | Subgroup effect/female | SE/female | Overall OR (95% CI) |
| --- | --- | --- | --- | --- | --- | --- | --- |
| Lin WY | Body fat percentage | Cognitive impairment | 1.11 (0.96, 1.29) | 0.077 | 1.04 (0.92, 1.17) | 0.060 | 1.06 (0.97, 1.17) |
| Hiroshi Yokomichi | Obesity | Dementia | 0.91 (0.13, 6.64) | 1.003 | 0.61 (0.09, 4.43) | 0.994 | 0.74 (0.19, 2.97) |
| Hiroshi Yokomichi | Overweight | Dementia | 0.73 (0.42, 1.28) | 0.284 | 0.82 (0.49, 1.37) | 0.262 | 0.78 (0.53, 1.13) |
| Hiroshi Yokomichi | Underweight | Dementia | 1.04 (0.51, 2.10) | 0.361 | 1.72 (1.05, 2.81) | 0.251 | 1.43 (0.89, 2.30) |

Supplementary Table 3. Genetic variables in MR analysis

| 508 SNPs are associated with Body Mass Index | | | | | | | | |
| --- | --- | --- | --- | --- | --- | --- | --- | --- |
|  | | | | | | | | |
| SNP | chr.exposure | effect_allele.exposure | other_allele.exposure | beta.exposure | eaf.exposure | pval.exposure | se.exposure | samplesize.exposure |
| rs12044597 | 1 | G | A | 0.0143 | 0.5029 | 1.70E-18 | 0.0016 | 789125 |
| rs2284746 | 1 | G | C | -0.0104 | 0.5238 | 1.40E-09 | 0.0017 | 692206 |
| rs6692586 | 1 | G | A | -0.0192 | 0.832 | 1.10E-16 | 0.0023 | 690921 |
| rs705217 | 1 | G | T | -0.0102 | 0.3652 | 9.30E-09 | 0.0018 | 688609 |
| rs4660443 | 1 | T | C | 0.0164 | 0.2218 | 6.80E-15 | 0.0021 | 687234 |
| rs17014375 | 1 | G | T | 0.0172 | 0.1348 | 1.10E-11 | 0.0025 | 690856 |
| rs7535528 | 1 | A | G | -0.0152 | 0.3741 | 1.40E-16 | 0.0018 | 632868 |
| rs2235564 | 1 | T | C | 0.0131 | 0.3466 | 3.70E-13 | 0.0018 | 691544 |
| rs10914462 | 1 | G | A | -0.0112 | 0.4255 | 1.50E-10 | 0.0017 | 689808 |
| rs12033257 | 1 | G | A | -0.0146 | 0.3835 | 2.40E-15 | 0.0018 | 664083 |
| rs6587552 | 1 | G | A | -0.0173 | 0.7591 | 1.60E-17 | 0.002 | 689723 |
| rs6673081 | 1 | C | T | -0.01 | 0.5534 | 1.80E-08 | 0.0018 | 677818 |
| rs543874 | 1 | G | A | 0.0475 | 0.1952 | 1.20E-122 | 0.002 | 795504 |
| rs11118308 | 1 | G | A | -0.0101 | 0.4703 | 4.80E-10 | 0.0016 | 794625 |
| rs17425707 | 1 | C | T | 0.0167 | 0.1003 | 4.40E-09 | 0.0028 | 688867 |
| rs6593688 | 1 | G | A | 0.0137 | 0.3733 | 8.60E-15 | 0.0018 | 691779 |
| rs946824 | 1 | C | T | -0.0206 | 0.859 | 1.10E-15 | 0.0026 | 689849 |
| rs1891216 | 1 | G | T | 0.0107 | 0.3759 | 2.40E-09 | 0.0018 | 685079 |
| rs2791653 | 1 | G | A | -0.0141 | 0.7577 | 1.30E-13 | 0.0019 | 795271 |
| rs657452 | 1 | G | A | -0.0188 | 0.6216 | 7.20E-29 | 0.0017 | 767846 |
| rs2481665 | 1 | C | T | -0.0161 | 0.4408 | 7.20E-23 | 0.0016 | 795247 |
| rs7519259 | 1 | A | G | 0.0125 | 0.5356 | 3.80E-13 | 0.0017 | 681362 |
| rs7551507 | 1 | T | C | -0.0184 | 0.5633 | 9.30E-30 | 0.0016 | 794579 |
| rs12049202 | 1 | T | C | 0.024 | 0.203 | 1.00E-28 | 0.0022 | 691566 |
| rs818524 | 1 | C | T | 0.0106 | 0.6939 | 3.40E-08 | 0.0019 | 670078 |
| rs2007231 | 1 | T | C | -0.0104 | 0.6387 | 5.20E-09 | 0.0018 | 691969 |
| rs10733051 | 1 | G | A | -0.0097 | 0.4802 | 2.90E-09 | 0.0016 | 781928 |
| rs12564992 | 1 | G | A | 0.0196 | 0.1144 | 5.30E-14 | 0.0026 | 795119 |
| rs10915840 | 1 | A | G | -0.0118 | 0.283 | 1.30E-09 | 0.0019 | 684857 |
| rs4653017 | 1 | T | C | 0.0122 | 0.6818 | 4.50E-11 | 0.0018 | 686378 |
| rs977747 | 1 | G | T | -0.0169 | 0.5949 | 1.30E-24 | 0.0017 | 793546 |
| rs1993709 | 1 | G | A | 0.0331 | 0.8177 | 1.90E-57 | 0.0021 | 786001 |
| rs11165643 | 1 | T | C | 0.0206 | 0.5828 | 1.40E-35 | 0.0017 | 792657 |
| rs10747488 | 1 | A | C | -0.0123 | 0.7601 | 1.20E-09 | 0.002 | 689295 |
| rs11185111 | 1 | A | G | -0.0129 | 0.3042 | 7.70E-12 | 0.0019 | 686508 |
| rs7550711 | 1 | T | C | 0.0649 | 0.03058 | 3.20E-38 | 0.005 | 769184 |
| rs4414033 | 1 | A | G | 0.0129 | 0.627 | 1.40E-12 | 0.0018 | 672697 |
| rs10920678 | 1 | G | A | -0.0155 | 0.5709 | 1.50E-21 | 0.0016 | 788624 |
| rs12041258 | 1 | C | T | -0.0146 | 0.2287 | 9.50E-13 | 0.002 | 688602 |
| rs2820311 | 1 | G | A | 0.0235 | 0.3369 | 4.10E-38 | 0.0018 | 691876 |
| rs16849710 | 1 | G | A | -0.0116 | 0.515 | 6.00E-11 | 0.0018 | 666229 |
| rs2693826 | 2 | A | G | -0.0137 | 0.4421 | 2.00E-15 | 0.0017 | 690808 |
| rs4358081 | 2 | C | A | 0.0097 | 0.4631 | 1.50E-08 | 0.0017 | 690038 |
| rs6545714 | 2 | A | G | -0.0191 | 0.6139 | 9.10E-31 | 0.0017 | 793368 |
| rs1371108 | 2 | A | C | 0.0119 | 0.3247 | 9.00E-11 | 0.0018 | 684620 |
| rs4851029 | 2 | G | T | 0.0121 | 0.5247 | 1.70E-12 | 0.0017 | 689752 |
| rs10192119 | 2 | G | T | 0.0166 | 0.1673 | 3.00E-14 | 0.0022 | 795369 |
| rs1521527 | 2 | C | G | -0.0121 | 0.5324 | 3.10E-12 | 0.0017 | 678175 |
| rs17499593 | 2 | G | C | 0.0125 | 0.1897 | 1.10E-08 | 0.0022 | 691663 |
| rs1064213 | 2 | A | G | 0.012 | 0.492 | 2.40E-12 | 0.0017 | 692576 |
| rs4639527 | 2 | G | A | 0.0172 | 0.3012 | 3.30E-20 | 0.0019 | 691706 |
| rs13021737 | 2 | G | A | 0.0574 | 0.8319 | 7.50E-157 | 0.0021 | 789534 |
| rs10169594 | 2 | C | T | 0.0121 | 0.3596 | 2.00E-11 | 0.0018 | 685712 |
| rs4556997 | 2 | A | C | 0.0197 | 0.1349 | 6.90E-17 | 0.0024 | 792972 |
| rs429343 | 2 | G | A | -0.015 | 0.5813 | 6.80E-18 | 0.0017 | 689345 |
| rs3764835 | 2 | A | G | -0.0141 | 0.1528 | 3.10E-09 | 0.0024 | 689505 |
| rs3754963 | 2 | T | A | -0.0123 | 0.2574 | 3.30E-10 | 0.002 | 691535 |
| rs12328930 | 2 | C | T | 0.0098 | 0.4235 | 1.80E-08 | 0.0017 | 690521 |
| rs1528435 | 2 | T | C | 0.0164 | 0.6331 | 9.10E-23 | 0.0017 | 794198 |
| rs3731695 | 2 | C | T | 0.0116 | 0.5582 | 7.90E-13 | 0.0016 | 793581 |
| rs7599312 | 2 | A | G | -0.0186 | 0.2652 | 6.90E-24 | 0.0019 | 780823 |
| rs1260326 | 2 | C | T | 0.0105 | 0.5973 | 3.90E-10 | 0.0017 | 784462 |
| rs17399237 | 2 | C | T | -0.0129 | 0.5497 | 6.70E-14 | 0.0017 | 690950 |
| rs930295 | 2 | C | A | -0.0211 | 0.8417 | 1.00E-19 | 0.0023 | 690522 |
| rs4671328 | 2 | G | T | -0.0219 | 0.5533 | 2.20E-36 | 0.0017 | 679487 |
| rs7557796 | 2 | C | T | -0.016 | 0.6524 | 2.30E-19 | 0.0018 | 692414 |
| rs10197031 | 2 | C | T | 0.0166 | 0.2834 | 1.90E-18 | 0.0019 | 691479 |
| rs1445652 | 2 | A | G | 0.0123 | 0.1855 | 4.30E-08 | 0.0022 | 682166 |
| rs4482463 | 2 | A | C | -0.0331 | 0.9213 | 2.80E-23 | 0.0033 | 635414 |
| rs3732084 | 2 | C | T | 0.0107 | 0.6139 | 1.10E-09 | 0.0018 | 691763 |
| rs2162524 | 2 | C | T | 0.0155 | 0.3321 | 4.10E-17 | 0.0018 | 691302 |
| rs10182181 | 2 | G | A | 0.0325 | 0.4753 | 6.70E-90 | 0.0016 | 792111 |
| rs902695 | 2 | A | G | -0.0103 | 0.4798 | 2.20E-09 | 0.0017 | 678635 |
| rs4954638 | 2 | C | A | -0.0118 | 0.2492 | 2.90E-09 | 0.002 | 689971 |
| rs4589691 | 2 | G | C | 0.0141 | 0.1579 | 4.70E-09 | 0.0024 | 688253 |
| rs11889536 | 2 | G | A | -0.0189 | 0.1493 | 6.40E-15 | 0.0024 | 688977 |
| rs934224 | 2 | T | C | 0.0107 | 0.7399 | 4.70E-08 | 0.002 | 692568 |
| rs4952843 | 2 | G | A | -0.0131 | 0.3807 | 6.80E-14 | 0.0018 | 692482 |
| rs3806572 | 2 | A | G | -0.0145 | 0.2788 | 1.60E-14 | 0.0019 | 687646 |
| rs2861683 | 2 | C | A | -0.0144 | 0.407 | 1.30E-16 | 0.0017 | 691163 |
| rs17551974 | 2 | A | C | -0.0141 | 0.1782 | 1.90E-10 | 0.0022 | 691115 |
| rs17203016 | 2 | G | A | 0.015 | 0.196 | 2.10E-13 | 0.002 | 786272 |
| rs199783965 | 2 | T | C | 0.0156 | 0.3461 | 6.50E-18 | 0.0018 | 680852 |
| rs2317299 | 2 | C | T | -0.0106 | 0.5597 | 1.30E-09 | 0.0017 | 677983 |
| rs17033117 | 3 | T | C | 0.0137 | 0.1872 | 8.90E-10 | 0.0022 | 691863 |
| rs11713193 | 3 | A | G | 0.0239 | 0.5073 | 2.40E-44 | 0.0017 | 692159 |
| rs1452075 | 3 | T | C | 0.0141 | 0.7277 | 1.30E-14 | 0.0018 | 783729 |
| rs1006896 | 3 | C | A | -0.0234 | 0.1061 | 5.50E-18 | 0.0027 | 691892 |
| rs645040 | 3 | T | G | 0.0171 | 0.7762 | 2.50E-18 | 0.002 | 795579 |
| rs6443750 | 3 | C | T | 0.0148 | 0.8068 | 3.20E-12 | 0.0021 | 776837 |
| rs9816226 | 3 | T | A | 0.0323 | 0.8199 | 1.60E-52 | 0.0021 | 778333 |
| rs1552893 | 3 | G | A | -0.0126 | 0.2777 | 8.10E-11 | 0.0019 | 691313 |
| rs6764533 | 3 | A | G | 0.0116 | 0.359 | 1.40E-10 | 0.0018 | 690832 |
| rs17535749 | 3 | A | G | 0.015 | 0.1023 | 2.50E-08 | 0.0027 | 777038 |
| rs754635 | 3 | G | C | 0.0198 | 0.8873 | 2.20E-13 | 0.0027 | 690346 |
| rs7626079 | 3 | T | C | 0.011 | 0.3434 | 1.60E-09 | 0.0018 | 692571 |
| rs1656377 | 3 | C | T | 0.0099 | 0.5885 | 1.60E-08 | 0.0017 | 691955 |
| rs13069244 | 3 | A | G | 0.0187 | 0.07747 | 3.00E-09 | 0.0032 | 791327 |
| rs2600226 | 3 | T | C | -0.0116 | 0.6697 | 3.70E-10 | 0.0019 | 685285 |
| rs9845966 | 3 | G | T | -0.0105 | 0.5479 | 2.50E-10 | 0.0017 | 778076 |
| rs4858193 | 3 | C | T | -0.0129 | 0.2779 | 1.60E-11 | 0.0019 | 686850 |
| rs33500 | 3 | T | C | -0.0167 | 0.8082 | 4.30E-14 | 0.0022 | 689500 |
| rs538579 | 3 | C | G | 0.0137 | 0.3228 | 1.30E-13 | 0.0019 | 688452 |
| rs3772882 | 3 | A | C | 0.0127 | 0.3661 | 6.60E-13 | 0.0018 | 691912 |
| rs7640424 | 3 | T | C | -0.0136 | 0.2969 | 2.30E-14 | 0.0018 | 790612 |
| rs2868975 | 3 | A | G | -0.0143 | 0.178 | 2.20E-10 | 0.0023 | 690613 |
| rs8192675 | 3 | C | T | 0.0152 | 0.2888 | 1.40E-17 | 0.0018 | 795515 |
| rs10510419 | 3 | T | G | -0.0177 | 0.1416 | 2.20E-14 | 0.0023 | 789318 |
| rs2365389 | 3 | T | C | -0.0174 | 0.4143 | 1.30E-25 | 0.0017 | 783625 |
| rs6785245 | 3 | C | T | 0.0132 | 0.3969 | 4.00E-14 | 0.0017 | 692250 |
| rs12629015 | 3 | G | A | -0.0135 | 0.1852 | 2.10E-09 | 0.0023 | 691059 |
| rs1320903 | 3 | A | G | 0.0216 | 0.3174 | 9.20E-32 | 0.0018 | 691519 |
| rs16851483 | 3 | T | G | 0.0369 | 0.06928 | 3.20E-26 | 0.0035 | 692316 |
| rs7615297 | 3 | G | C | -0.0149 | 0.1465 | 5.70E-10 | 0.0024 | 689710 |
| rs6804842 | 3 | G | A | 0.0156 | 0.572 | 3.60E-21 | 0.0017 | 789179 |
| rs7637852 | 3 | G | A | -0.0139 | 0.6951 | 1.70E-13 | 0.0019 | 691815 |
| rs2325036 | 3 | C | A | -0.0181 | 0.3845 | 3.60E-27 | 0.0017 | 790870 |
| rs1454687 | 3 | G | C | -0.0202 | 0.5227 | 5.20E-32 | 0.0017 | 692324 |
| rs1436344 | 3 | C | G | 0.0141 | 0.5922 | 4.10E-16 | 0.0017 | 692017 |
| rs2124499 | 3 | C | G | -0.0123 | 0.3718 | 3.40E-13 | 0.0017 | 785955 |
| rs355777 | 3 | C | G | 0.0153 | 0.4106 | 1.40E-18 | 0.0017 | 689978 |
| rs1476322 | 3 | A | G | 0.0101 | 0.569 | 5.00E-09 | 0.0017 | 692523 |
| rs6772756 | 3 | G | A | -0.0104 | 0.3372 | 4.00E-08 | 0.0019 | 681709 |
| rs865809 | 3 | G | A | -0.0127 | 0.7678 | 5.40E-10 | 0.002 | 689186 |
| rs10938397 | 4 | G | A | 0.0324 | 0.4317 | 3.40E-86 | 0.0016 | 793518 |
| rs326896 | 4 | T | C | -0.0128 | 0.3925 | 2.80E-13 | 0.0018 | 690324 |
| rs4864201 | 4 | C | T | -0.0141 | 0.6469 | 1.50E-16 | 0.0017 | 795263 |
| rs331966 | 4 | C | A | 0.0112 | 0.3792 | 3.20E-10 | 0.0018 | 687189 |
| rs11736228 | 4 | T | A | -0.0139 | 0.2587 | 4.10E-12 | 0.002 | 691580 |
| rs1492767 | 4 | T | C | 0.0094 | 0.4957 | 1.00E-08 | 0.0016 | 794161 |
| rs7685048 | 4 | T | C | -0.0101 | 0.4654 | 4.10E-09 | 0.0017 | 692398 |
| rs13107325 | 4 | T | C | 0.047 | 0.07373 | 1.10E-47 | 0.0032 | 792045 |
| rs2051559 | 4 | C | T | 0.0176 | 0.1308 | 5.00E-12 | 0.0026 | 689307 |
| rs13132853 | 4 | G | A | -0.0142 | 0.3452 | 4.70E-15 | 0.0018 | 682543 |
| rs10009336 | 4 | T | C | -0.014 | 0.1638 | 2.20E-10 | 0.0022 | 794766 |
| rs6815910 | 4 | A | T | -0.0128 | 0.5435 | 1.40E-13 | 0.0017 | 689263 |
| rs17001561 | 4 | A | G | 0.0151 | 0.1573 | 3.80E-11 | 0.0023 | 794327 |
| rs13147390 | 4 | C | T | 0.0103 | 0.3569 | 1.00E-08 | 0.0018 | 681032 |
| rs4148155 | 4 | G | A | -0.0188 | 0.1127 | 5.00E-13 | 0.0026 | 794889 |
| rs7694732 | 4 | G | A | -0.0099 | 0.4378 | 8.70E-09 | 0.0017 | 690622 |
| rs1296328 | 4 | C | A | -0.0179 | 0.5657 | 4.90E-24 | 0.0018 | 683488 |
| rs13110266 | 4 | A | G | -0.0117 | 0.4065 | 1.90E-12 | 0.0017 | 791087 |
| rs1522569 | 4 | G | T | -0.0164 | 0.1819 | 2.90E-13 | 0.0022 | 689573 |
| rs2643452 | 4 | A | T | 0.0136 | 0.5448 | 4.70E-15 | 0.0017 | 690573 |
| rs6448587 | 4 | C | A | -0.0167 | 0.1891 | 2.30E-13 | 0.0023 | 691097 |
| rs11945861 | 4 | A | G | -0.0148 | 0.2369 | 5.00E-13 | 0.002 | 682451 |
| rs1804528 | 4 | A | G | 0.0109 | 0.3507 | 3.00E-08 | 0.002 | 518856 |
| rs6841761 | 4 | T | G | -0.0131 | 0.5252 | 6.40E-16 | 0.0016 | 793477 |
| rs1863652 | 4 | A | G | -0.0115 | 0.3449 | 1.40E-10 | 0.0018 | 692539 |
| rs7683836 | 4 | A | G | -0.0114 | 0.5405 | 6.30E-11 | 0.0017 | 686968 |
| rs7730004 | 5 | T | C | 0.0148 | 0.6693 | 9.10E-16 | 0.0018 | 690164 |
| rs17424296 | 5 | A | G | -0.0108 | 0.3659 | 2.40E-09 | 0.0018 | 684366 |
| rs2931434 | 5 | T | C | -0.0104 | 0.3168 | 1.40E-08 | 0.0018 | 690664 |
| rs13174863 | 5 | G | A | 0.0192 | 0.1548 | 2.90E-16 | 0.0023 | 773762 |
| rs3844598 | 5 | G | A | 0.0095 | 0.521 | 3.80E-08 | 0.0017 | 690704 |
| rs2367112 | 5 | G | T | -0.0119 | 0.4919 | 2.30E-13 | 0.0016 | 794305 |
| rs16903285 | 5 | C | T | 0.0331 | 0.1407 | 7.60E-38 | 0.0026 | 687944 |
| rs11739877 | 5 | T | C | 0.0117 | 0.6118 | 6.60E-11 | 0.0018 | 692540 |
| rs6595205 | 5 | G | C | -0.0114 | 0.5305 | 2.00E-12 | 0.0016 | 794525 |
| rs7715256 | 5 | T | G | -0.0166 | 0.5781 | 2.20E-24 | 0.0016 | 795302 |
| rs2307111 | 5 | C | T | -0.0265 | 0.3962 | 1.60E-58 | 0.0016 | 795430 |
| rs6235 | 5 | G | C | 0.0175 | 0.2702 | 1.50E-19 | 0.0019 | 691708 |
| rs11951673 | 5 | T | C | -0.0123 | 0.3941 | 1.10E-13 | 0.0017 | 792278 |
| rs10478110 | 5 | C | A | 0.01 | 0.4348 | 9.60E-09 | 0.0017 | 680441 |
| rs13184896 | 5 | T | G | -0.0133 | 0.4346 | 3.30E-16 | 0.0016 | 794825 |
| rs7724675 | 5 | A | G | -0.0119 | 0.2238 | 9.50E-09 | 0.0021 | 691968 |
| rs7703576 | 5 | C | T | 0.0103 | 0.2885 | 4.80E-08 | 0.0019 | 690818 |
| rs16871902 | 5 | A | G | 0.0125 | 0.4877 | 4.60E-13 | 0.0017 | 690830 |
| rs7704281 | 5 | A | G | 0.0271 | 0.04531 | 6.50E-11 | 0.0041 | 788585 |
| rs1503526 | 5 | C | T | 0.014 | 0.4838 | 5.50E-17 | 0.0017 | 747347 |
| rs2009416 | 5 | T | C | -0.0121 | 0.361 | 1.10E-11 | 0.0018 | 691741 |
| rs40067 | 5 | A | G | -0.0266 | 0.1713 | 7.10E-30 | 0.0023 | 681695 |
| rs294704 | 5 | T | G | -0.0113 | 0.7239 | 4.00E-09 | 0.0019 | 690036 |
| rs4518345 | 5 | A | G | -0.0117 | 0.2842 | 1.00E-09 | 0.0019 | 688609 |
| rs876605 | 5 | G | A | -0.0108 | 0.7352 | 3.40E-08 | 0.002 | 692586 |
| rs10942267 | 5 | G | A | -0.0156 | 0.3088 | 3.90E-17 | 0.0019 | 689084 |
| rs11738695 | 5 | A | C | 0.0097 | 0.586 | 2.00E-08 | 0.0017 | 691380 |
| rs17056301 | 5 | C | T | 0.0118 | 0.2636 | 2.40E-09 | 0.002 | 688085 |
| rs189843 | 5 | C | G | -0.0098 | 0.5557 | 1.70E-08 | 0.0017 | 685314 |
| rs17663412 | 5 | A | C | 0.0157 | 0.1139 | 6.10E-09 | 0.0027 | 691018 |
| rs7730898 | 5 | A | G | 0.0168 | 0.729 | 4.50E-20 | 0.0018 | 792975 |
| rs806600 | 5 | G | A | -0.0095 | 0.475 | 3.30E-08 | 0.0017 | 691791 |
| rs6556301 | 5 | T | G | -0.0111 | 0.3596 | 4.10E-10 | 0.0018 | 734744 |
| rs2228213 | 6 | A | G | -0.0139 | 0.3481 | 4.60E-16 | 0.0017 | 795595 |
| rs9367368 | 6 | C | T | -0.0121 | 0.3033 | 1.00E-11 | 0.0018 | 786723 |
| rs9379827 | 6 | A | C | -0.0132 | 0.2409 | 6.90E-12 | 0.0019 | 795072 |
| rs9370261 | 6 | T | C | 0.0231 | 0.04601 | 3.40E-08 | 0.0042 | 690051 |
| rs947612 | 6 | A | G | -0.0116 | 0.7516 | 5.60E-09 | 0.002 | 692596 |
| rs2246012 | 6 | C | T | 0.0158 | 0.1628 | 3.10E-13 | 0.0022 | 795598 |
| rs1885728 | 6 | A | G | 0.0108 | 0.6787 | 1.00E-08 | 0.0019 | 682316 |
| rs3828783 | 6 | A | G | -0.0165 | 0.1809 | 5.60E-15 | 0.0021 | 792749 |
| rs2744974 | 6 | T | C | 0.0249 | 0.338 | 1.40E-45 | 0.0018 | 789647 |
| rs765875 | 6 | T | C | -0.0121 | 0.4808 | 3.00E-12 | 0.0017 | 690961 |
| rs3806114 | 6 | A | G | -0.0113 | 0.6773 | 3.40E-10 | 0.0018 | 778855 |
| rs987237 | 6 | G | A | 0.0409 | 0.1803 | 9.30E-84 | 0.0021 | 795612 |
| rs1327259 | 6 | G | A | -0.0155 | 0.3872 | 1.70E-18 | 0.0018 | 685922 |
| rs9688431 | 6 | C | T | -0.0231 | 0.06034 | 2.40E-11 | 0.0035 | 789356 |
| rs9294260 | 6 | A | G | 0.0147 | 0.4731 | 1.80E-19 | 0.0016 | 783533 |
| rs2357760 | 6 | A | G | 0.0145 | 0.6754 | 6.80E-17 | 0.0017 | 791053 |
| rs2875762 | 6 | C | G | 0.0139 | 0.2473 | 1.20E-11 | 0.002 | 685199 |
| rs1268065 | 6 | A | G | -0.0102 | 0.4794 | 1.00E-09 | 0.0017 | 759626 |
| rs262130 | 6 | T | C | 0.0127 | 0.1969 | 1.80E-08 | 0.0023 | 679542 |
| rs4495304 | 6 | C | T | -0.0194 | 0.067 | 5.00E-09 | 0.0033 | 774211 |
| rs2033529 | 6 | G | A | 0.0205 | 0.2936 | 1.90E-30 | 0.0018 | 792112 |
| rs3749897 | 6 | T | C | 0.0122 | 0.4172 | 8.40E-12 | 0.0018 | 638224 |
| rs1266874 | 6 | G | A | 0.014 | 0.3558 | 9.80E-15 | 0.0018 | 691020 |
| rs7761673 | 6 | A | T | -0.0126 | 0.2058 | 1.90E-09 | 0.0021 | 691716 |
| rs200810 | 6 | C | T | -0.0136 | 0.3716 | 5.50E-16 | 0.0017 | 793699 |
| rs9375702 | 6 | T | C | -0.0115 | 0.705 | 7.90E-10 | 0.0019 | 690564 |
| rs9362662 | 6 | G | A | -0.0112 | 0.5201 | 1.20E-10 | 0.0017 | 683953 |
| rs901630 | 6 | T | C | -0.0146 | 0.3973 | 1.90E-18 | 0.0017 | 794597 |
| rs17789218 | 6 | C | T | 0.013 | 0.2392 | 7.40E-12 | 0.0019 | 793904 |
| rs156201 | 6 | C | G | 0.0123 | 0.7606 | 5.80E-10 | 0.002 | 691835 |
| rs3800229 | 6 | T | G | 0.0175 | 0.7123 | 1.40E-22 | 0.0018 | 792474 |
| rs1538247 | 6 | C | T | 0.0108 | 0.3181 | 1.00E-08 | 0.0019 | 682726 |
| rs9478671 | 6 | G | A | 0.012 | 0.2087 | 1.70E-08 | 0.0021 | 688072 |
| rs486359 | 6 | C | G | 0.0112 | 0.4853 | 1.60E-11 | 0.0017 | 770999 |
| rs13191362 | 6 | G | A | -0.0236 | 0.1198 | 5.90E-21 | 0.0025 | 792699 |
| rs17207196 | 7 | T | C | -0.0221 | 0.4118 | 2.10E-35 | 0.0018 | 668894 |
| rs3807645 | 7 | A | G | -0.0166 | 0.221 | 2.40E-15 | 0.0021 | 683086 |
| rs11496125 | 7 | T | C | 0.0169 | 0.4212 | 3.00E-22 | 0.0017 | 684574 |
| rs10247983 | 7 | A | G | 0.0201 | 0.9213 | 1.70E-09 | 0.0033 | 672411 |
| rs3800637 | 7 | C | T | 0.0115 | 0.336 | 5.10E-10 | 0.0018 | 682001 |
| rs2907948 | 7 | A | G | -0.0141 | 0.2427 | 1.30E-13 | 0.0019 | 794299 |
| rs1830074 | 7 | C | T | 0.0115 | 0.288 | 1.40E-09 | 0.0019 | 689911 |
| rs4307239 | 7 | G | A | 0.0115 | 0.4578 | 3.90E-11 | 0.0017 | 687289 |
| rs12718572 | 7 | T | C | -0.0117 | 0.4024 | 3.00E-11 | 0.0018 | 686523 |
| rs38314 | 7 | A | G | -0.012 | 0.4912 | 4.70E-12 | 0.0017 | 689782 |
| rs13240600 | 7 | G | A | -0.0204 | 0.1552 | 3.50E-17 | 0.0024 | 692233 |
| rs7788008 | 7 | A | G | -0.0157 | 0.4445 | 1.10E-19 | 0.0017 | 690410 |
| rs6461115 | 7 | G | A | -0.0144 | 0.2285 | 1.20E-13 | 0.0019 | 791735 |
| rs4722398 | 7 | T | C | 0.0158 | 0.1336 | 3.60E-10 | 0.0025 | 692509 |
| rs10248136 | 7 | T | C | -0.0097 | 0.5142 | 2.00E-08 | 0.0017 | 686892 |
| rs202240867 | 7 | T | C | -0.0121 | 0.3422 | 3.30E-11 | 0.0018 | 692017 |
| rs11505821 | 7 | T | A | 0.0311 | 0.0601 | 2.70E-19 | 0.0035 | 758322 |
| rs7811342 | 7 | C | T | -0.0197 | 0.1058 | 1.10E-11 | 0.0029 | 676265 |
| rs10243319 | 7 | C | T | -0.0107 | 0.3939 | 1.20E-09 | 0.0018 | 690936 |
| rs774246 | 7 | G | A | 0.0153 | 0.1444 | 5.40E-10 | 0.0025 | 690818 |
| rs215634 | 7 | G | A | -0.0152 | 0.6212 | 2.60E-17 | 0.0018 | 681296 |
| rs10269783 | 7 | A | G | 0.0133 | 0.3896 | 1.40E-15 | 0.0017 | 790551 |
| rs7780752 | 7 | C | T | 0.0139 | 0.36 | 1.00E-14 | 0.0018 | 690830 |
| rs10953740 | 7 | G | A | -0.0153 | 0.5534 | 1.00E-18 | 0.0017 | 684419 |
| rs2283093 | 7 | T | C | 0.0127 | 0.2066 | 3.10E-09 | 0.0021 | 691773 |
| rs2543132 | 8 | C | G | 0.0146 | 0.8134 | 5.00E-11 | 0.0022 | 688326 |
| rs1421334 | 8 | C | A | -0.0125 | 0.5431 | 1.00E-12 | 0.0018 | 680665 |
| rs7826312 | 8 | C | T | 0.0104 | 0.5879 | 4.90E-10 | 0.0017 | 785343 |
| rs12546578 | 8 | A | T | 0.0146 | 0.7246 | 1.00E-13 | 0.002 | 689192 |
| rs12680842 | 8 | G | A | -0.0133 | 0.3205 | 4.40E-14 | 0.0018 | 782549 |
| rs12675063 | 8 | T | A | 0.0156 | 0.1131 | 1.30E-09 | 0.0026 | 789771 |
| rs4072917 | 8 | A | G | 0.0115 | 0.4694 | 6.90E-11 | 0.0018 | 684720 |
| rs7844647 | 8 | C | T | -0.0123 | 0.2681 | 2.80E-11 | 0.0018 | 793703 |
| rs13250058 | 8 | T | G | 0.0112 | 0.6771 | 2.90E-10 | 0.0018 | 787870 |
| rs6985109 | 8 | A | G | -0.0177 | 0.5338 | 1.50E-26 | 0.0017 | 793993 |
| rs17119937 | 8 | C | T | 0.0212 | 0.06905 | 5.60E-09 | 0.0036 | 668272 |
| rs6471941 | 8 | A | G | 0.0156 | 0.1684 | 3.10E-13 | 0.0021 | 793986 |
| rs12334877 | 8 | A | G | -0.0144 | 0.198 | 7.70E-11 | 0.0022 | 683820 |
| rs1431659 | 8 | G | A | -0.0196 | 0.7344 | 6.00E-24 | 0.0019 | 689739 |
| rs7819514 | 8 | A | G | -0.0107 | 0.3216 | 5.70E-09 | 0.0018 | 684955 |
| rs2694047 | 8 | G | A | 0.0188 | 0.747 | 3.90E-21 | 0.002 | 690295 |
| rs11781699 | 8 | C | T | 0.0132 | 0.1896 | 3.10E-10 | 0.0021 | 784642 |
| rs13263601 | 8 | C | A | 0.0154 | 0.3478 | 2.20E-17 | 0.0018 | 686196 |
| rs1982441 | 8 | T | G | 0.0175 | 0.1381 | 7.00E-12 | 0.0026 | 687705 |
| rs17405819 | 8 | C | T | -0.0215 | 0.301 | 4.30E-33 | 0.0018 | 795493 |
| rs7037266 | 9 | A | C | -0.0112 | 0.3739 | 3.50E-10 | 0.0018 | 691603 |
| rs1948080 | 9 | G | T | -0.0137 | 0.3749 | 1.10E-14 | 0.0018 | 690633 |
| rs10962550 | 9 | C | G | 0.0182 | 0.1801 | 6.20E-16 | 0.0022 | 690579 |
| rs10971709 | 9 | T | C | 0.0132 | 0.2062 | 6.20E-10 | 0.0021 | 688312 |
| rs3829849 | 9 | T | C | 0.0098 | 0.3589 | 5.90E-09 | 0.0017 | 793851 |
| rs10858334 | 9 | G | C | 0.0143 | 0.1415 | 2.70E-08 | 0.0026 | 672640 |
| rs1412235 | 9 | C | G | 0.0246 | 0.3175 | 6.00E-45 | 0.0017 | 790147 |
| rs1472169 | 9 | T | C | -0.0139 | 0.3772 | 2.80E-15 | 0.0018 | 689945 |
| rs7024334 | 9 | G | T | -0.0138 | 0.7742 | 3.10E-12 | 0.002 | 782431 |
| rs10984756 | 9 | G | C | 0.0174 | 0.1048 | 1.10E-09 | 0.0029 | 689917 |
| rs10968114 | 9 | C | A | -0.0113 | 0.4681 | 6.10E-11 | 0.0017 | 686025 |
| rs7025938 | 9 | G | C | 0.0166 | 0.3187 | 3.70E-19 | 0.0019 | 691581 |
| rs9408882 | 9 | A | G | -0.0093 | 0.4594 | 1.30E-08 | 0.0016 | 794283 |
| rs2174307 | 9 | C | G | 0.0121 | 0.4067 | 4.90E-12 | 0.0017 | 686559 |
| rs10867256 | 9 | T | C | -0.0118 | 0.553 | 8.70E-12 | 0.0017 | 689493 |
| rs13287131 | 9 | C | T | 0.0123 | 0.2491 | 6.80E-10 | 0.002 | 683808 |
| rs7869771 | 9 | C | A | -0.014 | 0.2647 | 4.90E-13 | 0.0019 | 679436 |
| rs9650755 | 9 | G | A | 0.0154 | 0.2664 | 2.80E-15 | 0.002 | 691183 |
| rs380857 | 9 | A | C | -0.0151 | 0.8878 | 3.60E-08 | 0.0027 | 691507 |
| rs1535660 | 9 | C | T | -0.0147 | 0.8554 | 5.20E-09 | 0.0025 | 690624 |
| rs4740619 | 9 | C | T | -0.0186 | 0.4521 | 2.30E-30 | 0.0016 | 794491 |
| rs10811871 | 9 | G | A | -0.0108 | 0.3829 | 1.60E-09 | 0.0018 | 686376 |
| rs1187352 | 9 | C | T | 0.0119 | 0.6518 | 6.00E-11 | 0.0018 | 688522 |
| rs1928295 | 9 | C | T | -0.0141 | 0.4461 | 5.40E-18 | 0.0016 | 793649 |
| rs7871866 | 9 | C | G | 0.0187 | 0.1531 | 2.30E-14 | 0.0024 | 683494 |
| rs12098284 | 10 | T | C | 0.0178 | 0.1241 | 1.80E-11 | 0.0026 | 686167 |
| rs11251352 | 10 | G | A | 0.0109 | 0.5988 | 7.00E-10 | 0.0018 | 690804 |
| rs12779328 | 10 | T | C | 0.0105 | 0.2833 | 4.50E-08 | 0.0019 | 690736 |
| rs10795422 | 10 | G | A | 0.0139 | 0.6905 | 9.30E-14 | 0.0019 | 692108 |
| rs1624134 | 10 | C | G | 0.0101 | 0.4068 | 1.10E-08 | 0.0018 | 690572 |
| rs2163188 | 10 | C | G | 0.0131 | 0.474 | 2.00E-14 | 0.0017 | 686502 |
| rs7899106 | 10 | G | A | 0.0331 | 0.04777 | 1.00E-18 | 0.0037 | 793689 |
| rs7903146 | 10 | T | C | -0.0181 | 0.2912 | 1.30E-23 | 0.0018 | 795624 |
| rs7084454 | 10 | A | G | 0.0193 | 0.335 | 4.00E-25 | 0.0019 | 678564 |
| rs12762034 | 10 | C | T | 0.024 | 0.07583 | 7.30E-14 | 0.0032 | 692191 |
| rs3977755 | 10 | T | C | -0.0135 | 0.2804 | 5.90E-13 | 0.0019 | 731529 |
| rs3904244 | 10 | A | T | 0.0155 | 0.1377 | 4.30E-10 | 0.0025 | 691180 |
| rs1937683 | 10 | T | C | 0.0109 | 0.6699 | 3.20E-09 | 0.0018 | 692539 |
| rs17113297 | 10 | T | C | 0.0166 | 0.2082 | 2.10E-15 | 0.0021 | 686357 |
| rs17636031 | 10 | C | T | 0.016 | 0.2701 | 1.20E-17 | 0.0019 | 782807 |
| rs999889 | 10 | A | G | -0.0108 | 0.2818 | 1.40E-08 | 0.0019 | 690572 |
| rs10887578 | 10 | C | G | 0.0128 | 0.4896 | 1.60E-13 | 0.0017 | 679172 |
| rs577525 | 10 | C | T | 0.0166 | 0.5676 | 9.70E-22 | 0.0017 | 690616 |
| rs1681740 | 10 | C | A | -0.0115 | 0.3933 | 1.10E-10 | 0.0018 | 673459 |
| rs845084 | 10 | A | G | 0.014 | 0.2678 | 1.30E-12 | 0.002 | 685413 |
| rs4880341 | 10 | T | C | -0.0118 | 0.5606 | 1.10E-11 | 0.0017 | 689012 |
| rs4757144 | 11 | A | G | 0.0169 | 0.5878 | 5.60E-22 | 0.0018 | 690082 |
| rs6265 | 11 | T | C | -0.0412 | 0.1951 | 1.00E-86 | 0.0021 | 795458 |
| rs7102454 | 11 | C | T | 0.0158 | 0.3435 | 2.40E-18 | 0.0018 | 691134 |
| rs592483 | 11 | T | C | -0.0147 | 0.5716 | 2.00E-18 | 0.0017 | 781871 |
| rs349088 | 11 | A | C | -0.0128 | 0.4976 | 1.80E-13 | 0.0017 | 684401 |
| rs7358465 | 11 | T | C | 0.0103 | 0.6781 | 3.00E-08 | 0.0019 | 686935 |
| rs2605603 | 11 | A | G | -0.0103 | 0.4887 | 2.50E-10 | 0.0016 | 790857 |
| rs10750215 | 11 | T | G | 0.0108 | 0.3883 | 1.30E-10 | 0.0017 | 788895 |
| rs4936175 | 11 | C | T | 0.0122 | 0.4445 | 1.40E-12 | 0.0017 | 692569 |
| rs12416812 | 11 | A | G | 0.0111 | 0.5088 | 6.10E-12 | 0.0016 | 793338 |
| rs10832778 | 11 | G | C | 0.0125 | 0.6222 | 1.30E-13 | 0.0017 | 783042 |
| rs2065418 | 11 | G | T | -0.0166 | 0.3623 | 3.60E-20 | 0.0018 | 691707 |
| rs10768994 | 11 | C | T | -0.0114 | 0.4337 | 6.40E-12 | 0.0017 | 791685 |
| rs6591407 | 11 | A | C | -0.0118 | 0.1861 | 1.90E-08 | 0.0021 | 794246 |
| rs4937870 | 11 | G | A | -0.0109 | 0.3172 | 8.80E-09 | 0.0019 | 683154 |
| rs7925214 | 11 | T | C | 0.0147 | 0.5133 | 4.40E-17 | 0.0018 | 677603 |
| rs491711 | 11 | C | A | -0.0115 | 0.316 | 1.10E-09 | 0.0019 | 685113 |
| rs10742752 | 11 | C | T | 0.0124 | 0.6159 | 1.10E-13 | 0.0017 | 792704 |
| rs7117238 | 11 | A | G | -0.0131 | 0.168 | 2.50E-09 | 0.0022 | 788879 |
| rs4310573 | 11 | T | C | 0.0116 | 0.7813 | 3.50E-08 | 0.0021 | 682567 |
| rs1048932 | 11 | A | C | -0.016 | 0.4162 | 3.80E-22 | 0.0017 | 795167 |
| rs4929923 | 11 | C | T | 0.0181 | 0.6376 | 7.20E-27 | 0.0017 | 794933 |
| rs4237643 | 11 | G | T | -0.0223 | 0.6938 | 4.30E-33 | 0.0019 | 692491 |
| rs685870 | 11 | C | T | 0.012 | 0.7035 | 2.40E-10 | 0.0019 | 688423 |
| rs11030618 | 11 | T | C | 0.011 | 0.5679 | 2.40E-10 | 0.0017 | 690005 |
| rs7124681 | 11 | A | C | 0.0263 | 0.4133 | 3.20E-58 | 0.0016 | 795474 |
| rs1465900 | 11 | C | A | -0.0125 | 0.2188 | 4.80E-10 | 0.002 | 779748 |
| rs1784460 | 11 | A | T | 0.0132 | 0.4035 | 9.00E-14 | 0.0018 | 680042 |
| rs12364470 | 11 | G | T | 0.0178 | 0.1626 | 1.10E-15 | 0.0022 | 787411 |
| rs2429150 | 12 | C | A | 0.0111 | 0.4164 | 2.70E-10 | 0.0018 | 686276 |
| rs7970953 | 12 | A | G | 0.0135 | 0.29 | 9.80E-14 | 0.0018 | 788417 |
| rs10878946 | 12 | T | C | -0.0141 | 0.714 | 3.60E-13 | 0.0019 | 685707 |
| rs11066188 | 12 | A | G | -0.012 | 0.4181 | 8.10E-13 | 0.0017 | 792755 |
| rs11615578 | 12 | T | C | 0.013 | 0.2474 | 8.10E-11 | 0.002 | 669422 |
| rs11170468 | 12 | C | A | -0.0123 | 0.2326 | 1.90E-10 | 0.0019 | 795265 |
| rs7138803 | 12 | A | G | 0.03 | 0.3772 | 2.30E-71 | 0.0017 | 795588 |
| rs705704 | 12 | A | G | -0.0131 | 0.3304 | 1.90E-13 | 0.0018 | 743597 |
| rs11105839 | 12 | A | T | -0.0109 | 0.3799 | 1.10E-10 | 0.0017 | 781573 |
| rs7488867 | 12 | T | C | -0.0204 | 0.2639 | 8.40E-24 | 0.002 | 635746 |
| rs10492229 | 12 | T | C | 0.0142 | 0.2268 | 7.70E-14 | 0.0019 | 794845 |
| rs4148866 | 12 | T | C | 0.0098 | 0.4068 | 4.00E-08 | 0.0018 | 676418 |
| rs2306537 | 12 | G | A | 0.0133 | 0.3092 | 8.70E-13 | 0.0019 | 691923 |
| rs12422552 | 12 | C | G | -0.0134 | 0.2663 | 1.60E-11 | 0.002 | 689543 |
| rs2608703 | 12 | A | C | 0.0142 | 0.4546 | 1.90E-16 | 0.0017 | 686700 |
| rs12299814 | 12 | A | C | -0.0157 | 0.2525 | 5.20E-15 | 0.002 | 687962 |
| rs2943465 | 12 | C | T | 0.0248 | 0.9444 | 2.00E-10 | 0.0039 | 686547 |
| rs11173522 | 12 | A | C | 0.0128 | 0.2078 | 1.10E-09 | 0.0021 | 691593 |
| rs11115176 | 12 | C | T | -0.0121 | 0.2399 | 2.00E-10 | 0.0019 | 792384 |
| rs4842491 | 12 | T | C | 0.0098 | 0.7138 | 4.00E-08 | 0.0018 | 795312 |
| rs11609659 | 12 | C | T | -0.0154 | 0.2371 | 2.20E-14 | 0.002 | 679177 |
| rs12369179 | 12 | T | C | -0.0359 | 0.08782 | 2.50E-31 | 0.0031 | 674260 |
| rs11611246 | 12 | T | G | 0.024 | 0.21 | 5.00E-32 | 0.002 | 779823 |
| rs1843328 | 12 | A | C | -0.0099 | 0.5085 | 7.90E-09 | 0.0017 | 686814 |
| rs7318817 | 13 | T | C | -0.0155 | 0.6071 | 2.70E-18 | 0.0018 | 691917 |
| rs1927790 | 13 | C | T | 0.0148 | 0.4109 | 1.80E-19 | 0.0016 | 794326 |
| rs9522285 | 13 | A | G | 0.0127 | 0.4143 | 2.50E-13 | 0.0017 | 690681 |
| rs6561943 | 13 | T | C | 0.0119 | 0.2595 | 4.20E-10 | 0.0019 | 793951 |
| rs9300422 | 13 | G | A | -0.0103 | 0.6903 | 4.00E-09 | 0.0018 | 795011 |
| rs7334078 | 13 | C | T | -0.0121 | 0.2882 | 2.20E-10 | 0.0019 | 688374 |
| rs1218822 | 13 | A | G | 0.0168 | 0.6663 | 1.90E-22 | 0.0017 | 794711 |
| rs7983065 | 13 | T | C | -0.0148 | 0.4503 | 8.90E-18 | 0.0017 | 690924 |
| rs12429545 | 13 | A | G | 0.0316 | 0.1248 | 9.60E-38 | 0.0025 | 778918 |
| rs8181823 | 13 | C | A | 0.0127 | 0.7614 | 4.10E-10 | 0.002 | 691345 |
| rs9547153 | 13 | G | A | 0.0098 | 0.3839 | 8.70E-09 | 0.0017 | 775400 |
| rs1112613 | 13 | A | G | -0.0133 | 0.1762 | 3.40E-09 | 0.0023 | 682816 |
| rs9538162 | 13 | C | T | -0.0156 | 0.4138 | 4.80E-19 | 0.0018 | 690345 |
| rs1144387 | 13 | C | G | 0.0098 | 0.5714 | 1.60E-08 | 0.0017 | 687565 |
| rs7998796 | 13 | G | A | 0.0105 | 0.3373 | 1.10E-08 | 0.0018 | 689430 |
| rs2479958 | 13 | G | A | -0.0154 | 0.5075 | 1.50E-17 | 0.0018 | 664268 |
| rs17446257 | 13 | A | G | 0.0153 | 0.1292 | 2.90E-09 | 0.0026 | 690630 |
| rs9571687 | 13 | A | C | -0.0129 | 0.329 | 2.80E-12 | 0.0018 | 690974 |
| rs1330052 | 13 | G | C | 0.0132 | 0.3504 | 1.50E-13 | 0.0018 | 691613 |
| rs3007105 | 14 | T | C | 0.0142 | 0.4697 | 1.10E-17 | 0.0017 | 785488 |
| rs4981693 | 14 | A | G | 0.0206 | 0.771 | 6.90E-24 | 0.002 | 689120 |
| rs226000 | 14 | T | C | 0.0119 | 0.8245 | 3.60E-08 | 0.0022 | 793480 |
| rs3902951 | 14 | G | T | 0.0134 | 0.2455 | 7.00E-12 | 0.002 | 773819 |
| rs1285997 | 14 | G | C | 0.0142 | 0.7153 | 1.20E-13 | 0.0019 | 684235 |
| rs9989141 | 14 | T | C | 0.0162 | 0.6387 | 3.60E-21 | 0.0017 | 752768 |
| rs709400 | 14 | G | A | -0.015 | 0.3818 | 4.60E-19 | 0.0017 | 795379 |
| rs7148846 | 14 | G | T | 0.0124 | 0.1896 | 2.20E-08 | 0.0022 | 687940 |
| rs4430672 | 14 | C | T | -0.0127 | 0.8004 | 3.90E-09 | 0.0022 | 691400 |
| rs217671 | 14 | G | A | 0.0144 | 0.2719 | 1.30E-13 | 0.0019 | 691456 |
| rs768840 | 14 | A | G | 0.0114 | 0.4183 | 2.00E-10 | 0.0018 | 677485 |
| rs7144011 | 14 | T | G | 0.0282 | 0.2136 | 5.20E-47 | 0.002 | 794117 |
| rs12888545 | 14 | G | A | 0.0136 | 0.2519 | 9.10E-12 | 0.002 | 688605 |
| rs10132280 | 14 | A | C | -0.0223 | 0.3017 | 5.60E-35 | 0.0018 | 786578 |
| rs872281 | 14 | T | C | -0.0151 | 0.1728 | 4.70E-11 | 0.0023 | 685310 |
| rs12888955 | 14 | A | G | -0.0178 | 0.6513 | 1.40E-22 | 0.0018 | 691849 |
| rs1836303 | 15 | G | A | 0.0116 | 0.3873 | 5.30E-11 | 0.0018 | 688991 |
| rs3736485 | 15 | G | A | -0.0134 | 0.5443 | 2.50E-16 | 0.0016 | 790404 |
| rs339991 | 15 | G | A | 0.0124 | 0.5631 | 1.20E-12 | 0.0018 | 686476 |
| rs12593036 | 15 | G | A | -0.0154 | 0.2993 | 3.80E-16 | 0.0019 | 686055 |
| rs4906908 | 15 | G | T | 0.0103 | 0.5253 | 2.50E-09 | 0.0017 | 691345 |
| rs8036040 | 15 | A | C | 0.0109 | 0.4932 | 2.70E-10 | 0.0017 | 691068 |
| rs10518694 | 15 | A | C | 0.0146 | 0.1424 | 3.30E-09 | 0.0025 | 690554 |
| rs17238110 | 15 | G | A | -0.0353 | 0.1634 | 2.00E-12 | 0.005 | 775505 |
| rs16953563 | 15 | A | G | -0.0134 | 0.2516 | 1.50E-11 | 0.002 | 691761 |
| rs11855853 | 15 | T | C | -0.0145 | 0.2649 | 2.40E-13 | 0.002 | 682564 |
| rs7172627 | 15 | G | A | 0.0117 | 0.4719 | 1.10E-11 | 0.0017 | 690458 |
| rs17311369 | 15 | T | C | -0.0104 | 0.3278 | 3.10E-08 | 0.0019 | 673102 |
| rs13329567 | 15 | T | C | -0.0293 | 0.2308 | 1.00E-50 | 0.002 | 793953 |
| rs7181498 | 15 | C | T | -0.0163 | 0.6309 | 1.00E-19 | 0.0018 | 690980 |
| rs12905439 | 15 | G | C | -0.0118 | 0.3393 | 1.40E-10 | 0.0018 | 675205 |
| rs9806742 | 15 | A | G | 0.0208 | 0.8826 | 1.40E-15 | 0.0026 | 692509 |
| rs12914489 | 15 | A | G | 0.0165 | 0.1103 | 3.80E-10 | 0.0026 | 795244 |
| rs8027205 | 15 | G | C | -0.0108 | 0.3967 | 1.40E-09 | 0.0018 | 685725 |
| rs12448257 | 16 | A | G | 0.0184 | 0.218 | 8.10E-20 | 0.002 | 779628 |
| rs879620 | 16 | T | C | 0.0231 | 0.6179 | 5.30E-38 | 0.0018 | 688377 |
| rs9926784 | 16 | C | T | -0.0258 | 0.1822 | 9.90E-35 | 0.0021 | 789617 |
| rs9927848 | 16 | A | C | -0.0122 | 0.7326 | 6.40E-10 | 0.002 | 687060 |
| rs1477199 | 16 | G | A | 0.0228 | 0.1451 | 9.40E-22 | 0.0024 | 794442 |
| rs825688 | 16 | T | C | -0.0095 | 0.456 | 4.70E-08 | 0.0017 | 686144 |
| rs2361988 | 16 | C | T | -0.0155 | 0.2539 | 5.20E-15 | 0.002 | 690251 |
| rs1896767 | 16 | A | G | -0.0109 | 0.5376 | 2.40E-10 | 0.0017 | 686262 |
| rs4786903 | 16 | G | A | 0.0125 | 0.7368 | 3.50E-10 | 0.002 | 680139 |
| rs7196720 | 16 | C | T | -0.0129 | 0.5068 | 7.30E-14 | 0.0017 | 689863 |
| rs3814883 | 16 | T | C | 0.0232 | 0.4764 | 1.10E-40 | 0.0017 | 685519 |
| rs8047395 | 16 | A | G | 0.0642 | 0.5061 | 1.00E-200 | 0.0017 | 788856 |
| rs12922346 | 16 | C | G | 0.0136 | 0.2657 | 1.00E-11 | 0.002 | 679615 |
| rs4783830 | 16 | A | G | -0.0105 | 0.3074 | 2.40E-08 | 0.0019 | 675527 |
| rs889398 | 16 | T | C | -0.0196 | 0.4247 | 1.30E-32 | 0.0016 | 789694 |
| rs12933482 | 16 | G | A | 0.0186 | 0.1048 | 4.90E-11 | 0.0028 | 691477 |
| rs7206608 | 16 | G | C | 0.0132 | 0.3146 | 1.30E-12 | 0.0019 | 689058 |
| rs7498665 | 16 | G | A | 0.0271 | 0.4038 | 5.60E-60 | 0.0017 | 790299 |
| rs6500208 | 16 | A | G | 0.014 | 0.2006 | 4.10E-12 | 0.002 | 781931 |
| rs756717 | 16 | A | G | -0.0148 | 0.3973 | 5.40E-18 | 0.0017 | 771976 |
| rs4516268 | 17 | A | C | -0.0217 | 0.1925 | 5.20E-25 | 0.0021 | 786617 |
| rs4986044 | 17 | T | C | -0.0164 | 0.4687 | 3.30E-23 | 0.0016 | 787219 |
| rs208015 | 17 | C | T | -0.0356 | 0.9216 | 1.40E-25 | 0.0034 | 691575 |
| rs4968656 | 17 | G | A | 0.0116 | 0.3216 | 8.20E-10 | 0.0019 | 675153 |
| rs12939549 | 17 | G | A | -0.018 | 0.4335 | 2.70E-28 | 0.0016 | 793950 |
| rs12936083 | 17 | G | A | 0.0139 | 0.3267 | 4.10E-13 | 0.0019 | 633615 |
| rs12150665 | 17 | C | T | -0.0162 | 0.4058 | 1.60E-22 | 0.0017 | 795501 |
| rs2411182 | 17 | A | G | 0.0123 | 0.6943 | 7.70E-11 | 0.0019 | 691730 |
| rs962273 | 17 | C | T | 0.0137 | 0.7057 | 2.60E-13 | 0.0019 | 692594 |
| rs3935648 | 17 | G | C | -0.0125 | 0.2328 | 6.80E-09 | 0.0022 | 629303 |
| rs1075901 | 17 | C | T | 0.0121 | 0.5639 | 1.20E-13 | 0.0016 | 794789 |
| rs7222349 | 17 | A | G | 0.0115 | 0.3441 | 3.30E-10 | 0.0018 | 692215 |
| rs391300 | 17 | C | T | -0.0119 | 0.6275 | 3.10E-12 | 0.0017 | 791120 |
| rs11656076 | 17 | A | G | -0.0142 | 0.2254 | 5.60E-12 | 0.0021 | 691283 |
| rs8071182 | 17 | A | G | 0.0133 | 0.1735 | 2.10E-09 | 0.0022 | 771437 |
| rs12602912 | 17 | T | C | 0.0176 | 0.2048 | 9.90E-18 | 0.0021 | 777510 |
| rs8097672 | 18 | T | A | 0.02 | 0.1528 | 8.40E-16 | 0.0025 | 686063 |
| rs1430387 | 18 | C | T | -0.0114 | 0.4295 | 5.80E-11 | 0.0017 | 689325 |
| rs9783858 | 18 | T | C | 0.0091 | 0.5191 | 3.30E-08 | 0.0017 | 770874 |
| rs8097783 | 18 | A | G | -0.0389 | 0.07554 | 7.20E-36 | 0.0031 | 795408 |
| rs17710386 | 18 | C | T | 0.0126 | 0.3319 | 1.00E-12 | 0.0018 | 783810 |
| rs11150911 | 18 | C | A | -0.0133 | 0.7191 | 4.70E-13 | 0.0018 | 781716 |
| rs4800191 | 18 | C | G | 0.0103 | 0.6369 | 2.50E-09 | 0.0017 | 785353 |
| rs1365466 | 18 | T | C | -0.0137 | 0.7406 | 3.30E-13 | 0.0019 | 791868 |
| rs559231 | 18 | T | G | 0.0135 | 0.3956 | 2.40E-14 | 0.0018 | 685154 |
| rs663129 | 18 | A | G | 0.0545 | 0.2301 | 1.60E-178 | 0.0019 | 788948 |
| rs1241986 | 18 | A | G | -0.0139 | 0.8479 | 1.10E-08 | 0.0024 | 687757 |
| rs7239575 | 18 | C | T | -0.0202 | 0.4832 | 7.40E-32 | 0.0017 | 692313 |
| rs1158805 | 18 | A | C | -0.0137 | 0.3766 | 1.20E-14 | 0.0018 | 691776 |
| rs8090983 | 18 | G | A | 0.0118 | 0.3314 | 2.00E-10 | 0.0018 | 682470 |
| rs9951619 | 18 | G | T | 0.0156 | 0.7643 | 1.40E-15 | 0.002 | 772643 |
| rs287104 | 19 | A | G | 0.0115 | 0.6604 | 4.40E-11 | 0.0017 | 787307 |
| rs895330 | 19 | G | C | -0.0201 | 0.1924 | 5.50E-19 | 0.0023 | 684271 |
| rs273504 | 19 | G | A | 0.0153 | 0.4266 | 4.40E-18 | 0.0018 | 690672 |
| rs17724992 | 19 | G | A | -0.0183 | 0.2596 | 1.00E-22 | 0.0019 | 785851 |
| rs11880870 | 19 | G | A | -0.0189 | 0.4801 | 1.00E-28 | 0.0017 | 717350 |
| rs1982725 | 19 | T | C | 0.0097 | 0.4778 | 3.30E-08 | 0.0017 | 683155 |
| rs11084553 | 19 | G | A | -0.021 | 0.1518 | 1.80E-18 | 0.0024 | 691103 |
| rs11672660 | 19 | T | C | -0.034 | 0.2049 | 1.70E-60 | 0.0021 | 768426 |
| rs9304665 | 19 | A | T | 0.0229 | 0.7633 | 2.90E-29 | 0.002 | 689470 |
| rs10408324 | 19 | T | C | -0.0124 | 0.2744 | 9.50E-11 | 0.0019 | 690737 |
| rs12981256 | 19 | A | G | 0.0142 | 0.5325 | 1.10E-15 | 0.0018 | 678327 |
| rs998732 | 19 | G | A | -0.0171 | 0.1578 | 2.00E-14 | 0.0022 | 793852 |
| rs17513613 | 19 | C | T | 0.0186 | 0.3236 | 3.60E-26 | 0.0018 | 789575 |
| rs769449 | 19 | A | G | -0.0254 | 0.1161 | 2.30E-20 | 0.0027 | 566857 |
| rs6050446 | 20 | G | A | 0.0343 | 0.97001 | 4.40E-13 | 0.0047 | 766287 |
| rs11908637 | 20 | A | G | -0.012 | 0.236 | 4.90E-09 | 0.0021 | 691443 |
| rs6011457 | 20 | A | T | -0.0116 | 0.4975 | 2.70E-11 | 0.0017 | 690692 |
| rs4813619 | 20 | T | G | -0.0108 | 0.5101 | 2.30E-09 | 0.0018 | 622760 |
| rs1321432 | 20 | C | A | 0.0201 | 0.6321 | 3.50E-29 | 0.0018 | 686481 |
| rs4012234 | 20 | G | T | 0.0141 | 0.5924 | 9.90E-16 | 0.0018 | 689653 |
| rs2143253 | 20 | A | G | -0.0188 | 0.1189 | 1.10E-12 | 0.0026 | 684760 |
| rs2425840 | 20 | C | A | 0.0119 | 0.4059 | 1.60E-11 | 0.0018 | 681680 |
| rs8123881 | 20 | G | A | 0.0196 | 0.1299 | 4.40E-16 | 0.0024 | 793018 |
| rs852056 | 20 | C | T | -0.0128 | 0.7584 | 1.80E-10 | 0.002 | 691874 |
| rs1884389 | 20 | T | C | -0.0103 | 0.4289 | 4.00E-09 | 0.0017 | 683669 |
| rs2423668 | 20 | C | T | -0.0105 | 0.5505 | 2.80E-08 | 0.0019 | 527352 |
| rs1409818 | 20 | T | C | 0.0201 | 0.1156 | 2.50E-12 | 0.0029 | 690984 |
| rs17806379 | 20 | T | C | -0.0258 | 0.1789 | 1.50E-30 | 0.0022 | 690043 |
| rs6512302 | 20 | C | G | 0.0142 | 0.7511 | 2.10E-12 | 0.002 | 686053 |
| rs427943 | 21 | C | A | 0.017 | 0.5669 | 7.30E-23 | 0.0017 | 712095 |
| rs2832283 | 21 | A | G | 0.0115 | 0.2208 | 5.80E-09 | 0.002 | 792324 |
| rs13047416 | 21 | G | C | -0.0154 | 0.3769 | 2.20E-17 | 0.0018 | 683228 |
| rs2836964 | 21 | C | T | -0.011 | 0.3576 | 1.30E-09 | 0.0018 | 692353 |
| rs4818225 | 21 | G | A | 0.0117 | 0.6606 | 2.30E-10 | 0.0018 | 688274 |
| rs175165 | 22 | G | T | -0.0103 | 0.3941 | 5.20E-09 | 0.0018 | 690545 |
| rs9615905 | 22 | T | C | 0.011 | 0.45 | 2.70E-10 | 0.0017 | 690274 |
| rs11538 | 22 | G | A | 0.0135 | 0.1805 | 3.30E-09 | 0.0023 | 692349 |
| rs6712 | 22 | C | G | 0.0138 | 0.1368 | 4.40E-08 | 0.0025 | 688469 |
| rs138289 | 22 | T | A | -0.0103 | 0.4829 | 3.30E-09 | 0.0017 | 687258 |
| rs2285178 | 22 | C | T | 0.0112 | 0.3109 | 9.40E-09 | 0.0019 | 638268 |
| rs4820408 | 22 | G | T | -0.0151 | 0.592 | 2.10E-19 | 0.0017 | 794185 |
| 337 SNPs are associated with waist circumference | | | | | | | | |
| SNP | chr.exposure | effect_allele.exposure | effect_allele.exposure | effect_allele.exposure | effect_allele.exposure | effect_allele.exposure | effect_allele.exposure | effect_allele.exposure |
| rs6695898 | 1 | A | G | -0.0117009 | NA | 3.77459E-10 | 0.00186824 | 407661 |
| rs6659830 | 1 | A | G | -0.0176069 | NA | 9.50824E-19 | 0.00199157 | 407661 |
| rs146322930 | 1 | TTC | T | -0.0203555 | NA | 2.62301E-19 | 0.00226588 | 407661 |
| rs41279738 | 1 | G | T | 0.0491105 | NA | 5.77963E-17 | 0.0058677 | 407661 |
| rs115056380 | 1 | A | G | -0.0243843 | NA | 1.82037E-08 | 0.00433247 | 407661 |
| rs543874 | 1 | G | A | 0.035504 | NA | 4.03367E-54 | 0.00229202 | 407661 |
| rs12731208 | 1 | G | T | 0.0113729 | NA | 5.38952E-09 | 0.00194919 | 407661 |
| rs1198443 | 1 | T | A | -0.0174437 | NA | 8.41201E-11 | 0.00268652 | 407661 |
| rs112566467 | 1 | T | C | 0.0188265 | NA | 1.82936E-16 | 0.00228678 | 407661 |
| rs200090476 | 1 | A | G | 0.0138767 | NA | 9.27897E-13 | 0.0019433 | 407661 |
| rs156902 | 1 | T | G | -0.0156652 | NA | 6.66362E-10 | 0.00253733 | 407661 |
| rs588660 | 1 | A | G | 0.0148905 | NA | 3.28473E-15 | 0.00188976 | 407661 |
| rs4844809 | 1 | C | G | 0.0197821 | NA | 6.07855E-13 | 0.00274801 | 407661 |
| rs12044012 | 1 | A | G | -0.0122505 | NA | 7.76104E-10 | 0.00199204 | 407661 |
| rs34517439 | 1 | A | C | 0.0302012 | NA | 3.39E-26 | 0.00285242 | 407661 |
| rs12072739 | 1 | G | A | 0.0174869 | NA | 4.64943E-15 | 0.00223159 | 407661 |
| rs2618039 | 1 | T | A | 0.013003 | NA | 1.17761E-11 | 0.00191702 | 407661 |
| rs61813324 | 1 | T | C | 0.0217781 | NA | 2.6687E-15 | 0.00275481 | 407661 |
| rs1490382 | 1 | G | A | -0.0111753 | NA | 5.65731E-09 | 0.00191798 | 407661 |
| rs12726165 | 1 | T | C | 0.0115314 | NA | 2.02456E-09 | 0.00192324 | 407661 |
| rs12030345 | 1 | A | G | -0.0190189 | NA | 3.63279E-08 | 0.00345306 | 407661 |
| rs12140153 | 1 | T | G | -0.0279147 | NA | 5.71347E-18 | 0.00323157 | 407661 |
| rs7519259 | 1 | A | G | 0.0134443 | NA | 7.10886E-13 | 0.00187317 | 407661 |
| rs6604866 | 1 | C | G | -0.0110683 | NA | 3.68358E-09 | 0.00187667 | 407661 |
| rs12088070 | 1 | T | C | 0.011437 | NA | 1.63629E-08 | 0.00202545 | 407661 |
| rs61826867 | 1 | G | A | 0.017592 | NA | 3.35166E-09 | 0.00297496 | 407661 |
| rs815163 | 1 | C | T | -0.0142686 | NA | 2.56921E-14 | 0.00187293 | 407661 |
| rs2678204 | 1 | G | T | 0.0166782 | NA | 2.03845E-17 | 0.00196409 | 407661 |
| rs77165542 | 2 | T | C | -0.0721526 | NA | 1.20559E-45 | 0.00508807 | 407661 |
| rs10182458 | 2 | G | A | 0.0198069 | NA | 1.99434E-26 | 0.00186199 | 407661 |
| rs6739755 | 2 | G | A | -0.0155629 | NA | 3.20406E-16 | 0.00190596 | 407661 |
| rs7583157 | 2 | G | A | 0.0130497 | NA | 2.36233E-10 | 0.0020597 | 407661 |
| rs1048013 | 2 | T | C | 0.0110185 | NA | 3.97375E-09 | 0.0018722 | 407661 |
| rs284532 | 2 | G | T | -0.0107684 | NA | 7.76998E-09 | 0.00186519 | 407661 |
| rs113784412 | 2 | T | C | 0.0144613 | NA | 7.46449E-13 | 0.00201673 | 407661 |
| rs2861692 | 2 | C | T | -0.0166676 | NA | 1.23339E-15 | 0.00208318 | 407661 |
| rs4851283 | 2 | G | C | -0.0172043 | NA | 1.43847E-17 | 0.00201645 | 407661 |
| rs1609303 | 2 | A | T | 0.0158413 | NA | 2.88071E-16 | 0.00193702 | 407661 |
| rs35882248 | 2 | T | C | 0.0164641 | NA | 2.25944E-16 | 0.002006 | 407661 |
| rs4671328 | 2 | G | T | -0.0156187 | NA | 1.22067E-16 | 0.00188607 | 407661 |
| rs308911 | 2 | G | A | -0.0118731 | NA | 8.60617E-09 | 0.00206269 | 407661 |
| rs34847111 | 2 | T | C | 0.0124083 | NA | 8.27713E-09 | 0.0021532 | 407661 |
| rs114964326 | 2 | A | G | -0.0339599 | NA | 8.82206E-10 | 0.0055405 | 407661 |
| rs6751993 | 2 | G | A | 0.0428629 | NA | 9.30679E-66 | 0.00250263 | 407661 |
| rs935166 | 2 | A | G | -0.014013 | NA | 5.29663E-14 | 0.00186235 | 407661 |
| rs3770821 | 2 | C | T | 0.0130904 | NA | 4.67089E-11 | 0.00198911 | 407661 |
| rs6545144 | 2 | A | T | -0.0180689 | NA | 3.3986E-13 | 0.00248281 | 407661 |
| rs12616638 | 2 | G | A | -0.0104379 | NA | 4.0412E-08 | 0.00190159 | 407661 |
| rs72820274 | 2 | A | G | 0.0118657 | NA | 3.46027E-10 | 0.00189047 | 407661 |
| rs778094 | 2 | A | G | -0.0114182 | NA | 1.43031E-09 | 0.00188666 | 407661 |
| rs4482463 | 2 | A | C | -0.0277234 | NA | 3.6358E-15 | 0.00352406 | 407661 |
| rs73985439 | 2 | C | A | 0.0132309 | NA | 5.7161E-11 | 0.00201968 | 407661 |
| rs1405261 | 2 | A | T | -0.0107553 | NA | 1.13355E-08 | 0.00188377 | 407661 |
| rs754209 | 2 | T | C | 0.0111077 | NA | 9.1622E-09 | 0.00193327 | 407661 |
| rs6433243 | 2 | C | T | -0.0132939 | NA | 9.33469E-12 | 0.00195028 | 407661 |
| rs34234296 | 2 | A | G | -0.0129962 | NA | 1.51043E-11 | 0.00192625 | 407661 |
| rs13427822 | 2 | G | A | -0.0149809 | NA | 1.35831E-12 | 0.00211349 | 407661 |
| rs183181489 | 3 | C | T | 0.0147892 | NA | 1.88973E-11 | 0.00220264 | 407661 |
| rs9843653 | 3 | C | T | 0.0191257 | NA | 9.22147E-25 | 0.00186155 | 407661 |
| rs9842991 | 3 | A | G | 0.0110603 | NA | 2.46405E-08 | 0.00198363 | 407661 |
| rs9835772 | 3 | T | A | 0.0157517 | NA | 3.66606E-13 | 0.00216746 | 407661 |
| rs1436348 | 3 | G | A | 0.0122897 | NA | 7.03234E-11 | 0.00188493 | 407661 |
| rs1320903 | 3 | A | G | 0.0187779 | NA | 5.46764E-21 | 0.0019977 | 407661 |
| rs701146 | 3 | A | T | 0.0109782 | NA | 3.93632E-09 | 0.00186487 | 407661 |
| rs869400 | 3 | G | T | 0.0208153 | NA | 4.67305E-18 | 0.00240331 | 407661 |
| rs9343 | 3 | G | A | 0.0103346 | NA | 2.77198E-08 | 0.00186032 | 407661 |
| rs2569993 | 3 | C | T | 0.0134039 | NA | 2.06158E-11 | 0.0020001 | 407661 |
| rs9867068 | 3 | G | C | 0.0168157 | NA | 6.44911E-15 | 0.00215728 | 407661 |
| rs9968060 | 3 | T | C | 0.0146596 | NA | 1.24796E-13 | 0.00197793 | 407661 |
| rs7630382 | 3 | T | C | 0.0130901 | NA | 2.33453E-12 | 0.00186658 | 407661 |
| rs3774063 | 3 | T | C | 0.0207635 | NA | 1.60842E-11 | 0.00308166 | 407661 |
| rs17639546 | 3 | A | G | -0.0175466 | NA | 1.65844E-11 | 0.00260594 | 407661 |
| rs6445258 | 3 | C | T | -0.0125792 | NA | 3.84698E-08 | 0.00228807 | 407661 |
| rs1454687 | 3 | G | C | -0.0181157 | NA | 1.96743E-22 | 0.00185927 | 407661 |
| rs12330631 | 3 | T | C | -0.0110388 | NA | 1.10583E-08 | 0.00193201 | 407661 |
| rs67481408 | 3 | TA | T | 0.0145727 | NA | 3.83354E-12 | 0.00209885 | 407661 |
| rs10936684 | 3 | A | G | -0.0115118 | NA | 6.48246E-09 | 0.00198349 | 407661 |
| rs11923804 | 3 | A | C | -0.012582 | NA | 2.44439E-10 | 0.00198754 | 407661 |
| rs7619139 | 3 | A | T | 0.0108844 | NA | 9.03005E-09 | 0.00189359 | 407661 |
| rs754635 | 3 | G | C | 0.017152 | NA | 4.0493E-09 | 0.00291593 | 407661 |
| rs62261725 | 3 | G | A | -0.0144853 | NA | 2.86286E-13 | 0.00198409 | 407661 |
| rs56398417 | 3 | T | C | -0.0132917 | NA | 3.46338E-11 | 0.00200618 | 407661 |
| rs654060 | 3 | A | T | 0.0108628 | NA | 5.76302E-09 | 0.00186533 | 407661 |
| rs1154988 | 3 | A | T | 0.0188249 | NA | 2.32756E-17 | 0.00222093 | 407661 |
| rs11917587 | 3 | A | G | 0.0154906 | NA | 1.70962E-16 | 0.00187973 | 407661 |
| rs13322435 | 3 | G | A | -0.0163938 | NA | 8.5055E-18 | 0.00190791 | 407661 |
| rs3113509 | 4 | T | C | -0.0127687 | NA | 1.27441E-09 | 0.00210337 | 407661 |
| rs6845883 | 4 | G | A | -0.0136018 | NA | 1.67865E-09 | 0.00225712 | 407661 |
| rs11099020 | 4 | T | C | -0.0110748 | NA | 1.1698E-08 | 0.00194157 | 407661 |
| rs9991695 | 4 | C | A | -0.0109181 | NA | 4.98127E-09 | 0.00186705 | 407661 |
| rs73213484 | 4 | T | A | -0.0181165 | NA | 1.53745E-11 | 0.00268619 | 407661 |
| rs10938398 | 4 | A | G | 0.0206341 | NA | 4.61318E-28 | 0.00187873 | 407661 |
| rs6815910 | 4 | A | T | -0.0101967 | NA | 4.87832E-08 | 0.001869 | 407661 |
| rs4148155 | 4 | G | A | -0.0161582 | NA | 3.13971E-08 | 0.00292008 | 407661 |
| rs7377083 | 4 | A | C | 0.01312 | NA | 3.67029E-12 | 0.00188796 | 407661 |
| rs58449180 | 4 | TA | T | -0.0108609 | NA | 5.53261E-09 | 0.00186283 | 407661 |
| rs1229984 | 4 | C | T | 0.0380954 | NA | 1.2716E-09 | 0.00627503 | 407661 |
| rs57800857 | 4 | C | A | -0.0143522 | NA | 1.33783E-13 | 0.00193887 | 407661 |
| rs1522569 | 4 | G | T | -0.0133624 | NA | 2.80886E-08 | 0.00240636 | 407661 |
| rs56203712 | 4 | G | A | -0.0153329 | NA | 6.85646E-12 | 0.00223491 | 407661 |
| rs12506689 | 4 | A | G | 0.0121834 | NA | 2.75081E-10 | 0.00193014 | 407661 |
| rs66679256 | 4 | T | C | 0.0143993 | NA | 1.42298E-14 | 0.00187143 | 407661 |
| rs4419475 | 4 | T | A | 0.0113284 | NA | 1.99251E-09 | 0.00188856 | 407661 |
| rs4525978 | 4 | T | C | -0.0115435 | NA | 4.90253E-08 | 0.00211621 | 407661 |
| rs57590313 | 4 | A | C | 0.0146884 | NA | 1.26398E-09 | 0.00241907 | 407661 |
| rs1724557 | 4 | A | C | -0.0125658 | NA | 3.33426E-11 | 0.00189502 | 407661 |
| rs67913249 | 5 | G | C | -0.0130331 | NA | 3.17468E-11 | 0.00196334 | 407661 |
| rs3776712 | 5 | C | T | -0.0116152 | NA | 4.00415E-08 | 0.00211544 | 407661 |
| rs36156 | 5 | C | T | 0.0119633 | NA | 7.56676E-10 | 0.00194407 | 407661 |
| rs201632662 | 5 | C | CGG | 0.0273676 | NA | 1.83992E-23 | 0.00274188 | 407661 |
| rs4958702 | 5 | C | T | -0.011939 | NA | 2.04207E-10 | 0.00187774 | 407661 |
| rs2546503 | 5 | T | A | -0.0113517 | NA | 4.41845E-09 | 0.00193461 | 407661 |
| rs2307111 | 5 | C | T | -0.0239454 | NA | 2.79576E-36 | 0.00190378 | 407661 |
| rs407028 | 5 | A | T | 0.0129505 | NA | 8.5426E-09 | 0.00224938 | 407661 |
| rs12514473 | 5 | C | T | -0.0148009 | NA | 7.02749E-12 | 0.00215848 | 407661 |
| rs67632512 | 5 | A | C | 0.0170891 | NA | 5.01095E-09 | 0.00292281 | 407661 |
| rs1625623 | 5 | T | C | 0.0108468 | NA | 2.5204E-08 | 0.00194671 | 407661 |
| rs6884859 | 5 | C | G | -0.0129116 | NA | 1.02014E-08 | 0.00225437 | 407661 |
| rs112323962 | 5 | AC | A | 0.0199841 | NA | 5.04789E-09 | 0.00341866 | 407661 |
| rs10461444 | 5 | T | A | 0.0106697 | NA | 1.76856E-08 | 0.00189407 | 407661 |
| rs61023343 | 5 | C | A | 0.0113381 | NA | 5.14257E-09 | 0.00194062 | 407661 |
| rs254027 | 5 | A | T | 0.013477 | NA | 7.54571E-13 | 0.00187985 | 407661 |
| rs40067 | 5 | A | G | -0.0177159 | NA | 8.62581E-13 | 0.00247747 | 407661 |
| rs12516350 | 5 | G | A | -0.0114715 | NA | 7.98068E-10 | 0.00186671 | 407661 |
| rs13186637 | 5 | C | T | -0.0136219 | NA | 3.10099E-12 | 0.00195351 | 407661 |
| rs7707394 | 5 | A | G | -0.0181054 | NA | 1.02353E-20 | 0.00193981 | 407661 |
| rs34732995 | 5 | CTA | C | -0.0134553 | NA | 5.25775E-13 | 0.00186401 | 407661 |
| rs13163306 | 5 | A | G | -0.0110365 | NA | 3.17475E-09 | 0.00186355 | 407661 |
| rs2133561 | 5 | T | A | -0.0131113 | NA | 1.02849E-11 | 0.00192743 | 407661 |
| rs245767 | 5 | G | A | 0.0149359 | NA | 1.02612E-12 | 0.00209568 | 407661 |
| rs11759152 | 6 | A | G | 0.0151099 | NA | 4.61381E-08 | 0.00276456 | 407661 |
| rs1321521 | 6 | A | C | 0.0131604 | NA | 1.54028E-11 | 0.00195141 | 407661 |
| rs9370243 | 6 | T | G | 0.0199682 | NA | 4.74428E-09 | 0.00340993 | 407661 |
| rs10945159 | 6 | T | C | 0.0132671 | NA | 1.1337E-10 | 0.00205757 | 407661 |
| rs678278 | 6 | G | A | -0.0107389 | NA | 8.27942E-09 | 0.00186352 | 407661 |
| rs60898782 | 6 | A | G | 0.0150576 | NA | 9.9747E-11 | 0.00232826 | 407661 |
| rs72892910 | 6 | T | G | 0.0314001 | NA | 7.40628E-37 | 0.00247589 | 407661 |
| rs1327259 | 6 | G | A | -0.0107205 | NA | 2.18369E-08 | 0.00191548 | 407661 |
| rs13210406 | 6 | G | C | -0.0123251 | NA | 1.9066E-09 | 0.00205227 | 407661 |
| rs2253310 | 6 | G | C | 0.0199736 | NA | 2.7555E-25 | 0.0019224 | 407661 |
| rs9478496 | 6 | C | T | 0.0148008 | NA | 3.87597E-09 | 0.00251312 | 407661 |
| rs2066295 | 6 | G | A | -0.0228124 | NA | 7.22104E-25 | 0.0022153 | 407661 |
| rs34045288 | 6 | T | C | 0.0207973 | NA | 4.23838E-26 | 0.00196813 | 407661 |
| rs9294260 | 6 | A | G | 0.0128048 | NA | 7.56484E-12 | 0.00187026 | 407661 |
| rs3004179 | 6 | G | A | 0.0117745 | NA | 5.19912E-10 | 0.00189514 | 407661 |
| rs72959041 | 6 | A | G | 0.0301906 | NA | 2.4632E-12 | 0.00430962 | 407661 |
| rs62395827 | 6 | T | C | -0.023172 | NA | 6.03254E-14 | 0.00308657 | 407661 |
| rs62407562 | 6 | A | T | 0.0143181 | NA | 8.25087E-12 | 0.00209509 | 407661 |
| rs2744938 | 6 | G | A | 0.0305147 | NA | 2.18323E-31 | 0.00261833 | 407661 |
| rs1902066 | 6 | C | T | 0.0106493 | NA | 1.53501E-08 | 0.0018823 | 407661 |
| rs12199502 | 6 | C | T | -0.0142156 | NA | 9.51634E-09 | 0.00247696 | 407661 |
| rs13210756 | 6 | A | T | -0.0175637 | NA | 4.95656E-10 | 0.00282354 | 407661 |
| rs10947793 | 6 | G | A | -0.0122259 | NA | 2.53519E-10 | 0.00193301 | 407661 |
| rs11757278 | 6 | C | T | -0.0135678 | NA | 1.79515E-11 | 0.00201848 | 407661 |
| rs112553547 | 6 | CAAAACA | C | -0.0147711 | NA | 3.14934E-08 | 0.00266967 | 407661 |
| rs215669 | 7 | A | G | -0.0129234 | NA | 1.46656E-11 | 0.00191424 | 407661 |
| rs12718572 | 7 | T | C | -0.0120621 | NA | 2.07429E-10 | 0.00189783 | 407661 |
| rs2470946 | 7 | T | G | 0.0111576 | NA | 3.65132E-09 | 0.00189135 | 407661 |
| rs10248298 | 7 | A | C | 0.0131809 | NA | 7.8886E-12 | 0.00192688 | 407661 |
| rs1805123 | 7 | G | T | -0.0134472 | NA | 4.36305E-10 | 0.00215484 | 407661 |
| rs1182199 | 7 | A | C | -0.0144063 | NA | 9.08448E-13 | 0.00201663 | 407661 |
| rs2289379 | 7 | T | C | -0.0105034 | NA | 3.54275E-08 | 0.00190547 | 407661 |
| rs2404324 | 7 | G | A | -0.0186094 | NA | 4.47816E-13 | 0.00257027 | 407661 |
| rs12375196 | 7 | A | C | 0.0127804 | NA | 1.31704E-11 | 0.0018887 | 407661 |
| rs73068448 | 7 | T | C | -0.01532 | NA | 8.45006E-10 | 0.00249665 | 407661 |
| rs10269774 | 7 | A | G | 0.0129258 | NA | 7.40969E-11 | 0.00198488 | 407661 |
| rs4722398 | 7 | T | C | 0.0162822 | NA | 1.67556E-09 | 0.00270177 | 407661 |
| rs4718964 | 7 | T | G | 0.0134909 | NA | 9.60285E-13 | 0.00189052 | 407661 |
| rs3030348 | 7 | CCTCAGTCT | C | -0.0161503 | NA | 2.67424E-11 | 0.0024237 | 407661 |
| rs236660 | 7 | C | T | 0.0176701 | NA | 1.18359E-19 | 0.00194806 | 407661 |
| rs17149254 | 7 | C | T | -0.0159031 | NA | 4.04762E-11 | 0.00240871 | 407661 |
| rs1922879 | 7 | A | G | -0.0110155 | NA | 2.22751E-08 | 0.00196939 | 407661 |
| rs1559900 | 8 | T | C | 0.0129202 | NA | 3.225E-10 | 0.00205489 | 407661 |
| rs11778934 | 8 | G | C | -0.0133447 | NA | 8.66164E-13 | 0.00186632 | 407661 |
| rs4876611 | 8 | G | A | 0.0160928 | NA | 5.97998E-15 | 0.00206202 | 407661 |
| rs117176448 | 8 | G | C | 0.021495 | NA | 9.0615E-12 | 0.00315146 | 407661 |
| rs59607297 | 8 | C | T | 0.0127954 | NA | 3.0987E-08 | 0.00231141 | 407661 |
| rs10957088 | 8 | C | T | 0.0149176 | NA | 3.83204E-09 | 0.00253215 | 407661 |
| rs7845090 | 8 | A | G | -0.0208089 | NA | 4.87865E-24 | 0.0020578 | 407661 |
| rs201519328 | 8 | A | G | 0.0159828 | NA | 1.46589E-17 | 0.00187376 | 407661 |
| rs12680342 | 8 | G | T | -0.0126567 | NA | 9.8168E-09 | 0.00220737 | 407661 |
| rs10954772 | 8 | C | T | -0.0146578 | NA | 3.23445E-13 | 0.00201226 | 407661 |
| rs13264909 | 8 | T | A | -0.0123269 | NA | 5.17249E-11 | 0.00187742 | 407661 |
| rs4072917 | 8 | A | G | 0.0106757 | NA | 1.05254E-08 | 0.00186572 | 407661 |
| rs10780248 | 9 | A | G | -0.0110596 | NA | 3.45486E-09 | 0.00187186 | 407661 |
| rs16916303 | 9 | G | A | -0.0169347 | NA | 4.48384E-09 | 0.00288728 | 407661 |
| rs7038934 | 9 | C | T | 0.0111507 | NA | 5.35156E-09 | 0.00191072 | 407661 |
| rs1752169 | 9 | A | C | 0.0144599 | NA | 1.7362E-11 | 0.00214965 | 407661 |
| rs12001634 | 9 | A | T | -0.012913 | NA | 5.11564E-11 | 0.0019662 | 407661 |
| rs10756713 | 9 | G | A | -0.0150071 | NA | 1.36082E-15 | 0.00187848 | 407661 |
| rs17770336 | 9 | T | C | 0.0191746 | NA | 3.63831E-22 | 0.00198068 | 407661 |
| rs12339822 | 9 | G | A | 0.0116343 | NA | 5.10434E-10 | 0.00187171 | 407661 |
| rs2417998 | 9 | G | C | -0.0132738 | NA | 8.6896E-11 | 0.00204585 | 407661 |
| rs17218879 | 9 | G | C | 0.011149 | NA | 1.53169E-08 | 0.00197049 | 407661 |
| rs4743930 | 9 | T | C | 0.0120016 | NA | 2.15452E-08 | 0.00214348 | 407661 |
| rs1019240 | 9 | T | A | 0.0112324 | NA | 7.60887E-09 | 0.00194436 | 407661 |
| rs6597653 | 9 | C | G | 0.0114969 | NA | 1.57529E-09 | 0.00190457 | 407661 |
| rs2439823 | 10 | G | A | 0.0154423 | NA | 1.45345E-16 | 0.00186947 | 407661 |
| rs7070670 | 10 | T | C | -0.0117059 | NA | 3.94748E-09 | 0.00198865 | 407661 |
| rs35475612 | 10 | CTT | C | 0.0137241 | NA | 8.00571E-12 | 0.0020069 | 407661 |
| rs7894565 | 10 | C | T | 0.0204287 | NA | 3.08248E-24 | 0.00201128 | 407661 |
| rs35972789 | 10 | A | C | -0.0292226 | NA | 2.10363E-09 | 0.00487889 | 407661 |
| rs12416587 | 10 | A | T | 0.0167914 | NA | 4.19952E-11 | 0.00254536 | 407661 |
| rs1571136 | 10 | T | C | 0.0108151 | NA | 8.70923E-09 | 0.00187955 | 407661 |
| rs10787738 | 10 | T | C | 0.0144743 | NA | 2.23049E-11 | 0.00216356 | 407661 |
| rs2007833 | 10 | G | A | -0.0127211 | NA | 3.27831E-08 | 0.00230209 | 407661 |
| rs17399739 | 10 | G | A | 0.0238767 | NA | 5.99377E-11 | 0.0036487 | 407661 |
| rs17094222 | 10 | C | T | 0.0143328 | NA | 3.00497E-10 | 0.00227558 | 407661 |
| rs2172131 | 10 | C | T | -0.0126836 | NA | 1.60177E-11 | 0.00188229 | 407661 |
| rs7925100 | 11 | A | G | 0.0124779 | NA | 5.05825E-11 | 0.00189944 | 407661 |
| rs61903695 | 11 | G | A | 0.0125342 | NA | 3.8892E-09 | 0.00212847 | 407661 |
| rs188351692 | 11 | T | C | 0.0254743 | NA | 3.40997E-10 | 0.00405714 | 407661 |
| rs2187449 | 11 | A | G | 0.0120805 | NA | 4.6237E-08 | 0.00221044 | 407661 |
| rs11030108 | 11 | G | A | -0.0248117 | NA | 7.15979E-36 | 0.00198441 | 407661 |
| rs7115013 | 11 | T | C | -0.0108313 | NA | 7.37021E-09 | 0.0018732 | 407661 |
| rs11216221 | 11 | G | A | 0.0245173 | NA | 1.54064E-09 | 0.00405912 | 407661 |
| rs10894605 | 11 | G | A | 0.0111058 | NA | 3.68036E-09 | 0.00188298 | 407661 |
| rs111258054 | 11 | T | C | 0.0165052 | NA | 1.00046E-11 | 0.00242493 | 407661 |
| rs7941828 | 11 | T | C | -0.0163651 | NA | 2.48199E-17 | 0.00193243 | 407661 |
| rs7117115 | 11 | G | A | -0.020044 | NA | 3.13906E-26 | 0.00189181 | 407661 |
| rs186234792 | 11 | G | A | 0.0309274 | NA | 4.82236E-08 | 0.0056667 | 407661 |
| rs10898330 | 11 | T | C | -0.0111934 | NA | 2.07941E-09 | 0.00186823 | 407661 |
| rs11607476 | 11 | C | A | 0.0106982 | NA | 1.06468E-08 | 0.00187029 | 407661 |
| rs10128597 | 11 | A | G | -0.0166279 | NA | 2.10814E-15 | 0.00209557 | 407661 |
| rs11824092 | 11 | C | T | 0.0139447 | NA | 7.32993E-13 | 0.00194401 | 407661 |
| rs11037563 | 11 | C | T | 0.0148192 | NA | 1.16225E-14 | 0.00191956 | 407661 |
| rs7952436 | 11 | T | C | -0.0268741 | NA | 1.21507E-15 | 0.00335804 | 407661 |
| rs883528 | 12 | C | T | 0.0124716 | NA | 9.24251E-10 | 0.00203718 | 407661 |
| rs1160863 | 12 | TTA | T | 0.0142377 | NA | 2.56153E-14 | 0.00186878 | 407661 |
| rs7138383 | 12 | A | G | -0.0158782 | NA | 1.1051E-13 | 0.0021377 | 407661 |
| rs111260184 | 12 | A | G | 0.0102452 | NA | 4.39026E-08 | 0.00187148 | 407661 |
| rs77234932 | 12 | T | C | -0.0165087 | NA | 1.75267E-13 | 0.00224106 | 407661 |
| rs147786161 | 12 | G | A | 0.011882 | NA | 2.75074E-10 | 0.00188238 | 407661 |
| rs57307148 | 12 | A | C | 0.018511 | NA | 3.19301E-16 | 0.00226689 | 407661 |
| rs76895963 | 12 | G | T | 0.043494 | NA | 1.4785E-09 | 0.00719302 | 407661 |
| rs12367809 | 12 | T | C | 0.0219231 | NA | 7.97627E-30 | 0.00193264 | 407661 |
| rs894736 | 12 | G | A | 0.0163291 | NA | 3.39313E-17 | 0.00193652 | 407661 |
| rs55938344 | 12 | C | A | -0.0137001 | NA | 3.19029E-10 | 0.00217834 | 407661 |
| rs2731238 | 12 | T | G | -0.0232909 | NA | 8.86339E-09 | 0.00404977 | 407661 |
| rs11056876 | 12 | G | A | 0.013053 | NA | 1.48946E-08 | 0.00230506 | 407661 |
| rs10506965 | 12 | G | T | -0.0158288 | NA | 2.48714E-17 | 0.00186915 | 407661 |
| rs184287715 | 12 | G | T | 0.0124248 | NA | 1.61585E-08 | 0.00219954 | 407661 |
| rs6489071 | 12 | G | C | 0.0162836 | NA | 7.85236E-15 | 0.00209572 | 407661 |
| rs2306537 | 12 | G | A | 0.0135199 | NA | 1.59478E-11 | 0.00200622 | 407661 |
| rs9536637 | 13 | G | T | -0.0190168 | NA | 7.64892E-11 | 0.00292236 | 407661 |
| rs1441264 | 13 | A | G | 0.0151611 | NA | 3.77485E-15 | 0.00192836 | 407661 |
| rs61969510 | 13 | C | T | 0.0125993 | NA | 1.42672E-09 | 0.00208168 | 407661 |
| rs12877270 | 13 | A | G | 0.0136897 | NA | 3.6686E-13 | 0.00188375 | 407661 |
| rs9888533 | 13 | T | C | 0.0108188 | NA | 1.05962E-08 | 0.0018911 | 407661 |
| rs2528787 | 13 | T | C | -0.0126455 | NA | 5.724E-11 | 0.00193039 | 407661 |
| rs1218822 | 13 | A | G | 0.0137486 | NA | 2.44794E-12 | 0.00196234 | 407661 |
| rs1336486 | 13 | G | T | 0.0127375 | NA | 1.14934E-10 | 0.00197607 | 407661 |
| rs927606 | 13 | T | C | -0.0115761 | NA | 4.30487E-10 | 0.00185438 | 407661 |
| rs9568867 | 13 | A | G | 0.0215852 | NA | 9.00741E-15 | 0.00278428 | 407661 |
| rs4055791 | 13 | T | C | -0.0130472 | NA | 4.00313E-12 | 0.0018808 | 407661 |
| rs4435118 | 13 | A | G | 0.0132194 | NA | 1.25052E-09 | 0.00217652 | 407661 |
| rs2026752 | 13 | T | G | 0.0134813 | NA | 3.33534E-10 | 0.00214591 | 407661 |
| rs4900715 | 14 | A | G | -0.0117012 | NA | 3.05225E-10 | 0.00185848 | 407661 |
| rs217669 | 14 | C | T | 0.0128558 | NA | 7.027E-10 | 0.00208513 | 407661 |
| rs2370982 | 14 | T | C | 0.0223143 | NA | 9.70957E-23 | 0.0022735 | 407661 |
| rs61992671 | 14 | G | A | -0.0140485 | NA | 4.14095E-13 | 0.00193749 | 407661 |
| rs6575340 | 14 | A | G | 0.016753 | NA | 4.54988E-18 | 0.0019336 | 407661 |
| rs10132280 | 14 | A | C | -0.0164064 | NA | 7.18952E-16 | 0.0020337 | 407661 |
| rs4981693 | 14 | A | G | 0.0166678 | NA | 5.5847E-14 | 0.00221721 | 407661 |
| rs11160601 | 14 | T | C | 0.0201436 | NA | 4.31907E-10 | 0.00322709 | 407661 |
| rs3993347 | 14 | C | T | -0.0137963 | NA | 1.25516E-12 | 0.00194337 | 407661 |
| rs10153031 | 15 | G | T | 0.0182365 | NA | 1.09245E-21 | 0.00190604 | 407661 |
| rs7163176 | 15 | A | G | -0.011901 | NA | 1.9196E-10 | 0.00186898 | 407661 |
| rs2456524 | 15 | T | C | 0.0150439 | NA | 1.10831E-08 | 0.00263316 | 407661 |
| rs11071546 | 15 | C | T | 0.0115621 | NA | 6.67636E-10 | 0.00187283 | 407661 |
| rs7171864 | 15 | A | G | 0.0151922 | NA | 1.22208E-14 | 0.0019695 | 407661 |
| rs34994596 | 15 | C | T | -0.0137728 | NA | 1.08019E-11 | 0.00202678 | 407661 |
| rs7179507 | 15 | A | G | 0.0139805 | NA | 2.41435E-12 | 0.00199488 | 407661 |
| rs4586381 | 15 | A | C | -0.0107604 | NA | 2.54431E-08 | 0.00193177 | 407661 |
| rs8024137 | 15 | T | A | 0.0144696 | NA | 2.43276E-08 | 0.00259404 | 407661 |
| rs57066717 | 15 | GAC | G | -0.0135636 | NA | 6.52379E-11 | 0.00207674 | 407661 |
| rs11636611 | 15 | T | C | 0.0112866 | NA | 1.2286E-09 | 0.00185743 | 407661 |
| rs2238435 | 16 | G | C | 0.0187936 | NA | 1.02212E-22 | 0.0019158 | 407661 |
| rs3814883 | 16 | T | C | 0.0254681 | NA | 2.1424E-42 | 0.00186639 | 407661 |
| rs2962082 | 16 | A | G | -0.0102614 | NA | 3.65595E-08 | 0.00186344 | 407661 |
| rs369230 | 16 | T | G | 0.0110328 | NA | 4.88326E-08 | 0.00202233 | 407661 |
| rs629471 | 16 | G | A | 0.0123161 | NA | 2.06847E-09 | 0.00205531 | 407661 |
| rs56094641 | 16 | G | A | 0.0566533 | NA | 1.8967E-196 | 0.00189468 | 407661 |
| rs756717 | 16 | A | G | -0.0115846 | NA | 1.61243E-09 | 0.00192031 | 407661 |
| rs12926311 | 16 | C | G | -0.0135288 | NA | 3.79577E-12 | 0.00194811 | 407661 |
| rs116353685 | 16 | C | G | -0.0228163 | NA | 6.75461E-21 | 0.00243309 | 407661 |
| rs28375268 | 16 | T | G | -0.0142212 | NA | 3.43954E-13 | 0.00195455 | 407661 |
| rs7498665 | 16 | G | A | 0.0246546 | NA | 1.32678E-38 | 0.00189741 | 407661 |
| rs1073331 | 16 | G | A | -0.011642 | NA | 6.23807E-10 | 0.00188252 | 407661 |
| rs825680 | 16 | T | A | -0.0108035 | NA | 1.23558E-08 | 0.0018971 | 407661 |
| rs12103006 | 16 | G | A | 0.0131917 | NA | 2.26569E-12 | 0.00187993 | 407661 |
| rs10530053 | 16 | CTTTTT | C | 0.0180594 | NA | 1.57398E-21 | 0.00189502 | 407661 |
| rs7206608 | 16 | G | C | 0.0130071 | NA | 6.29506E-11 | 0.00198989 | 407661 |
| rs731540 | 17 | A | G | 0.0126877 | NA | 2.99668E-10 | 0.00201425 | 407661 |
| rs8078135 | 17 | T | C | -0.0112572 | NA | 1.50041E-09 | 0.00186244 | 407661 |
| rs144443274 | 17 | T | C | 0.0182195 | NA | 2.90068E-13 | 0.00249619 | 407661 |
| rs8074454 | 17 | C | G | 0.0136942 | NA | 4.18504E-12 | 0.00197585 | 407661 |
| rs55931203 | 17 | T | C | 0.0242122 | NA | 6.68498E-24 | 0.0024017 | 407661 |
| rs4790841 | 17 | T | C | -0.0229415 | NA | 4.61424E-19 | 0.00257159 | 407661 |
| rs7210186 | 17 | G | A | 0.0121052 | NA | 1.65764E-09 | 0.00200809 | 407661 |
| rs6416913 | 17 | C | T | -0.0117898 | NA | 1.63995E-08 | 0.00208808 | 407661 |
| rs113866544 | 17 | C | T | 0.028472 | NA | 1.42922E-14 | 0.00370068 | 407661 |
| rs35867081 | 17 | G | A | -0.0113488 | NA | 1.14797E-09 | 0.00186432 | 407661 |
| rs9916444 | 17 | G | C | 0.0127609 | NA | 8.01863E-11 | 0.00196314 | 407661 |
| rs62072003 | 17 | T | C | 0.0146714 | NA | 2.97119E-08 | 0.00264677 | 407661 |
| rs3826408 | 17 | T | C | 0.0113154 | NA | 1.17147E-09 | 0.00185983 | 407661 |
| rs12951079 | 17 | A | G | -0.0155826 | NA | 2.19989E-16 | 0.00189785 | 407661 |
| rs11653367 | 17 | G | A | -0.0182133 | NA | 4.8899E-20 | 0.00198696 | 407661 |
| rs1652376 | 18 | T | G | -0.0191391 | NA | 8.16018E-25 | 0.00186072 | 407661 |
| rs6567160 | 18 | C | T | 0.0431506 | NA | 2.76885E-86 | 0.00219179 | 407661 |
| rs635132 | 18 | G | A | 0.0123322 | NA | 4.91994E-09 | 0.00210813 | 407661 |
| rs77162980 | 18 | C | A | -0.0536372 | NA | 2.71957E-21 | 0.00566212 | 407661 |
| rs60764613 | 18 | T | G | 0.0170996 | NA | 1.08131E-10 | 0.002649 | 407661 |
| rs491342 | 18 | A | G | 0.0132991 | NA | 3.35892E-11 | 0.00200593 | 407661 |
| rs2861344 | 18 | A | G | 0.0115565 | NA | 4.81504E-10 | 0.00185646 | 407661 |
| rs1942826 | 18 | A | G | 0.0171312 | NA | 1.18754E-09 | 0.00281675 | 407661 |
| rs1834144 | 18 | A | C | -0.0143554 | NA | 8.12831E-14 | 0.0019222 | 407661 |
| rs35343344 | 19 | A | C | -0.0179443 | NA | 4.12572E-17 | 0.00213387 | 407661 |
| rs11878507 | 19 | A | G | -0.0146738 | NA | 3.21514E-15 | 0.00186162 | 407661 |
| rs11670190 | 19 | G | C | 0.0118768 | NA | 1.58034E-10 | 0.00185649 | 407661 |
| rs56187939 | 19 | G | A | -0.0120003 | NA | 2.72502E-09 | 0.00201775 | 407661 |
| rs12461072 | 19 | G | C | 0.0196605 | NA | 7.43704E-22 | 0.00204638 | 407661 |
| rs111640872 | 19 | C | G | 0.0194137 | NA | 9.22359E-23 | 0.00197693 | 407661 |
| rs10422861 | 19 | T | C | 0.0110548 | NA | 2.00724E-08 | 0.00197007 | 407661 |
| rs7259070 | 19 | C | T | 0.0169678 | NA | 6.45357E-19 | 0.00190994 | 407661 |
| rs56356382 | 19 | C | T | -0.0166271 | NA | 1.92486E-12 | 0.00236186 | 407661 |
| rs429358 | 19 | C | T | -0.0247647 | NA | 3.434E-22 | 0.00255656 | 407661 |
| rs10423928 | 19 | A | T | -0.0254175 | NA | 1.92752E-27 | 0.00234196 | 407661 |
| rs6030803 | 20 | C | T | -0.0186385 | NA | 3.76357E-11 | 0.00281843 | 407661 |
| rs1056441 | 20 | C | T | 0.0134395 | NA | 1.07647E-11 | 0.0019776 | 407661 |
| rs35679975 | 20 | C | T | -0.0244469 | NA | 3.78966E-25 | 0.00235985 | 407661 |
| rs4456769 | 20 | T | C | 0.01315 | NA | 2.16721E-11 | 0.00196436 | 407661 |
| rs76040172 | 21 | A | G | -0.031822 | NA | 8.02786E-15 | 0.00409701 | 407661 |
| rs13047416 | 21 | G | C | -0.0128153 | NA | 2.43164E-11 | 0.0019192 | 407661 |
| rs11704728 | 22 | T | C | 0.0150712 | NA | 1.20648E-10 | 0.00234079 | 407661 |
| rs165722 | 22 | T | C | 0.0105585 | NA | 1.52669E-08 | 0.00186594 | 407661 |
| rs28489620 | 22 | A | G | -0.0127224 | NA | 5.88098E-10 | 0.00205412 | 407661 |
| 241 SNPs are associated with waist-to-hip ratio | | | | | | | | |
| SNP | chr.exposure | effect_allele.exposure | other_allele.exposure | beta.exposure | eaf.exposure | pval.exposure | se.exposure | samplesize.exposure |
| rs9435732 | 1 | T | C | -0.0144445 | 0.261123 | 1.7E-10 | 0.00225353 | 458349 |
| rs4908286 | 1 | T | G | 0.0163108 | 0.601913 | 1.10002E-15 | 0.00202487 | 458349 |
| rs905938 | 1 | C | T | -0.0246181 | 0.264989 | 1.69981E-27 | 0.00224841 | 458349 |
| rs2605110 | 1 | T | C | 0.0366572 | 0.671975 | 1.50003E-68 | 0.00211101 | 458349 |
| rs12121476 | 1 | G | C | 0.0223986 | 0.249589 | 5.70033E-22 | 0.00231085 | 458349 |
| rs2298632 | 1 | T | C | -0.015731 | 0.497671 | 4.79954E-15 | 0.00200341 | 458349 |
| rs1211184 | 1 | C | T | 0.0185414 | 0.0932957 | 2.19999E-08 | 0.00340717 | 458349 |
| rs1149392 | 1 | G | A | -0.015291 | 0.258651 | 2.49977E-12 | 0.00226662 | 458349 |
| rs10753801 | 1 | G | A | 0.0269491 | 0.839013 | 5.50047E-24 | 0.00269537 | 458349 |
| rs3795262 | 1 | G | T | 0.0208004 | 0.183636 | 1.9002E-16 | 0.00258388 | 458349 |
| rs41287298 | 1 | A | T | -0.026188 | 0.0537514 | 3.09999E-09 | 0.00440842 | 458349 |
| rs9425291 | 1 | A | G | 0.0284446 | 0.436459 | 1.50003E-47 | 0.00199971 | 458349 |
| rs11590845 | 1 | C | A | 0.0109623 | 0.35562 | 2.30001E-08 | 0.00207252 | 458349 |
| rs366187 | 1 | A | G | 0.0148343 | 0.402538 | 4.90004E-14 | 0.00201959 | 458349 |
| rs984222 | 1 | G | C | 0.0353383 | 0.611148 | 6.09958E-70 | 0.0020349 | 458349 |
| rs6701725 | 1 | G | A | 0.0112381 | 0.596058 | 6.1E-09 | 0.00202199 | 458349 |
| rs6670040 | 1 | T | C | -0.0107477 | 0.501555 | 0.00000004 | 0.00198478 | 458349 |
| rs12141651 | 1 | G | A | 0.0210784 | 0.178703 | 3.50026E-16 | 0.00258668 | 458349 |
| rs3851294 | 1 | G | A | 0.0237595 | 0.904934 | 4.40048E-12 | 0.00343617 | 458349 |
| rs2039956 | 1 | A | C | 0.0168169 | 0.293679 | 7.00003E-14 | 0.0021752 | 458349 |
| rs7578597 | 2 | C | T | 0.020388 | 0.108373 | 6.89922E-11 | 0.00319438 | 458349 |
| rs58584712 | 2 | A | G | -0.0248956 | 0.211128 | 1.50003E-24 | 0.00243457 | 458349 |
| rs10195252 | 2 | C | T | -0.0313223 | 0.405292 | 1.10002E-54 | 0.00201975 | 458349 |
| rs3771084 | 2 | G | A | 0.0203044 | 0.557977 | 3.19963E-23 | 0.00202393 | 458349 |
| rs16857751 | 2 | T | C | 0.0183917 | 0.0972302 | 2.39999E-08 | 0.00334775 | 458349 |
| rs72986327 | 2 | G | A | -0.0173758 | 0.167594 | 1.5E-10 | 0.00265482 | 458349 |
| rs2304429 | 2 | T | C | -0.0139591 | 0.584281 | 2.29985E-12 | 0.00203446 | 458349 |
| rs17033861 | 2 | A | G | 0.0197208 | 0.201405 | 8.30042E-16 | 0.00247662 | 458349 |
| rs4850910 | 2 | A | C | -0.0185986 | 0.816816 | 3.40017E-13 | 0.00256331 | 458349 |
| rs4321376 | 2 | T | C | 0.0156839 | 0.214419 | 1.59993E-11 | 0.00242427 | 458349 |
| rs2509902 | 2 | C | T | 0.0200535 | 0.858426 | 2.60016E-12 | 0.00284218 | 458349 |
| rs3732083 | 2 | C | T | 0.014327 | 0.43 | 7.8001E-13 | 0.00201616 | 458349 |
| rs6716270 | 2 | C | T | 0.0250618 | 0.147563 | 3.29989E-19 | 0.00279117 | 458349 |
| rs78852278 | 2 | A | C | -0.0239901 | 0.0908653 | 4.40048E-12 | 0.00344824 | 458349 |
| rs651477 | 2 | C | T | 0.0114968 | 0.256505 | 3.29997E-08 | 0.00226947 | 458349 |
| rs6735844 | 2 | T | C | 0.0170057 | 0.269378 | 2.80027E-14 | 0.0022359 | 458349 |
| rs1250247 | 2 | G | C | -0.0150832 | 0.716609 | 2.60016E-11 | 0.00222256 | 458349 |
| rs2459337 | 2 | C | G | -0.0176158 | 0.78659 | 2.90001E-13 | 0.00242044 | 458349 |
| rs6546355 | 2 | G | A | -0.0115281 | 0.717646 | 3.09999E-08 | 0.00220726 | 458349 |
| rs55920843 | 2 | G | T | -0.0810929 | 0.0118043 | 4.60045E-18 | 0.0093685 | 458349 |
| rs2629046 | 2 | C | T | -0.0111891 | 0.455334 | 1.89998E-08 | 0.00199038 | 458349 |
| rs1727956 | 3 | A | G | -0.0126863 | 0.727096 | 1.09999E-08 | 0.00225088 | 458349 |
| rs900400 | 3 | C | T | -0.0317467 | 0.399368 | 3.29989E-58 | 0.00203163 | 458349 |
| rs4684859 | 3 | A | G | 0.0245372 | 0.423559 | 1.59993E-35 | 0.00201001 | 458349 |
| rs2276824 | 3 | G | C | -0.0221456 | 0.542187 | 9.70063E-30 | 0.00200536 | 458349 |
| rs4928143 | 3 | T | C | 0.0249458 | 0.0772705 | 6.79986E-12 | 0.00371771 | 458349 |
| rs2271494 | 3 | T | A | 0.0104663 | 0.41343 | 4.09996E-08 | 0.0020274 | 458349 |
| rs72628506 | 3 | A | G | 0.0313714 | 0.0882821 | 8.80035E-20 | 0.00349789 | 458349 |
| rs7651090 | 3 | G | A | 0.0148676 | 0.314294 | 3.90032E-12 | 0.00213983 | 458349 |
| rs11718898 | 3 | C | T | -0.0170723 | 0.659018 | 3.19963E-16 | 0.00212843 | 458349 |
| rs333507 | 3 | A | G | -0.0143928 | 0.714684 | 8.30042E-11 | 0.00219788 | 458349 |
| rs9833879 | 3 | A | G | 0.0336708 | 0.784376 | 6.00067E-45 | 0.00241565 | 458349 |
| rs4683297 | 3 | C | T | -0.0151842 | 0.3309 | 2.90001E-13 | 0.00210864 | 458349 |
| rs1499813 | 3 | C | T | -0.0134792 | 0.413426 | 5.30029E-11 | 0.00202019 | 458349 |
| rs6793495 | 3 | A | G | -0.012712 | 0.266648 | 0.00000002 | 0.00224364 | 458349 |
| rs267567 | 3 | A | G | 0.0110553 | 0.467693 | 8.10009E-09 | 0.00198916 | 458349 |
| rs4681821 | 3 | C | T | 0.0130205 | 0.682538 | 3.2E-10 | 0.00213474 | 458349 |
| rs4132228 | 3 | T | C | -0.0384319 | 0.289003 | 7.19946E-70 | 0.00219266 | 458349 |
| rs9818870 | 3 | T | C | 0.0225004 | 0.156724 | 6.4998E-17 | 0.00273061 | 458349 |
| rs4450871 | 4 | G | A | -0.0172266 | 0.442894 | 6.4998E-17 | 0.00200449 | 458349 |
| rs2048508 | 4 | C | T | 0.0200193 | 0.175649 | 3.40017E-14 | 0.00261295 | 458349 |
| rs57826934 | 4 | T | C | 0.020197 | 0.327289 | 1.10002E-21 | 0.00211897 | 458349 |
| rs13107325 | 4 | T | C | -0.0351329 | 0.0748526 | 6.29941E-21 | 0.00383586 | 458349 |
| rs11737560 | 4 | T | C | 0.0175972 | 0.124267 | 3.50002E-09 | 0.00301027 | 458349 |
| rs11733546 | 4 | T | C | 0.0333311 | 0.0373727 | 2.09991E-11 | 0.00523959 | 458349 |
| rs951252 | 4 | G | A | 0.0195471 | 0.550041 | 1.9002E-23 | 0.00199825 | 458349 |
| rs308403 | 4 | T | C | -0.0182539 | 0.307566 | 7.00003E-18 | 0.00215341 | 458349 |
| rs113075206 | 4 | G | C | -0.0166073 | 0.451366 | 6.90081E-17 | 0.00205254 | 458349 |
| rs6836057 | 4 | G | T | -0.0125125 | 0.347203 | 8.30004E-10 | 0.00208856 | 458349 |
| rs2271384 | 4 | A | G | -0.0164369 | 0.601341 | 7.39946E-16 | 0.00203108 | 458349 |
| rs62320367 | 4 | T | C | 0.0146011 | 0.219844 | 7.10003E-10 | 0.00240038 | 458349 |
| rs1377290 | 4 | T | C | 0.0263338 | 0.464122 | 3.80014E-39 | 0.00199405 | 458349 |
| rs34402524 | 4 | G | T | -0.0194842 | 0.122937 | 8.60003E-11 | 0.0030458 | 458349 |
| rs6822892 | 4 | G | A | -0.0118836 | 0.327846 | 0.000000015 | 0.00211606 | 458349 |
| rs293101 | 5 | T | C | -0.0113504 | 0.59916 | 1.79999E-08 | 0.0020304 | 458349 |
| rs464605 | 5 | T | C | -0.0312481 | 0.746587 | 4.60045E-44 | 0.00227923 | 458349 |
| rs72801474 | 5 | A | G | -0.0294071 | 0.092178 | 1.50003E-19 | 0.00344258 | 458349 |
| rs6887628 | 5 | A | C | 0.0117432 | 0.346076 | 6.1E-09 | 0.0020965 | 458349 |
| rs299615 | 5 | A | G | -0.0164059 | 0.157288 | 8.50002E-10 | 0.00272823 | 458349 |
| rs76525389 | 5 | A | G | 0.0254137 | 0.0469545 | 3.59998E-08 | 0.00469762 | 458349 |
| rs7723040 | 5 | T | C | 0.0266114 | 0.0906231 | 1E-14 | 0.00346461 | 458349 |
| rs6861681 | 5 | A | G | 0.0290203 | 0.302074 | 1.59993E-41 | 0.00216171 | 458349 |
| rs12519145 | 5 | A | G | 0.0217587 | 0.23034 | 3.69999E-21 | 0.00236042 | 458349 |
| rs937218 | 5 | G | A | -0.0142749 | 0.450205 | 6.4003E-13 | 0.00199826 | 458349 |
| rs3898451 | 5 | A | C | -0.0136484 | 0.661605 | 2.49977E-11 | 0.00210134 | 458349 |
| rs10515337 | 5 | C | T | -0.0170845 | 0.314685 | 2.80027E-15 | 0.00213926 | 458349 |
| rs34005 | 5 | A | G | -0.011781 | 0.436 | 3.59998E-09 | 0.00200147 | 458349 |
| rs17631629 | 5 | T | C | 0.0199781 | 0.111673 | 2.80027E-11 | 0.00315146 | 458349 |
| rs1445846 | 5 | C | T | -0.0115093 | 0.662738 | 3.79997E-08 | 0.00211156 | 458349 |
| rs3792752 | 5 | G | A | 0.0143275 | 0.258278 | 4.70002E-11 | 0.00226882 | 458349 |
| rs7708285 | 5 | A | G | -0.013586 | 0.691461 | 0.000000002 | 0.00215108 | 458349 |
| rs250303 | 5 | G | A | 0.017323 | 0.296175 | 1.80011E-15 | 0.0021738 | 458349 |
| rs115447786 | 6 | T | C | 0.0850764 | 0.0446225 | 9.79941E-73 | 0.00481435 | 458349 |
| rs4711750 | 6 | A | T | 0.0494331 | 0.500015 | 8.3946E-142 | 0.00198607 | 458349 |
| rs67597629 | 6 | A | G | 0.0362621 | 0.0795641 | 5.30029E-23 | 0.00366919 | 458349 |
| rs35772466 | 6 | T | C | -0.0193403 | 0.493887 | 4.70002E-23 | 0.0019836 | 458349 |
| rs10046368 | 6 | C | T | -0.0180066 | 0.30225 | 4.70002E-17 | 0.00216521 | 458349 |
| rs1294421 | 6 | G | T | 0.0298455 | 0.606918 | 1.39991E-49 | 0.0020313 | 458349 |
| rs10498948 | 6 | T | G | 0.0224348 | 0.105556 | 3.19963E-12 | 0.00323109 | 458349 |
| rs72961013 | 6 | A | G | 0.122913 | 0.065846 | 1E-200 | 0.00400702 | 458349 |
| rs6921610 | 6 | T | C | 0.0125611 | 0.536888 | 3.29997E-10 | 0.00199256 | 458349 |
| rs9462026 | 6 | G | C | -0.0148829 | 0.327554 | 3.59998E-13 | 0.00211577 | 458349 |
| rs2073267 | 6 | G | A | 0.0270392 | 0.172639 | 8.60003E-26 | 0.00262938 | 458349 |
| rs651837 | 6 | G | A | -0.0238685 | 0.590708 | 1.9002E-32 | 0.00201756 | 458349 |
| rs555754 | 6 | A | G | -0.0226512 | 0.468052 | 1.80011E-30 | 0.00199677 | 458349 |
| rs6914079 | 6 | G | A | 0.0170385 | 0.814112 | 2.09991E-11 | 0.00255511 | 458349 |
| rs4487571 | 6 | A | C | 0.0302856 | 0.0655097 | 4.90004E-14 | 0.00403754 | 458349 |
| rs2242416 | 6 | G | A | -0.0154775 | 0.604127 | 7.19946E-15 | 0.0020478 | 458349 |
| rs77097175 | 6 | A | C | 0.0477698 | 0.0266171 | 8.69961E-15 | 0.00616525 | 458349 |
| rs3807947 | 7 | G | T | -0.0134256 | 0.394935 | 9.3994E-11 | 0.00203431 | 458349 |
| rs1055144 | 7 | T | C | 0.0291321 | 0.192834 | 4.00037E-31 | 0.00251975 | 458349 |
| rs2240463 | 7 | T | C | 0.0156213 | 0.343097 | 3.90032E-13 | 0.00210547 | 458349 |
| rs10953934 | 7 | A | G | -0.0157023 | 0.382768 | 8.9002E-15 | 0.00205406 | 458349 |
| rs10441228 | 7 | G | A | -0.0184168 | 0.498228 | 8.99912E-22 | 0.00198711 | 458349 |
| rs388781 | 7 | C | T | 0.0140472 | 0.385979 | 5.00035E-12 | 0.00204158 | 458349 |
| rs690238 | 7 | G | T | -0.0120008 | 0.35678 | 1.79999E-08 | 0.00209205 | 458349 |
| rs7797942 | 7 | G | T | -0.0141875 | 0.312752 | 5.50047E-11 | 0.0021414 | 458349 |
| rs1534696 | 7 | A | C | -0.026846 | 0.541111 | 1.99986E-42 | 0.00199498 | 458349 |
| rs849134 | 7 | G | A | -0.0194393 | 0.503042 | 1E-23 | 0.00198622 | 458349 |
| rs3110697 | 7 | G | A | -0.0121951 | 0.577723 | 1.40001E-09 | 0.00201649 | 458349 |
| rs35368205 | 7 | T | C | -0.0247085 | 0.197942 | 4.49987E-24 | 0.00249361 | 458349 |
| rs11770289 | 7 | A | C | -0.0295166 | 0.0890871 | 2.70023E-16 | 0.00357743 | 458349 |
| rs1601468 | 8 | A | G | 0.0163283 | 0.137846 | 6.69993E-09 | 0.00288354 | 458349 |
| rs622856 | 8 | T | C | -0.0171265 | 0.365713 | 3.29989E-17 | 0.00206389 | 458349 |
| rs4841132 | 8 | G | A | -0.019663 | 0.908757 | 3.29997E-08 | 0.0036732 | 458349 |
| rs6983481 | 8 | T | G | -0.0207036 | 0.240288 | 1E-18 | 0.00232763 | 458349 |
| rs11992444 | 8 | T | G | 0.0229863 | 0.509618 | 1.69981E-32 | 0.00199446 | 458349 |
| rs16919100 | 8 | C | T | 0.0150935 | 0.181497 | 4.60002E-09 | 0.00258042 | 458349 |
| rs4548227 | 8 | G | A | -0.0159537 | 0.697739 | 6.89922E-13 | 0.00216778 | 458349 |
| rs13702 | 8 | C | T | -0.0124155 | 0.287325 | 1.29999E-08 | 0.00220096 | 458349 |
| rs13250456 | 8 | T | C | 0.0444299 | 0.0726867 | 5.90065E-32 | 0.00383482 | 458349 |
| rs7832357 | 8 | G | A | 0.0159875 | 0.341841 | 9.30037E-14 | 0.00209863 | 458349 |
| rs10991437 | 9 | A | C | 0.0292357 | 0.116979 | 1.99986E-21 | 0.00309478 | 458349 |
| rs192111487 | 9 | T | C | -0.0483926 | 0.0150013 | 4.39997E-09 | 0.00832319 | 458349 |
| rs11793856 | 9 | G | C | -0.0143297 | 0.276099 | 5.40008E-11 | 0.00224106 | 458349 |
| rs111874795 | 9 | C | T | -0.0520967 | 0.0462565 | 3.90032E-29 | 0.00473322 | 458349 |
| rs7025811 | 9 | G | A | 0.0121849 | 0.364333 | 8.70001E-10 | 0.00206882 | 458349 |
| rs12554943 | 9 | C | T | -0.0181245 | 0.126616 | 1.2E-09 | 0.00299288 | 458349 |
| rs3808782 | 9 | T | C | -0.0201905 | 0.0849326 | 9.69996E-09 | 0.00357501 | 458349 |
| rs7031163 | 9 | T | C | 0.0238147 | 0.174831 | 1.39991E-19 | 0.00261921 | 458349 |
| rs4397735 | 10 | A | G | -0.0223287 | 0.116633 | 4.19952E-13 | 0.00310846 | 458349 |
| rs11198034 | 10 | T | C | -0.0154743 | 0.618503 | 4.49987E-14 | 0.00205022 | 458349 |
| rs7072380 | 10 | A | G | 0.0125276 | 0.507785 | 1.6E-10 | 0.0019899 | 458349 |
| rs1111875 | 10 | T | C | -0.0120053 | 0.408442 | 9.09997E-09 | 0.00202389 | 458349 |
| rs1757471 | 10 | T | C | 0.0147571 | 0.502325 | 1.29987E-13 | 0.0019891 | 458349 |
| rs79561207 | 10 | A | C | 0.0195649 | 0.0995156 | 1.5E-09 | 0.0033225 | 458349 |
| rs17597176 | 10 | T | C | 0.0202201 | 0.0827872 | 1.29999E-08 | 0.00361197 | 458349 |
| rs1044210 | 10 | T | G | -0.0129301 | 0.281687 | 1.2E-09 | 0.00221845 | 458349 |
| rs10821962 | 10 | G | A | -0.0122048 | 0.339866 | 6.1E-09 | 0.00210154 | 458349 |
| rs1749824 | 10 | A | C | -0.0175009 | 0.43267 | 3.80014E-19 | 0.00200774 | 458349 |
| rs10883756 | 10 | C | T | 0.012037 | 0.602786 | 1.6E-09 | 0.00203247 | 458349 |
| rs10787472 | 10 | C | A | 0.0188063 | 0.466076 | 2.39994E-21 | 0.00199353 | 458349 |
| rs7394746 | 11 | C | T | -0.020273 | 0.472689 | 2.70023E-25 | 0.00199258 | 458349 |
| rs62621409 | 11 | G | A | 0.0301874 | 0.0421073 | 1.6E-09 | 0.00500404 | 458349 |
| rs140201358 | 11 | G | C | 0.0921862 | 0.0133988 | 5.00035E-25 | 0.00887781 | 458349 |
| rs10840331 | 11 | C | T | 0.0144665 | 0.565104 | 1.10002E-13 | 0.00200741 | 458349 |
| rs56911719 | 11 | G | C | -0.0148304 | 0.200072 | 7.10003E-09 | 0.00248573 | 458349 |
| rs10832246 | 11 | A | G | 0.0139432 | 0.336778 | 9.70063E-11 | 0.00213411 | 458349 |
| rs34312154 | 11 | A | G | 0.0199406 | 0.10899 | 1.29999E-10 | 0.0031961 | 458349 |
| rs2509957 | 11 | A | C | 0.0183555 | 0.745015 | 2.09991E-16 | 0.0022765 | 458349 |
| rs7942991 | 11 | T | C | 0.0186143 | 0.301074 | 1.59993E-17 | 0.00216788 | 458349 |
| rs56271783 | 11 | C | G | 0.0627011 | 0.0447076 | 1.50003E-37 | 0.00488603 | 458349 |
| rs10891315 | 11 | G | A | -0.0242849 | 0.359408 | 8.80035E-32 | 0.00210158 | 458349 |
| rs718314 | 12 | G | A | 0.0342433 | 0.243766 | 1.80011E-50 | 0.00231256 | 458349 |
| rs11053221 | 12 | T | C | -0.0134227 | 0.39506 | 1.20005E-11 | 0.0020331 | 458349 |
| rs2695152 | 12 | G | A | 0.0132669 | 0.326961 | 8.30042E-11 | 0.00211794 | 458349 |
| rs10744167 | 12 | C | T | -0.0152539 | 0.433216 | 2.39994E-14 | 0.00200611 | 458349 |
| rs11147235 | 12 | C | T | -0.0164674 | 0.185493 | 1.6E-10 | 0.00255792 | 458349 |
| rs10878370 | 12 | G | T | 0.019183 | 0.279241 | 1.59993E-18 | 0.00221613 | 458349 |
| rs3764002 | 12 | T | C | -0.0171134 | 0.261007 | 4.19952E-14 | 0.00228065 | 458349 |
| rs1805741 | 12 | T | C | -0.0183328 | 0.710719 | 4.70002E-17 | 0.00221416 | 458349 |
| rs1443512 | 12 | C | A | -0.0333595 | 0.780353 | 3.29989E-44 | 0.00239769 | 458349 |
| rs653178 | 12 | T | C | -0.0120422 | 0.51617 | 6.19998E-10 | 0.00198735 | 458349 |
| rs2292137 | 12 | G | C | 0.0175496 | 0.354832 | 2.60016E-17 | 0.00214153 | 458349 |
| rs7133378 | 12 | A | G | -0.0420882 | 0.316567 | 3.19963E-86 | 0.00214566 | 458349 |
| rs9568486 | 13 | C | T | 0.0146511 | 0.154685 | 2.69998E-08 | 0.00275087 | 458349 |
| rs647978 | 13 | T | C | 0.0171922 | 0.867939 | 0.000000005 | 0.00294224 | 458349 |
| rs9521732 | 13 | A | C | -0.0153534 | 0.382701 | 9.70063E-15 | 0.00204963 | 458349 |
| rs797475 | 13 | G | A | 0.0401465 | 0.879733 | 4.00037E-39 | 0.00306362 | 458349 |
| rs61964261 | 13 | T | C | -0.0184642 | 0.12735 | 8.10009E-10 | 0.00298475 | 458349 |
| rs61747585 | 14 | A | C | 0.0309791 | 0.0416725 | 3.29997E-10 | 0.00500148 | 458349 |
| rs2526882 | 14 | A | G | 0.0119075 | 0.660536 | 3.29997E-09 | 0.0021214 | 458349 |
| rs6573198 | 14 | A | G | -0.0147833 | 0.717436 | 2.90001E-11 | 0.00221057 | 458349 |
| rs862049 | 14 | T | C | 0.0134715 | 0.655138 | 8.69961E-11 | 0.00209597 | 458349 |
| rs12435157 | 14 | A | G | 0.0218598 | 0.287183 | 8.69961E-24 | 0.00220775 | 458349 |
| rs35759537 | 14 | T | C | -0.0132051 | 0.194948 | 1.79999E-08 | 0.00251174 | 458349 |
| rs2414095 | 15 | G | A | -0.0150555 | 0.649309 | 2.70023E-14 | 0.00208663 | 458349 |
| rs2654980 | 15 | T | C | 0.0141039 | 0.24758 | 2.59998E-10 | 0.00230736 | 458349 |
| rs62002122 | 15 | T | C | 0.012723 | 0.334295 | 3.79997E-10 | 0.00216207 | 458349 |
| rs62011302 | 15 | A | G | 0.0165365 | 0.156384 | 8.50002E-10 | 0.00274179 | 458349 |
| rs750460 | 15 | A | G | -0.0124525 | 0.468577 | 5.19996E-10 | 0.00199196 | 458349 |
| rs12591294 | 15 | C | T | 0.0199979 | 0.216474 | 5.19996E-17 | 0.0024169 | 458349 |
| rs6603004 | 15 | G | A | 0.011174 | 0.521292 | 1.29999E-08 | 0.00199786 | 458349 |
| rs12185079 | 15 | T | C | 0.0133513 | 0.227336 | 0.000000032 | 0.00237869 | 458349 |
| rs10851545 | 15 | T | C | 0.0156847 | 0.672947 | 7.00003E-14 | 0.0021236 | 458349 |
| rs2289790 | 15 | C | T | -0.0161136 | 0.239165 | 1.59993E-12 | 0.00235361 | 458349 |
| rs3848370 | 16 | C | G | -0.0153385 | 0.234274 | 2.70023E-11 | 0.00235344 | 458349 |
| rs2047936 | 16 | G | A | -0.0156966 | 0.514222 | 5.00035E-16 | 0.00199216 | 458349 |
| rs16952251 | 16 | G | A | -0.0137196 | 0.314211 | 1.80011E-11 | 0.00214441 | 458349 |
| rs4786487 | 16 | C | G | -0.0225093 | 0.739882 | 2.49977E-23 | 0.00226992 | 458349 |
| rs8052655 | 16 | A | G | -0.031991 | 0.0439802 | 2.80027E-11 | 0.00486185 | 458349 |
| rs2925979 | 16 | C | T | -0.0265204 | 0.699973 | 8.60003E-35 | 0.00217493 | 458349 |
| rs7203193 | 16 | G | A | 0.0114562 | 0.486833 | 2.30001E-08 | 0.0019907 | 458349 |
| rs727428 | 17 | C | T | -0.0172193 | 0.556341 | 3.69999E-18 | 0.00200441 | 458349 |
| rs11078594 | 17 | C | G | -0.0149361 | 0.18333 | 0.000000016 | 0.00259864 | 458349 |
| rs12936587 | 17 | A | G | -0.0201722 | 0.47177 | 9.30037E-24 | 0.00199244 | 458349 |
| rs7207123 | 17 | A | G | -0.0150085 | 0.320683 | 5.50047E-12 | 0.00214847 | 458349 |
| rs8082188 | 17 | T | C | 0.0161219 | 0.307305 | 2.90001E-14 | 0.00215591 | 458349 |
| rs62062136 | 17 | C | T | 0.0239635 | 0.221126 | 3.40017E-23 | 0.00242015 | 458349 |
| rs227731 | 17 | G | T | 0.0105449 | 0.456584 | 4.20001E-08 | 0.00199729 | 458349 |
| rs1468566 | 17 | T | C | 0.0147177 | 0.421641 | 1.10002E-12 | 0.00201478 | 458349 |
| rs1605750 | 17 | A | G | -0.0224604 | 0.510724 | 3.59998E-31 | 0.00198758 | 458349 |
| rs11870577 | 17 | A | C | 0.0292257 | 0.0782333 | 1.10002E-15 | 0.00372545 | 458349 |
| rs35344256 | 17 | A | C | 0.0149167 | 0.305563 | 1.20005E-11 | 0.0021621 | 458349 |
| rs79958143 | 18 | A | G | 0.0267261 | 0.0691209 | 1.39991E-11 | 0.00393055 | 458349 |
| rs12454712 | 18 | C | T | -0.0170986 | 0.374927 | 1E-16 | 0.00206875 | 458349 |
| rs4800452 | 18 | T | C | 0.0193747 | 0.784894 | 1.99986E-15 | 0.0024233 | 458349 |
| rs11664106 | 18 | T | A | -0.0278115 | 0.369976 | 3.90032E-40 | 0.00210144 | 458349 |
| rs116307916 | 19 | T | C | -0.0342714 | 0.0350583 | 9.79941E-11 | 0.00551766 | 458349 |
| rs2864436 | 19 | C | T | 0.0126986 | 0.244804 | 0.000000017 | 0.00231714 | 458349 |
| rs3745348 | 19 | T | C | 0.0157646 | 0.29367 | 9.49948E-14 | 0.00218717 | 458349 |
| rs1075403 | 19 | G | T | -0.0261034 | 0.632387 | 7.70016E-39 | 0.00206666 | 458349 |
| rs429358 | 19 | C | T | -0.0241114 | 0.153523 | 1E-17 | 0.00277585 | 458349 |
| rs891088 | 19 | G | A | -0.0130079 | 0.262488 | 7.49998E-09 | 0.00226023 | 458349 |
| rs80076900 | 19 | T | C | 0.0282404 | 0.0498559 | 2.5E-10 | 0.00457732 | 458349 |
| rs10403360 | 19 | A | G | 0.0327555 | 0.422384 | 4.60045E-60 | 0.00201397 | 458349 |
| rs117597226 | 19 | A | C | -0.0319862 | 0.0303774 | 2.99999E-08 | 0.00580787 | 458349 |
| rs12986413 | 19 | T | A | -0.0149594 | 0.461099 | 2.49977E-13 | 0.0019976 | 458349 |
| rs805753 | 20 | C | T | 0.0152506 | 0.360617 | 1.20005E-13 | 0.00208051 | 458349 |
| rs2145272 | 20 | A | G | -0.0253478 | 0.635275 | 4.19952E-36 | 0.00207 | 458349 |
| rs8121509 | 20 | C | T | -0.014237 | 0.452486 | 9.49948E-13 | 0.00200241 | 458349 |
| rs6066103 | 20 | G | A | -0.0312715 | 0.318233 | 2.19989E-49 | 0.00212464 | 458349 |
| rs856411 | 20 | C | T | 0.0143136 | 0.642004 | 1E-11 | 0.00207544 | 458349 |
| rs143384 | 20 | G | A | -0.019219 | 0.405592 | 3.69999E-22 | 0.00204056 | 458349 |
| rs2000339 | 20 | A | G | -0.0199465 | 0.50535 | 7.89951E-23 | 0.00199434 | 458349 |
| rs867264 | 20 | A | G | 0.0138344 | 0.551994 | 7.29962E-12 | 0.00200225 | 458349 |
| rs2008843 | 21 | C | T | -0.0141667 | 0.218521 | 7.69999E-09 | 0.00241194 | 458349 |
| rs13052956 | 21 | A | G | -0.012614 | 0.485189 | 3.09999E-10 | 0.00204239 | 458349 |
| rs1016695 | 21 | C | A | -0.017473 | 0.386703 | 3.40017E-17 | 0.00205067 | 458349 |
| rs2294239 | 22 | G | A | -0.0247713 | 0.41232 | 4.30031E-32 | 0.00207313 | 458349 |
| rs4823086 | 22 | T | C | 0.0235228 | 0.126157 | 5.50047E-15 | 0.00302642 | 458349 |
| 5 SNPs are associated with Visceral adipose tissue volume | | | | | | | | |
| SNP | chr.exposure | effect_allele.exposure | other_allele.exposure | beta.exposure | eaf.exposure | pval.exposure | se.exposure | samplesize.exposure |
| rs157845 | 5 | C | T | -0.0488353 | 0.744688 | 9.49948E-11 | 0.00754263 | 32860 |
| rs73221948 | 8 | T | G | -0.0502029 | 0.293261 | 2.49977E-11 | 0.00752515 | 32860 |
| rs62048402 | 16 | A | G | 0.0374094 | 0.399483 | 2.99999E-08 | 0.00674831 | 32860 |
| rs11666808 | 19 | C | T | -0.0377322 | 0.627815 | 3.50002E-08 | 0.00684322 | 32860 |
| rs153701 | 19 | C | T | -0.0427213 | 0.547645 | 1.40001E-10 | 0.00665852 | 32860 |
| 257 SNPs are associated with body fat percentage | | | | | | | | |
| SNP | chr.exposure | effect_allele.exposure | other_allele.exposure | beta.exposure | eaf.exposure | pval.exposure | se.exposure | samplesize.exposure |
| rs11642015 | 16 | T | C | 0.0395226 | 0.40236 | 3.10313e-96 | 0.00189805 | 331117 |
| rs713586 | 2 | C | T | 0.0241683 | 0.489512 | 1.32069e-38 | 0.0018597 | 331117 |
| rs7124681 | 11 | A | C | 0.0234727 | 0.408057 | 2.27667e-35 | 0.00189106 | 331117 |
| rs62037365 | 16 | G | C | 0.0235124 | 0.402132 | 3.37365e-35 | 0.00189907 | 331117 |
| rs539515 | 1 | C | A | 0.0273917 | 0.207529 | 7.41481e-33 | 0.00229406 | 331117 |
| rs9358912 | 6 | T | G | -0.0242776 | 0.27022 | 6.16737e-31 | 0.00209893 | 331117 |
| rs62106258 | 2 | C | T | -0.0495541 | 0.0484669 | 3.10242e-30 | 0.00433655 | 331117 |
| rs10732335 | 9 | C | A | -0.0209884 | 0.443788 | 4.83838e-29 | 0.00187633 | 331117 |
| rs13135092 | 4 | G | A | 0.0362684 | 0.0832342 | 1.10205e-26 | 0.00339161 | 331117 |
| rs538656 | 18 | T | G | 0.0229814 | 0.234928 | 1.13475e-25 | 0.00219391 | 331117 |
| rs7649970 | 3 | T | C | 0.0298437 | 0.120866 | 1.18195e-25 | 0.00285006 | 331117 |
| rs3736896 | 6 | C | T | 0.0240561 | 0.204605 | 1.78033e-25 | 0.00230591 | 331117 |
| rs10938397 | 4 | G | A | 0.0194334 | 0.434075 | 5.58599e-25 | 0.0018825 | 331117 |
| rs2867131 | 2 | C | T | 0.025643 | 0.829971 | 6.04784e-25 | 0.00248587 | 331117 |
| rs1538742 | 1 | A | C | -0.0193895 | 0.569585 | 9.23422e-25 | 0.00188709 | 331117 |
| rs6717858 | 2 | C | T | 0.0194567 | 0.403398 | 1.31009e-24 | 0.00189989 | 331117 |
| rs61888762 | 11 | G | C | 0.020321 | 0.322009 | 1.92176e-24 | 0.0019915 | 331117 |
| rs80135947 | 17 | C | A | 0.0232969 | 0.195339 | 3.57602e-23 | 0.00234944 | 331117 |
| rs17296856 | 15 | C | A | -0.0204763 | 0.281961 | 5.12625e-23 | 0.00207252 | 331117 |
| rs12367809 | 12 | T | C | 0.0189962 | 0.368275 | 1.13214e-22 | 0.00193836 | 331117 |
| rs9843653 | 3 | C | T | 0.0181875 | 0.514555 | 1.66418e-22 | 0.00186325 | 331117 |
| rs55714539 | 19 | C | A | 0.0190841 | 0.342691 | 4.19662e-22 | 0.00197417 | 331117 |
| rs10423928 | 19 | A | T | -0.0225756 | 0.19349 | 7.46621e-22 | 0.00234974 | 331117 |
| rs7133378 | 12 | A | G | 0.0191054 | 0.317373 | 1.43747e-21 | 0.00200266 | 331117 |
| rs1928185 | 6 | C | T | 0.0231796 | 0.168441 | 1.35332e-20 | 0.00249121 | 331117 |
| rs4790841 | 17 | T | C | -0.023527 | 0.154867 | 7.52489e-20 | 0.0025796 | 331117 |
| rs9320823 | 6 | C | T | 0.0173426 | 0.601901 | 8.8044e-20 | 0.00190508 | 331117 |
| rs11012732 | 10 | G | A | 0.017988 | 0.331106 | 1.03848e-19 | 0.00197988 | 331117 |
| rs28483178 | 19 | G | C | 0.0182802 | 0.303189 | 2.36102e-19 | 0.00203212 | 331117 |
| rs67807996 | 1 | A | G | 0.0175719 | 0.401994 | 3.34041e-19 | 0.00196171 | 331117 |
| rs2112347 | 5 | G | T | -0.0172924 | 0.359214 | 6.06876e-19 | 0.00194487 | 331117 |
| rs11866219 | 16 | C | A | -0.0169461 | 0.583662 | 7.4114e-19 | 0.0019107 | 331117 |
| rs16951304 | 15 | C | T | -0.020299 | 0.206692 | 9.49074e-19 | 0.00229589 | 331117 |
| rs1460940 | 1 | A | G | 0.02037 | 0.801745 | 2.8132e-18 | 0.00233621 | 331117 |
| rs2370982 | 14 | T | C | 0.0198907 | 0.215336 | 2.95121e-18 | 0.00228266 | 331117 |
| rs34483452 | 5 | A | C | 0.0237918 | 0.135156 | 4.54046e-18 | 0.00274578 | 331117 |
| rs879620 | 16 | T | C | 0.0165566 | 0.615276 | 6.79673e-18 | 0.00192099 | 331117 |
| rs35060985 | 11 | A | G | 0.0172488 | 0.316828 | 7.83971e-18 | 0.00200511 | 331117 |
| rs57866767 | 10 | C | T | -0.0161613 | 0.432354 | 8.23569e-18 | 0.00187993 | 331117 |
| rs4821764 | 22 | A | G | -0.0162016 | 0.581428 | 9.4189e-18 | 0.00188801 | 331117 |
| rs4889606 | 16 | G | A | -0.0162391 | 0.376469 | 3.65342e-17 | 0.00192772 | 331117 |
| rs4876611 | 8 | G | A | 0.0173632 | 0.715809 | 4.3401e-17 | 0.00206612 | 331117 |
| rs1454687 | 3 | G | C | -0.0155182 | 0.515055 | 7.97627e-17 | 0.00186248 | 331117 |
| rs12679106 | 8 | T | G | -0.0171708 | 0.711341 | 9.32825e-17 | 0.00206542 | 331117 |
| rs3764002 | 12 | T | C | -0.0175211 | 0.261039 | 1.36144e-16 | 0.00211902 | 331117 |
| rs2855818 | 17 | A | G | 0.0180974 | 0.236531 | 2.21157e-16 | 0.00220419 | 331117 |
| rs35882248 | 2 | T | C | 0.0163971 | 0.314642 | 3.1232e-16 | 0.00200726 | 331117 |
| rs2678204 | 1 | G | T | 0.0160146 | 0.341813 | 3.67621e-16 | 0.00196518 | 331117 |
| rs1893659 | 18 | A | C | -0.0152601 | 0.463084 | 4.42079e-16 | 0.00187773 | 331117 |
| rs6545714 | 2 | A | G | -0.0152536 | 0.601551 | 1.14578e-15 | 0.00190419 | 331117 |
| rs10999460 | 10 | T | C | 0.016798 | 0.265951 | 2.01002e-15 | 0.00211532 | 331117 |
| rs10756798 | 9 | T | C | -0.0153953 | 0.645026 | 2.64545e-15 | 0.00194705 | 331117 |
| rs17770336 | 9 | T | C | 0.0156916 | 0.323821 | 2.81384e-15 | 0.00198646 | 331117 |
| rs34045288 | 6 | T | C | 0.0155172 | 0.335903 | 3.68893e-15 | 0.00197283 | 331117 |
| rs704061 | 12 | C | T | 0.0146934 | 0.452703 | 4.09921e-15 | 0.00187124 | 331117 |
| rs3817428 | 15 | G | C | -0.016498 | 0.267231 | 4.27957e-15 | 0.0021025 | 331117 |
| rs952227 | 2 | G | A | -0.0155751 | 0.673163 | 4.42283e-15 | 0.00198593 | 331117 |
| rs57636386 | 18 | C | T | -0.0262411 | 0.0834151 | 7.33838e-15 | 0.00337338 | 331117 |
| rs972283 | 7 | G | A | -0.0144028 | 0.509677 | 1.03562e-14 | 0.00186199 | 331117 |
| rs17024393 | 1 | C | T | 0.0454815 | 0.0257993 | 1.17382e-14 | 0.00589198 | 331117 |
| rs10100245 | 8 | A | G | 0.0143788 | 0.56644 | 1.93286e-14 | 0.00187827 | 331117 |
| rs7972728 | 12 | A | C | 0.016177 | 0.73741 | 2.26204e-14 | 0.00211875 | 331117 |
| rs62261725 | 3 | G | A | -0.0151469 | 0.327437 | 2.49115e-14 | 0.00198708 | 331117 |
| rs72976986 | 19 | A | G | -0.0181336 | 0.191187 | 3.53346e-14 | 0.0023931 | 331117 |
| rs241459 | 1 | C | A | -0.0150756 | 0.680281 | 3.85922e-14 | 0.00199255 | 331117 |
| rs2052607 | 18 | A | G | -0.0148542 | 0.344774 | 3.94821e-14 | 0.00196406 | 331117 |
| rs12339822 | 9 | G | A | 0.0140943 | 0.5432 | 5.97035e-14 | 0.00187698 | 331117 |
| rs4671328 | 2 | G | T | -0.0141033 | 0.552555 | 7.8759e-14 | 0.00188732 | 331117 |
| rs2914231 | 5 | C | G | -0.0165342 | 0.234279 | 8.30233e-14 | 0.00221467 | 331117 |
| rs73041988 | 11 | G | T | -0.0186581 | 0.16616 | 9.13692e-14 | 0.0025034 | 331117 |
| rs2178899 | 6 | T | A | -0.0206057 | 0.129898 | 9.54553e-14 | 0.00276685 | 331117 |
| rs479018 | 11 | A | G | -0.0149054 | 0.333201 | 1.15372e-13 | 0.00200819 | 331117 |
| rs2038646 | 1 | G | C | 0.0140692 | 0.598633 | 1.24022e-13 | 0.00189798 | 331117 |
| rs2192527 | 4 | G | A | 0.0137966 | 0.464952 | 1.6592e-13 | 0.00187097 | 331117 |
| rs58862095 | 7 | T | C | -0.0139264 | 0.419757 | 1.79721e-13 | 0.00189131 | 331117 |
| rs1013293 | 1 | A | G | -0.0137428 | 0.433339 | 3.23594e-13 | 0.00188658 | 331117 |
| rs7139583 | 13 | C | A | -0.0148362 | 0.289937 | 4.70977e-13 | 0.00205098 | 331117 |
| rs11474838 | 20 | G | T | 0.0138628 | 0.432734 | 4.70977e-13 | 0.00191642 | 331117 |
| rs76929617 | 12 | G | A | -0.0348085 | 0.0388598 | 5.18561e-13 | 0.00482068 | 331117 |
| rs9522279 | 13 | T | C | 0.0136103 | 0.425038 | 5.57699e-13 | 0.0018875 | 331117 |
| rs2415142 | 15 | G | T | 0.0134616 | 0.549902 | 6.8281e-13 | 0.00187405 | 331117 |
| rs987469 | 4 | G | C | -0.0134445 | 0.460673 | 6.9775e-13 | 0.00187245 | 331117 |
| rs76040172 | 21 | A | G | -0.0295385 | 0.0544397 | 7.13346e-13 | 0.00411564 | 331117 |
| rs6575340 | 14 | A | G | 0.0138061 | 0.637416 | 1.13397e-12 | 0.00194084 | 331117 |
| rs7613261 | 3 | T | A | 0.0163508 | 0.204563 | 1.54348e-12 | 0.00231244 | 331117 |
| rs245775 | 5 | G | A | 0.0147507 | 0.728826 | 1.96698e-12 | 0.00209615 | 331117 |
| rs113230003 | 19 | A | G | -0.0150436 | 0.260185 | 2.00493e-12 | 0.00213857 | 331117 |
| rs7707394 | 5 | A | G | -0.0135868 | 0.354316 | 2.70334e-12 | 0.00194302 | 331117 |
| rs10848835 | 12 | C | G | -0.0219468 | 0.0972438 | 3.08603e-12 | 0.00314694 | 331117 |
| rs111743285 | 6 | T | C | 0.0156168 | 0.222481 | 3.3243e-12 | 0.00224266 | 331117 |
| rs7982447 | 13 | C | T | 0.0160514 | 0.206408 | 3.51884e-12 | 0.00230771 | 331117 |
| rs429358 | 19 | C | T | -0.0178118 | 0.156331 | 3.73508e-12 | 0.00256392 | 331117 |
| rs12049202 | 1 | T | C | 0.0161982 | 0.198651 | 3.77746e-12 | 0.00233218 | 331117 |
| rs4718964 | 7 | T | G | 0.0131514 | 0.412194 | 4.06818e-12 | 0.00189636 | 331117 |
| rs6861649 | 5 | C | T | 0.0132352 | 0.607485 | 4.65908e-12 | 0.00191375 | 331117 |
| rs11943456 | 4 | C | T | 0.0128758 | 0.46153 | 5.73588e-12 | 0.00186977 | 331117 |
| rs10883026 | 10 | T | C | -0.0129056 | 0.520437 | 6.04923e-12 | 0.00187616 | 331117 |
| rs10259620 | 7 | G | A | -0.0157427 | 0.787171 | 6.2431e-12 | 0.0022901 | 331117 |
| rs809955 | 4 | A | G | -0.0132408 | 0.366632 | 7.66126e-12 | 0.00193437 | 331117 |
| rs3803286 | 14 | G | A | -0.0134756 | 0.666379 | 8.4217e-12 | 0.0019726 | 331117 |
| rs1056441 | 20 | C | T | 0.0135525 | 0.673163 | 8.69161e-12 | 0.00198516 | 331117 |
| rs2003476 | 19 | C | T | -0.0128971 | 0.407079 | 1.23282e-11 | 0.0019032 | 331117 |
| rs2172131 | 10 | C | T | -0.012733 | 0.580636 | 1.523e-11 | 0.00188751 | 331117 |
| rs4549685 | 7 | T | C | -0.0133503 | 0.330375 | 1.57109e-11 | 0.00198033 | 331117 |
| rs10209821 | 2 | T | C | 0.0131612 | 0.341092 | 2.04739e-11 | 0.00196353 | 331117 |
| rs2239647 | 14 | C | A | -0.0125561 | 0.551791 | 2.05353e-11 | 0.00187337 | 331117 |
| rs6847975 | 4 | A | G | 0.0130709 | 0.356005 | 2.24543e-11 | 0.00195399 | 331117 |
| rs11079849 | 17 | T | C | -0.0132846 | 0.327359 | 2.37848e-11 | 0.00198844 | 331117 |
| rs13062093 | 3 | G | T | 0.0128602 | 0.366444 | 2.73653e-11 | 0.00193087 | 331117 |
| rs10954772 | 8 | C | T | -0.0134343 | 0.685712 | 2.81709e-11 | 0.00201836 | 331117 |
| rs2306593 | 17 | T | C | -0.0124024 | 0.488522 | 2.98126e-11 | 0.00186566 | 331117 |
| rs525101 | 13 | C | T | 0.0128139 | 0.371963 | 3.16446e-11 | 0.00193011 | 331117 |
| rs2124499 | 3 | C | G | -0.0127943 | 0.368767 | 3.53753e-11 | 0.00193195 | 331117 |
| rs40071 | 5 | C | T | -0.0160885 | 0.179293 | 3.74024e-11 | 0.0024324 | 331117 |
| rs11042030 | 11 | C | T | -0.0137824 | 0.274009 | 4.24424e-11 | 0.00208966 | 331117 |
| rs1446585 | 2 | G | A | -0.014693 | 0.228006 | 4.37925e-11 | 0.00222929 | 331117 |
| rs1441264 | 13 | A | G | 0.0127196 | 0.591866 | 4.90569e-11 | 0.00193484 | 331117 |
| rs1503526 | 5 | C | T | 0.012187 | 0.479184 | 6.19441e-11 | 0.00186369 | 331117 |
| rs61975142 | 14 | A | G | -0.0163082 | 0.167226 | 6.34162e-11 | 0.00249525 | 331117 |
| rs2042864 | 3 | C | T | 0.0124345 | 0.404211 | 6.64967e-11 | 0.00190463 | 331117 |
| rs56226325 | 7 | T | C | -0.0168241 | 0.153377 | 7.50067e-11 | 0.00258415 | 331117 |
| rs1927626 | 9 | T | C | 0.0248644 | 0.936314 | 7.51104e-11 | 0.00381925 | 331117 |
| rs2111281 | 12 | C | A | 0.0125795 | 0.36592 | 8.11335e-11 | 0.0019357 | 331117 |
| rs3766823 | 1 | A | G | 0.0160455 | 0.171635 | 8.17147e-11 | 0.00246944 | 331117 |
| rs12622267 | 2 | G | A | -0.0125742 | 0.3672 | 9.3519e-11 | 0.00194128 | 331117 |
| rs12103006 | 16 | G | A | 0.012178 | 0.570767 | 1.03162e-10 | 0.00188443 | 331117 |
| rs10886022 | 10 | C | A | -0.0144338 | 0.766266 | 1.08096e-10 | 0.00223594 | 331117 |
| rs2436772 | 2 | A | G | -0.0147766 | 0.208771 | 1.10634e-10 | 0.00229029 | 331117 |
| rs2371767 | 3 | C | G | 0.0134874 | 0.273109 | 1.14377e-10 | 0.0020921 | 331117 |
| rs11150745 | 17 | G | A | -0.0128721 | 0.319489 | 1.21571e-10 | 0.00199954 | 331117 |
| rs7442885 | 5 | G | C | -0.0147198 | 0.210104 | 1.32483e-10 | 0.0022912 | 331117 |
| rs12619178 | 2 | T | C | -0.0121832 | 0.402922 | 1.37158e-10 | 0.00189793 | 331117 |
| rs10499014 | 6 | G | C | -0.0135376 | 0.26787 | 1.60987e-10 | 0.00211698 | 331117 |
| rs4284389 | 11 | T | C | 0.011868 | 0.499012 | 1.82995e-10 | 0.0018616 | 331117 |
| rs4547132 | 11 | T | C | -0.01337 | 0.266593 | 2.08752e-10 | 0.00210387 | 331117 |
| rs2043016 | 2 | T | C | 0.0122624 | 0.375877 | 2.10615e-10 | 0.00193 | 331117 |
| rs10754132 | 1 | T | A | -0.0121964 | 0.376496 | 2.13074e-10 | 0.00192014 | 331117 |
| rs2861685 | 2 | C | T | -0.0119956 | 0.411371 | 2.18077e-10 | 0.00188959 | 331117 |
| rs301806 | 1 | T | C | 0.0119918 | 0.584225 | 2.19543e-10 | 0.0018893 | 331117 |
| rs34417222 | 20 | T | C | -0.0127871 | 0.310458 | 2.23218e-10 | 0.00201541 | 331117 |
| rs114712833 | 3 | T | C | -0.0236749 | 0.0671295 | 2.25819e-10 | 0.00373253 | 331117 |
| rs10172196 | 2 | A | G | 0.0128454 | 0.304839 | 2.44833e-10 | 0.00202917 | 331117 |
| rs12037698 | 1 | A | G | -0.0168353 | 0.141878 | 2.86418e-10 | 0.00266966 | 331117 |
| rs79113395 | 1 | A | G | -0.0133428 | 0.265449 | 2.97016e-10 | 0.00211773 | 331117 |
| rs10766077 | 11 | A | G | -0.0122742 | 0.365106 | 2.99027e-10 | 0.00194845 | 331117 |
| rs1928496 | 13 | T | C | 0.0133849 | 0.742388 | 3.10599e-10 | 0.00212675 | 331117 |
| rs1724557 | 4 | A | C | -0.0119372 | 0.58831 | 3.37031e-10 | 0.00190055 | 331117 |
| rs11786089 | 8 | G | A | 0.0117578 | 0.456852 | 3.42153e-10 | 0.00187269 | 331117 |
| rs4759318 | 12 | T | C | 0.012171 | 0.3622 | 3.52387e-10 | 0.00193993 | 331117 |
| rs9902386 | 17 | G | T | -0.0125968 | 0.677151 | 3.72881e-10 | 0.00201061 | 331117 |
| rs11782341 | 8 | G | A | 0.0149442 | 0.188513 | 4.57552e-10 | 0.00239752 | 331117 |
| rs55637757 | 16 | T | C | -0.0173246 | 0.133438 | 4.8356e-10 | 0.00278328 | 331117 |
| rs2606228 | 3 | C | A | -0.012235 | 0.642769 | 4.87326e-10 | 0.00196599 | 331117 |
| rs2002023 | 10 | T | C | 0.0117781 | 0.407707 | 5.41527e-10 | 0.00189763 | 331117 |
| rs8087074 | 18 | T | G | 0.013184 | 0.261119 | 5.99819e-10 | 0.00212967 | 331117 |
| rs12890931 | 14 | G | T | 0.0120472 | 0.363463 | 6.14427e-10 | 0.00194722 | 331117 |
| rs56803094 | 15 | G | A | -0.0137721 | 0.227111 | 6.31801e-10 | 0.00222761 | 331117 |
| rs815611 | 5 | A | G | -0.0115299 | 0.552303 | 7.14431e-10 | 0.00187082 | 331117 |
| rs10887578 | 10 | C | G | 0.0115263 | 0.502263 | 7.41583e-10 | 0.00187202 | 331117 |
| rs6927268 | 6 | G | T | -0.014181 | 0.204921 | 7.90934e-10 | 0.00230701 | 331117 |
| rs7925100 | 11 | A | G | 0.0116743 | 0.396337 | 8.61668e-10 | 0.00190342 | 331117 |
| rs75641275 | 1 | C | A | 0.0162428 | 0.143946 | 9.20322e-10 | 0.00265283 | 331117 |
| rs7933085 | 11 | G | A | 0.0114446 | 0.509195 | 9.71561e-10 | 0.00187181 | 331117 |
| rs1568489 | 3 | G | A | 0.0115308 | 0.574397 | 1.01676e-09 | 0.00188814 | 331117 |
| rs12724928 | 1 | C | T | -0.0140614 | 0.205376 | 1.01735e-09 | 0.00230256 | 331117 |
| rs9551991 | 13 | C | T | 0.0162053 | 0.143839 | 1.01979e-09 | 0.00265379 | 331117 |
| rs2281819 | 6 | A | T | -0.0135027 | 0.2294 | 1.15112e-09 | 0.00221825 | 331117 |
| rs10278040 | 7 | A | G | -0.0236879 | 0.0606882 | 1.30614e-09 | 0.0039045 | 331117 |
| rs117068593 | 14 | T | C | -0.0144504 | 0.189341 | 1.35859e-09 | 0.00238437 | 331117 |
| rs62414900 | 6 | T | C | -0.0256209 | 0.0516134 | 1.35997e-09 | 0.00422764 | 331117 |
| rs10947793 | 6 | G | A | -0.011725 | 0.373604 | 1.3781e-09 | 0.00193539 | 331117 |
| rs7591494 | 2 | A | G | 0.0128643 | 0.261436 | 1.46998e-09 | 0.00212711 | 331117 |
| rs2318543 | 4 | G | A | -0.0136603 | 0.782495 | 1.62387e-09 | 0.00226473 | 331117 |
| rs17265513 | 20 | C | T | 0.013987 | 0.199974 | 1.85477e-09 | 0.0023272 | 331117 |
| rs215614 | 7 | A | G | -0.0115735 | 0.628329 | 1.99379e-09 | 0.00192941 | 331117 |
| rs11856579 | 15 | A | G | -0.0125813 | 0.268192 | 2.13875e-09 | 0.00210141 | 331117 |
| rs72995085 | 6 | C | T | -0.0146267 | 0.177337 | 2.23501e-09 | 0.00244598 | 331117 |
| rs2660241 | 16 | C | T | 0.0116064 | 0.36282 | 2.23651e-09 | 0.00194094 | 331117 |
| rs59066241 | 12 | G | T | 0.0172863 | 0.118268 | 2.41074e-09 | 0.0028967 | 331117 |
| rs1701820 | 7 | A | G | -0.0111783 | 0.550656 | 2.48617e-09 | 0.00187476 | 331117 |
| rs1218822 | 13 | A | G | 0.0117383 | 0.662041 | 2.49833e-09 | 0.00196894 | 331117 |
| rs13109830 | 4 | G | A | -0.0111276 | 0.458762 | 2.60423e-09 | 0.00186863 | 331117 |
| rs4690324 | 4 | A | G | -0.0145244 | 0.821582 | 2.64113e-09 | 0.00243999 | 331117 |
| rs7630228 | 3 | C | T | -0.0112417 | 0.435046 | 2.64996e-09 | 0.0018887 | 331117 |
| rs2984618 | 1 | T | G | 0.0112583 | 0.416855 | 2.70377e-09 | 0.00189253 | 331117 |
| rs13174863 | 5 | G | A | 0.0156688 | 0.148208 | 2.79776e-09 | 0.00263643 | 331117 |
| rs28726372 | 1 | C | T | 0.0119642 | 0.307814 | 3.04145e-09 | 0.00201774 | 331117 |
| rs11852419 | 15 | T | A | 0.0125094 | 0.261831 | 3.45096e-09 | 0.00211711 | 331117 |
| rs56399737 | 13 | T | C | -0.0110847 | 0.448781 | 3.46362e-09 | 0.00187618 | 331117 |
| rs78801969 | 16 | T | C | -0.0157082 | 0.142231 | 3.66463e-09 | 0.00266294 | 331117 |
| rs12477385 | 2 | T | G | -0.013105 | 0.2273 | 4.11434e-09 | 0.00222887 | 331117 |
| rs56203712 | 4 | G | A | -0.0131536 | 0.238079 | 4.23175e-09 | 0.00223891 | 331117 |
| rs217672 | 14 | C | A | 0.0123142 | 0.272013 | 4.29744e-09 | 0.00209693 | 331117 |
| rs6888037 | 5 | T | G | 0.0126699 | 0.751706 | 4.70934e-09 | 0.00216309 | 331117 |
| rs2954033 | 8 | G | A | 0.0118546 | 0.697459 | 4.84128e-09 | 0.00202549 | 331117 |
| rs11208779 | 1 | C | G | 0.0109491 | 0.531674 | 4.89362e-09 | 0.00187135 | 331117 |
| rs12926311 | 16 | C | G | -0.0113803 | 0.355425 | 5.46085e-09 | 0.00195115 | 331117 |
| rs4503172 | 9 | T | C | -0.0111218 | 0.607824 | 5.6295e-09 | 0.00190848 | 331117 |
| rs4450871 | 4 | G | A | 0.0109096 | 0.4414 | 5.7277e-09 | 0.001873 | 331117 |
| rs73035223 | 7 | G | A | 0.015286 | 0.148535 | 5.75811e-09 | 0.00262476 | 331117 |
| rs75854315 | 1 | A | G | 0.0219425 | 0.0656378 | 5.99474e-09 | 0.0037721 | 331117 |
| rs11873650 | 18 | G | A | 0.0118891 | 0.297262 | 6.186e-09 | 0.00204568 | 331117 |
| rs6925197 | 6 | T | C | -0.0132234 | 0.213776 | 6.94624e-09 | 0.0022829 | 331117 |
| rs6948959 | 7 | A | G | -0.0124028 | 0.743925 | 7.30668e-09 | 0.00214437 | 331117 |
| rs2398861 | 9 | G | A | 0.0123803 | 0.256599 | 7.31223e-09 | 0.00214054 | 331117 |
| rs62285233 | 3 | A | G | -0.0180601 | 0.102336 | 7.34649e-09 | 0.00312299 | 331117 |
| rs11742930 | 5 | T | C | 0.0108693 | 0.566428 | 7.74926e-09 | 0.00188246 | 331117 |
| rs4864201 | 4 | C | T | -0.0112519 | 0.6533 | 8.55697e-09 | 0.00195439 | 331117 |
| rs7498044 | 15 | A | G | -0.0130365 | 0.218661 | 9.66563e-09 | 0.0022725 | 331117 |
| rs11043298 | 12 | A | T | -0.0148867 | 0.177685 | 9.75124e-09 | 0.00259569 | 331117 |
| rs117632017 | 15 | A | G | 0.0286098 | 0.0391416 | 1.03595e-08 | 0.00499744 | 331117 |
| rs11245344 | 10 | T | C | 0.010755 | 0.570426 | 1.13608e-08 | 0.0018838 | 331117 |
| rs2702123 | 2 | C | T | -0.0190216 | 0.0853962 | 1.15545e-08 | 0.00333343 | 331117 |
| rs8042404 | 15 | A | G | 0.0119627 | 0.270723 | 1.17298e-08 | 0.00209733 | 331117 |
| rs59104534 | 8 | T | C | 0.0115801 | 0.301112 | 1.32651e-08 | 0.00203777 | 331117 |
| rs12475388 | 2 | A | G | -0.0106367 | 0.486748 | 1.32776e-08 | 0.0018718 | 331117 |
| rs58360798 | 2 | C | T | 0.0134742 | 0.191016 | 1.34382e-08 | 0.002372 | 331117 |
| rs6875585 | 5 | C | A | 0.0112473 | 0.669998 | 1.47397e-08 | 0.00198551 | 331117 |
| rs7238896 | 18 | G | A | 0.0152365 | 0.140717 | 1.51911e-08 | 0.00269219 | 331117 |
| rs6973656 | 7 | G | A | 0.0107456 | 0.396582 | 1.57569e-08 | 0.00190078 | 331117 |
| rs79478789 | 21 | C | A | 0.0144098 | 0.159216 | 1.58862e-08 | 0.00254958 | 331117 |
| rs13062660 | 3 | C | T | 0.0110786 | 0.338985 | 1.68202e-08 | 0.00196359 | 331117 |
| rs815163 | 1 | C | T | -0.0105722 | 0.561603 | 1.736e-08 | 0.00187564 | 331117 |
| rs10774018 | 12 | C | G | 0.0127358 | 0.217907 | 1.80597e-08 | 0.00226222 | 331117 |
| rs7259070 | 19 | C | T | 0.0107802 | 0.599006 | 1.88322e-08 | 0.00191731 | 331117 |
| rs11030016 | 11 | T | C | 0.011958 | 0.740646 | 1.92695e-08 | 0.0021283 | 331117 |
| rs72917533 | 2 | C | T | -0.0134582 | 0.186273 | 1.99793e-08 | 0.00239797 | 331117 |
| rs7774 | 17 | A | C | 0.011376 | 0.309138 | 2.01688e-08 | 0.00202757 | 331117 |
| rs10830566 | 11 | G | A | -0.0116587 | 0.278192 | 2.04357e-08 | 0.00207879 | 331117 |
| rs73213484 | 4 | T | A | -0.0150818 | 0.139056 | 2.12178e-08 | 0.00269226 | 331117 |
| rs2487585 | 1 | G | A | 0.0121015 | 0.754597 | 2.16371e-08 | 0.00216155 | 331117 |
| rs1881505 | 11 | C | T | -0.0225144 | 0.942104 | 2.22285e-08 | 0.00402486 | 331117 |
| rs75846784 | 5 | G | T | -0.0197217 | 0.0785931 | 2.22469e-08 | 0.00352571 | 331117 |
| rs1783541 | 11 | T | C | 0.0126056 | 0.218469 | 2.28686e-08 | 0.00225547 | 331117 |
| rs4547574 | 2 | T | A | -0.0124569 | 0.225874 | 2.40315e-08 | 0.0022323 | 331117 |
| rs7094644 | 10 | A | G | 0.0112809 | 0.674176 | 2.4632e-08 | 0.00202312 | 331117 |
| rs114295766 | 3 | T | A | -0.0216475 | 0.0697799 | 2.67713e-08 | 0.0038924 | 331117 |
| rs62072006 | 17 | C | A | 0.0147373 | 0.145009 | 2.74998e-08 | 0.00265212 | 331117 |
| rs6792984 | 3 | T | C | 0.0112212 | 0.308285 | 2.75353e-08 | 0.00201945 | 331117 |
| rs12583872 | 13 | C | A | -0.0111762 | 0.309733 | 2.82983e-08 | 0.00201309 | 331117 |
| rs10854853 | 22 | T | G | 0.0103417 | 0.458065 | 3.01495e-08 | 0.0018665 | 331117 |
| rs4722398 | 7 | T | C | 0.0150172 | 0.135663 | 3.0297e-08 | 0.00271076 | 331117 |
| rs16934748 | 10 | C | T | 0.0143728 | 0.151594 | 3.10864e-08 | 0.00259655 | 331117 |
| rs4752183 | 10 | G | T | -0.0104468 | 0.571726 | 3.10864e-08 | 0.00188729 | 331117 |
| rs1486921 | 8 | T | C | -0.0112795 | 0.297004 | 3.16796e-08 | 0.00203894 | 331117 |
| rs11790018 | 9 | G | C | -0.0107118 | 0.36865 | 3.22151e-08 | 0.00193735 | 331117 |
| rs7570258 | 2 | C | T | 0.0102828 | 0.509268 | 3.3021e-08 | 0.00186123 | 331117 |
| rs9803921 | 1 | A | C | -0.0105787 | 0.382249 | 3.43321e-08 | 0.00191716 | 331117 |
| rs2499468 | 6 | A | C | 0.0108029 | 0.652736 | 3.49148e-08 | 0.00195884 | 331117 |
| rs72820274 | 2 | A | G | 0.0104249 | 0.41876 | 3.49695e-08 | 0.0018904 | 331117 |
| rs11685627 | 2 | A | G | -0.0103882 | 0.48454 | 3.50131e-08 | 0.00188382 | 331117 |
| rs4709745 | 6 | C | T | 0.0111427 | 0.305441 | 3.52826e-08 | 0.00202113 | 331117 |
| rs878206 | 14 | G | A | 0.0102685 | 0.542473 | 3.72237e-08 | 0.00186575 | 331117 |
| rs6977416 | 7 | A | G | -0.0109725 | 0.331226 | 3.82103e-08 | 0.00199535 | 331117 |
| rs136308 | 22 | T | C | 0.0127056 | 0.204381 | 3.88419e-08 | 0.00231171 | 331117 |
| rs17704028 | 7 | T | C | -0.0144201 | 0.147133 | 4.29062e-08 | 0.00263209 | 331117 |
| rs79535757 | 12 | T | A | -0.0574879 | 0.00819546 | 4.96707e-08 | 0.0105432 | 331117 |

**Supplementary Table 4. Sensitivity analysis results of the correlation between BMI, %BF, WC, WHR, and cognitive impairment**

|  | Heterogeneity test | | MR-Egger intercept test | |
| --- | --- | --- | --- | --- |
| Exposure vs. Outcome | Cochran’sQ | p-Value | Intercept | p-Value |
| BMI vs. Cognitive performance | 2239.943193 | 1.73E-224 | -0.000503039 | 0.53466617 |
| BMI vs. Cognitive function | 486.0459389 | 8.73E-02 | -0.001471252 | 4.19E-01 |
| BMI vs. AD | 2896.243349 | 0.00E+00 | 0.007808931 | 1.53E-01 |
| BMI vs. Dementia | 1819.404824 | 2.63E-155 | 0.001079287 | 8.27E-01 |
| %BF vs. Cognitive performance | 1400.230648 | 9.66E-169 | -0.002851181 | 1.43E-01 |
| %BF vs. Cognitive function | 234.9236234 | 2.48E-01 | -0.004596912 | 2.11E-01 |
| %BF vs. AD | 2719.031795 | 0.00E+00 | 0.009089138 | 5.57E-01 |
| %BF vs. Dementia | 247.9896104 | 3.99E-01 | -0.005547046 | 2.79E-01 |
| WC vs. Cognitive performance | 1414.471231 | 2.93E-158 | -0.001668164 | 1.96E-01 |
| WC vs. Cognitive function MCI | 278.6924728 | 1.38E-01 | -0.001461282 | 5.91E-01 |
| WC vs. AD | 2738.308756 | 0.00E+00 | 0.010032765 | 3.42E-01 |
| WC vs. Dementia | 265.7230672 | 5.45E-01 | 0.000168826 | 9.64E-01 |
| WHR vs. Cognitive performance | 758.3268988 | 1.27E-56 | -0.001881215 | 5.29E-02 |
| WHR vs. Cognitive function MCI | 217.6434595 | 4.37E-01 | -0.003518066 | 1.89E-01 |
| WHR vs. AD | 2733.733965 | 0.00E+00 | 0.00251321 | 8.42E-01 |
| WHR vs. Dementia | 256.1443131 | 1.42E-01 | 0.004766975 | 2.27E-01 |
| VAT vs. Cognitive performance CI | 21.21965071 | 2.86E-04 | 0.000428243 | 9.89E-01 |
| VAT vs. Cognitive function MCI | 2.389935469 | 4.96E-01 | -0.033339998 | 6.60E-01 |
| VAT vs. AD | 0.332100401 | 8.47E-01 | 0.053907309 | 6.77E-01 |
| VAT vs. Dementia | 2.693789484 | 6.10E-01 | 0.006085975 | 9.40E-01 |

Note: BMI, body mass index; WC, waist circumference; WHR, waist-to-hip ratio; %BF, Percentage of body fat; VAT, Visceral adipose tissue; CI, cognitive impairment; MCI, mild cognitive impairment; AD, Alzheimer's disease.

Supplementary Table 5. Details of GWAS data on exposure and outcome

| Type | GWAS ID | Data sources | Sample size | Ancestry |
| --- | --- | --- | --- | --- |
| Exposure |  |  |  |  |
| Body Mass Index | ieu-b-40 | UKB | 681,275 | European |
| Waist circumference | ebi-a-GCST90014020 | UKB | 407,661 | European |
| Waist-to-hip ratio | ebi-a-GCST90025996 | UKB | 458,349 | European |
| Visceral adipose tissue volume | ebi-a-GCST90016671 | UKB | 32,860 | European |
| Body fat percentage | ukb-a-264 | UKB | 331,117 | European |
| Outcome |  |  |  |  |
| Cognitive performance | ebi-a-GCST006572 | MRC-IEU | 257,841 | European |
| Dementia | finn-b-F5_DEMENTIA | FinnGen | / | European |
| Cognitive function | ieu-b-4837 | Within family GWAS consortium | 9,997 | European |
| Alzheimer's disease | ieu-a-297 | IGAP | 74,046 | European |

Abbreviations: GWAS, indicated genome-wide association studies; UKB, the UK Biobank; MRC-IEU, MRC Integrative Epidemiology Unit; GIANT, The Genetic Investigation of ANthropometric; IGAP , Traits International Genomics of Alzheimer’s Project.

Supplementary Table 6. Characteristics of studies included

| Author | Year | Design | Duration of follow-up | Country, Region | Sample size | Age/mean±  SD or range, years | OR/RR/HR | BMI categories, kg/m^2^ | Body fat percentage | Central obesity/ visceral fat | Disease type | Disease assessment | Adjusted covariates |
| --- | --- | --- | --- | --- | --- | --- | --- | --- | --- | --- | --- | --- | --- |
| F Nourhashémi ^[73]^ | 2003 | Cohort | 8 y | France, Europe | 3,557 | 77.6±6.4 | Underweight:1.185  Obesity:0.716  Overweight:0.709 | <21: Underweight;  >27: Obesity. | / | None | Dementia | DSM-IIIR | Sex, age, age-sex interaction, educational level, alcohol and tobacco consumption. |
| George Razay ^[75]^ | 2006 | Case-control | None | England and Australia, Mixed | 125 | 75.0±7.9 | Underweight:7.9  Obese:12.6  Overweight:1.8  WHR:2 | <20: Underweight; 20.0-24.9: Normal weight; 25-29.9: Overweight; ≥30: Obese. | / | WHR: >0.9 in men or >0.8 in women | AD | DSM-IV, NINCDS-ADRDA | Age, sex, location, systolic blood pressure, cholesterol, and glucose. |
| Kathleen M Hayden ^[55]^ | 2006 | Cohort | 5 y | US, North America | 3,264 | 74.0±6.4 | Obesity:1.93 | >30: Obesity. | / | None | Dementia, AD | DSM-III-R | Age, sex, APOE genotype, hypertension, high cholesterol, diabetes, stroke, CABG, and MI. |
| Majon Muller ^[92]^ | 2007 | Cohort | 4.4 y | US, North America | 2,476 | 76.8 | WC/AD:0.6  WC/Overall dementia:0.7 | None | / | WC: >0.94 in men or >0.80 in women | Dementia | DSM-IV, NINCDS-ADRDA | Age, sex, education, ethnic group, APOE allele, and smoking. |
| José A Luchsinger ^[67]^ | 2007 | Cohort | 5.1 y | US, North America | 893 | 77.0±5.7 | Overweight:0.6  Obesity:0.8  WC:1 | 26.3-29.6：Overweight:  >29.6：Obesity | / | The WC was used as continuous variables and categorized by quartiles. | Dementia | DSM-IV criteria; NINCDS-ADRDA; World Health Organization stroke criteria | Age, sex, years of education, ethnic group, apolipoprotein E-ε4 status. |
| Anna Rita Atti ^[42]^ | 2008 | Cohort | 9 y | Sweden, Europe | 1,255 | 80.8 | Overweight:& Obesity: 0.75  Underweight: 0.97 | <20: Underweight; >25: Overweight and Obesity. | / | None | Dementia | DSM-III-R | Sex, age, education, baseline Mini-Mental State Examination score, depressive symptoms, chronic disease up to baseline, impairment in activities of daily living. |
| Annette L. Fitzpatrick ^[54]^ | 2009 | Cohort | 10 y | US, North America | 2,798 | 74.4 | Underweight/AD: 1.42  Overweight/AD: 0.74  Obese/AD: 0.58 | <20: Underweight; 25-30: Overweight; >30: Obesity. | / | None | Dementia, AD | NINCDS-ADRDA | Age, race, sex, years of education, C-reactive protein level, interleukin 6 level, hypertension status, diabetes mellitus status, coronary heart disease, total cholesterol level, ankle-arm index, smoking status, kilocalories expended per week, apolipoprotein E ε4 allele. |
| Nancy A West ^[81]^ | 2009 | Cohort | 5.6 y | US, North America | 1,351 | 69.9 | Overweight: 0.46  obese: 0.24 | <25.0: Underweight; 25.0-29.9: Overweight; ≥30: Obese. | / | WC：43.3–88.9, 91.44–101.6, and 104.14–142.24 cm in women；40–88.9, 91.4–101.6, and 102–147.32 cm in men | Cognitive impairment | 3MSE and DelRec | Age, sex, education, waist circumference tertile, and height. |
| Christelle Raffaitin ^[93]^ | 2009 | Cohort | 4 y | France, Europe | 7,087 | 73.4±4.9 | WC/dementia: 0.83  WC/Alzheimer disease: 0.64 | None | / | WC: >102 cm in men or >88 cm in men | Dementia, AD | DSM-IV | Age, sex, educational level, and city center. |
| Yan Zhou ^[90]^ | 2010 | Cross-sectional | None | China, Asia | 638 | 93.36 | Normal weight: 1.107  Overweight: 1.145  Obesity: 1.15 | <18.5: Underweight; 18.5–23.0: Normal weight; 23.0–27.5: Overweight; ≥27.5: Obesity. | / | None | Dementia | MMSE | Age, gender, educational levels, the other components of metabolic syndrome and lifestyles. |
| Kenji Kamogawa ^[105]^ | 2010 | Cross-sectional | None | Japan, Asia | 517 | 72 | None | None | / | VFA; SFA | MCI | Consortium to Establish a Registry for Alzheimer’s Disease 10-word recall test | Age, leptin, adiponectin, either abdominal visceral fat area or subcutaneous fat area based on the significant association between abdominal fat area. |
| Yen-Ching Chen ^[46]^ | 2010 | Case-control | None | China, Asia | 554 | 77.6±8.2 | Underweight: 3.8  Overweight: 0.7 | <18.5: Low weight;  18.5-23.9: Normal weight; 24-26.9: Overweight; ≥27: Obesity. | / | None | AD | MMSE | Age, religion, diabetes mellitus, and marital status (men were additionally adjusted for smoking status). |
| Paola Forti ^[94]^ | 2010 | Cohort | 3.9 y | Italy, Europe | 749 | 73.3±6.1 | WC/Alzheimer disease: 0.63  WC/dementia: 0.61 | None | / | WC: >102 cm in men or >88 cm women | Dementia,  AD | DSM-IV | Age, sex, education, apolipoprotein E e4 carrier status, sedentary lifestyle, cardiovascular disease, history of stroke, hyperhomocysteinemia, inflammation status, and all of the other criteria. |
| Christiane Reitz ^[104]^ | 2010 | Cohort | 4 y | US, North America | 1,051 | 75.66±6.32 | WHR: 1.13 | None | / | None | AD | DSM-IV | Age, sex, education, and ethnicity. |
| Vincenzo Solfrizzi ^[95]^ | 2010 | Cohort | 3.5 y | Italy, Europe | 2,097 | 72.94±5.56 | WC/dementia: 0.58  WC/Alzheimer disease: 0.38  WC/other dementia: 0.89 | None | / | WC:>102 cm for men or >88 cm for women | Dementia, AD | DSM-III- R | Age, sex, education, Geriatric Depression Scale score, drink per day, smoking status in pack-years, fibrinogen, non-HDL cholesterol, ratio of apolipoprotein B to apolipoprotein A–I, coronary artery disease and stroke. |
| Brian D Power ^[41]^ | 2011 | Cohort | 7.1 y | Australia, Oceania | 12,047 | 72.1±4.4 | WC≥102cm/men: 0.88  WHR≥0.9/men: 0.82  Overweight/men: 0.82  Obese/men: 0.82 | 18.5-25: Normal weight; 25-30: Overweight; ≥30: Obese. | / | WC: ≥102cm for man; WHR: ≥0.9 | Dementia | ICD-9 and ICD-10 codes | Age, marital status, education, alcohol consumption, fat intake from milk, physical activity, and prevalent diabetes, dyslipidaemia, and coronary heart disease. |
| Dae Hyun Yoon ^[96]^ | 2012 | Cross-sectional | None | Korea, Asia | 250 | ≥60 | WC: 1.357 | None | / | WC: >90 cm for men or >80 cm for women;  VAT; SFA | MCI | MMSE-KC | Age, sex, education, hypertension, diabetes |
| Won-Sock Chang ^[44]^ | 2012 | Cross-sectional | None | Korea, Asia | 293 | 60-89 | Overweight: 2.2  Obesity: 2.41 | <18.5: Underweight; 18.5-23.0: Normal weight; 23.0-25.0: Overweight; ≥25.0: Obesity. | / | WC: ≥90 cm for men or ≥85 cm for women | Dementia | 7MSK | Age, sex, education, smoking, alcohol, exercise, hypertension, diabetes, and dyslipidemia. |
| Guo-Hong Cui ^[50]^ | 2013 | Cross-sectional | None | China, Asia | 2,809 | 70.6±6.6 | Underweight: 2.47  Overweight: 1.03  Obese: 0.99  WC: 1.42  WHR: 1.68 | <18.5: Underweight; 18.5-22.9: Normal; 23.0-24.9: Overweight; 25.0-29.9: Obese I; >30.0: Obese II. | / | WC: >90 cm for men or >80 cm for women; WHR: >0.90 for men or >0.85 for women | Cognitive impairment | MMSE | Age group, gender, education level, smoking, alcohol drinking, physical activity. |
| Renée F A G de Bruijn ^[51]^ | 2015 | Cohort | Original cohort: 8.3 y  Extended cohort: 8.9 y | Netherlands, Europe | RS-I: 7,003 RS-II: 2,953 | 69.4±9.1  65.0±8.3 | Underweight: 1.25  Overweight: 0.96  Obese: 0.84 | <18.5: Underweight; 25-30: Overweight; >30: Obesity. | / | None | Dementia | MMSE | Age, sex, hypertension, diabetes mellitus, total cholesterol/HDL cholesterol ratio, lipid-lowering medication, smoking, and educational. |
| Efstathios Papachristou ^[106]^ | 2015 | Cohort | 30 y | Britain, Europe | 1,570 | 71–92 | None | None | / | Visceral fat | Cognitive impairment | TYM | Sociodemographic characteristics, lifestyle factors and inflammatory or metabolic markers |
| Peggy J J Spauwen ^[107]^ | 2017 | Cohort | None | Iceland, Europe | 5,169 | 76 | None | None | / | VFA; SFA | MCI | DSM-IV | Educational level, smoking status, depressive symptoms, type 2 diabetes, hypertension, stroke, triglyceride/HDL ratio, high-sensitivity C-reactive protein and % change in weight from midlife |
| Ilaria Cova ^[49]^ | 2016 | Cohort | 2.4 y | Italy, Europe | 228 | 75 | Dementia: 2.49  AD: 2.24 | <20: underweight 25-29.9: Overweight ≥30: obese | / | None | Dementia, AD | CDR scale score | Age, sex, education, MMSE, MCI subtype, GDS, hypertension, diabetes, hypercholesterolemia, smoke, atrial fibrillation, cerebrovascular disease, cardiovascular disease, and ARWMC score |
| Johannes Baltasar Hessler ^[56]^ | 2016 | Cohort | 6.7 y | Germany, Europe | 3,547 | 67.3±7.6 | Overweight: 0.98  Obesity: 0.77 | <18.5：Underweight; 23-25: Overweight;  ＞25: Obesity; | / | None | Dementia | Health insurance claims data ICD-10 | Gender, age, marital status, health insurance, income, educational level, living place, physical activity, drinking, smoking status, comorbidity, baseline K-MMSE score, activities of daily livings, depression. |
| Jesper Skov Neergaard ^[72]^ | 2016 | Cohort | 15 y | Denmark, Europe | 5,512 | 70.6 | Underweight/ All-Cause Dementia: 0.88  Overweight/ All-Cause Dementia: 0.75  Obese/ All-Cause Dementia: 0.79  Underweight/ Alzheimer’s Disease: 0.92  Overweight/ All-Cause Dementia: 0.72  Obese/ All-Cause Dementia: 0.74 | <20: Underweight; 25-30: Overweight; >30: Obesity. | / | None | Dementia, AD | ICD-10 | Age, education level, smoking, alcohol consumption, physical activity, history of depression, history of cerebral embolism/hemorrhage, systolic blood pressure >160 mm Hg, fasting glucose levels, and total cholesterol levels >6.5 mmol/L, treatment for hypertension, diabetes, or hyperlipidemia, hyperglycemic or hyperlipidemic or hyperlipidemic. |
| Cecilia Albala ^[97]^ | 2016 | Cohort | 15 y | Chile, South America | 667 | 66±3.3 | WC:0.86 | None | / | WC:>88 cm in men or >102 cm in women | Dementia | MMSE | Sex, age, and education. |
| Sujin Kim ^[60]^ | 2016 | Cohort | 16 y | Korea, Asia | 5,125 | ≥ 65 | Underweight: 1.44  Overweight: 0.87  Obese: 0.73 | <18.5: Underweight; 23-24.9: Overweight; >25: Obesity. | / | None | Cognitive impairment | K-MMSE | Age, sex, and education. |
| Tze Pin Ng ^[98]^ | 2016 | Cohort | 3 y | Singapore, Asia | 1,519 | 64.9±6.8 | WC: 1.41 | None | / | WC: ≥90 cm for men or ≥80 cm for women | Cognitive impairment | MMSE | Sex, age, education, APOE-ε4 genotype, smoking, and physical, social, and productive activities score. |
| Tzeyu L Michaud ^[70]^ | 2017 | Cohort | 4.3y | US, North America | 8,691 | 75 | Underweight/ MCI: 1.12  Underweight/ AD: 2.12  Underweight/ Dementia: 1.31  Overweight/ MCI: 1.08  Overweight/ AD: 1.46  Overweight/ Dementia: 0.87 | <18.5: underweight 25–29.9: overweight ≥30: obesity | / | None | MCI, AD, Dementia | NACC | None |
| Feng Wang ^[80]^ | 2017 | Case-control | None | China, Asia | 960 | ≥60 | Underweight: 1.19  Overweight: 1.51  Obese: 1.52 | <18.5: Underweight; 18.5-22.9: Normal weight; 23.0-24.9: Overweight; ≥25: Obesity. | / | None | MCI | MMSE and MoCA | Gender, age, race, education level, medication history, apolopoprotein E4 carrier, onset of type 2 diabetes mellitus, hypertension, coronary heart disease and metabolism syndrome. |
| Jun Zhang ^[87]^ | 2017 | Cohort | 4.8 y | China, Asia | 16,791 | 60.1±7.4 | WC: 0.84  Underweight: 1.22  Overweight: 0.85  Obesity: 0.81 | <18.5: Underweight; 18.5-24.0: Normal;  24.0-28.0: Overweight; ≥28.0: Obesity. | / | WC: ≥90 cm for men and ≥80 cm for women | Cognitive impairment | MMSE | Study center, age, education, smoking, alcohol drinking, marital status, living conditions, physical activity, PHQ scores, systolic blood pressure at baseline, mean systolic blood pressure during the follow- up, estimated glomerular filtration rate, new stroke, diabetes mellitus at baseline, new diabetes mellitus during the follow-up, and treatment allocation. |
| Yanbo Li ^[63]^ | 2018 | Cross-sectional | None | China, Asia | 865 | 64.1±6.7 | Overweight: 0.770  Obesity: 0.637  WC: 0.722  WHR: 0.869 | ≤23.9: Non-obesity; 24.0–27.9: Overweight; ≥28.0: General Obesity. | / | WC: ≥90cm for men or ≥85 cm for women; WHR: ≥0.90 for men or ≥0.85 for women | Cognitive impairment | MMSE | Age, Sex, Educational level, Marital status, Smoking, Drinking, Lack of physical activity, Hypertension, CHD, Dyslipidemia, Triglycerides, HDL-C, LDL-C. |
| Ilse A C Arnoldussen ^[91]^ | 2018 | Cohort | 10 y | Sweden, Europe | 924 | 70 | Underweight: 2.94  Overweight: 0.79  Obese: 0.93  WHR: 0.8 | <20.0: underweight 25.00–29.99: overweight ≥30.0: obese | / | WHR: >0.90 in men, >0.85 in women | Dementia | DSM-III-R | Age, sex, SES, ApoE ε4 allele, and smoking |
| Soo Hyun Joo ^[59]^ | 2018 | Cohort | 7 y | Korea, Asia | 388 | 74.5±7.6 | Underweight: 2.38  Overweight: 0.78  Obese: 0.71 | <18.5: underweight 25–29.9: overweight ≥30: obesity | / | None | AD | DSM-IV criteria; NINCDS-ADRDA; World Health Organization stroke criteria | Age, sex, and education, CERAD-K total score, cognitive intervention, hypertension, diabetes mellitus, hyperlipidemia, heart disease, and cerebrovascular disease |
| Archana Singh-Manoux ^[77]^ | 2018 | Cohort | 30 y | London, Europe | 10,308 | 50.5±4.5 | Underweight: 1.21  Overweight: 0.97  Obese: 1.37  WHR: 0.932 | ≤18.5: Underweight; 18.5-24.9: Normal; 25.0-29.9: Overweight; ≥ 30: Obese. | / | WHR: ≥1.0 in men and 0.85 in women | Dementia | ICD-10 | Age, sex, education, diabetes, CVD and CVD medication. |
| Geum Joon Cho ^[47]^ | 2019 | Cohort | 6.46 y | Korea, Asia | 872,082 | 70.4±4.7 | WC: 1.199  Underweight: 1.24  Overweight: 0.89  Obese: 0.791 | <18.5: Underweight; 18.5-23: Normal weight; 23-25: Overweight; 25-27.5: Obesity-grade 1; 27.5-30: Obesity-grade 2; ≥30: Obesity-grade 3. | / | WC: < 65, 65 to < 70, 70 to < 75, 75 to < 80, 80 to < 85 (reference for women), 85 to < 90 (reference for men), 90 to < 95, 95 to < 100, 100 to < 105, 105 to < 110, and ≥ 110 cm | Dementia | ICD-10 | Age; BMI; alcohol consumption; smoking and exercise status; systolic blood pressure; fasting blood glucose; high-density lipoprotein cholesterol; low-density lipoprotein cholesterol; aspartate aminotransferase; alanine aminotransferase; economic status; history of diabetes, hypertension, and cardiovascular disease; and Charlson Comorbidity Index. |
| Yumika Kotaki ^[61]^ | 2019 | Cohort | 5.7 y | Japan, Asia | 8,563 | 73.1 | Obesity: 1.41 | >30: Obesity | / | None | Dementia | LTCI system | Age, sex, diabetes mellitus, hypertension, body mass index, time spent walking per day, Kessler 6-Item Psychological Distress Scale score, educational level. |
| Ji-Yeon Lee ^[62]^ | 2019 | Cohort | 65 months | Korea, Asia | 363,932 | ≥60 | Obesity/ Overall Dementia: 0.9  Obesity/ AD: 0.88 | ≥25: Obesity | / | None | Dementia,  AD | ICD-10 | Age, sex, tobacco and alcohol use, exercise, and socioeconomic status. |
| Zhizhen Liu ^[66]^ | 2019 | Cross-sectional | None | China, Asia | 1,037 | ≥70 | WC: 1.858  WHR: 1.98  Overweight: 0.860  Obese: 2.77 | <24: Normal; 24.0–27.9: Overweight; ≥28.0: Obese. | / | WC: ≥0.85 m for male and >0.80 m for female; WHR: ≥0.9 for male and ≥0.85 for female | Cognitive impairment | MMSE | Gender, age, level of education, lifestyle such as smoking,  alcohol consumption and exercise and blood biomarkers. |
| Supa Pengpid ^[74]^ | 2019 | Cross-sectional | None | Indonesia, Asia | 1,228 | 65 | Underweight: 1.18 | <18.5: Underweight. | / | None | Dementia | TICS | None. |
| Qingtao Hou ^[57]^ | 2019 | Cross-sectional | None | China, Asia | 1,100 | 60–98 | Underweight: 0.876  Overweight: 0.458  Obesity: 0.758 | <18.5: Underweight; 18.5–23.9: Normal weight; 24.0–27.9: Overweight; ≥28.0: Obesity. | / | WHR: ≥0.9 for males and ≥0.85 for females | Cognitive impairment | MMSE | Age, gender, smoking, drinking, education level, hypercholesterolemia, hypertension, and diabetes. |
| Zhi Cao ^[43]^ | 2020 | Cohort | 8.1 y | China, Asia | 466,980 | 71 | Overweight: 0.81  Underweight: 1.91  Obese: 0.78 | <18.5: Underweight; BMI 25-30: Overweight;  ≥30: Obesity. | 0.49(  0.41,  0.58 ) | None | Dementia | ICD-10 | Age, sex, Townsend deprivation index, income, educational attainment, employment, ethnicity, physical activity, alcohol intake, smoking status, C-reactive protein, total cholesterol, hypertension, diabetes, cardiovascular disease, cancer, long-illness, serum urate. |
| Hiroshi Yokomichi ^[83]^ | 2020 | Cohort | 5.8 y | Japan, Asia | 3,696 | 73.4 | Obese: 0.775  Underweight: 1.43 | <18.5: Underweight; 25–29.9: Obesity grade 1; ≥30: Obesity grades 2–4. | / | None | Dementia | LTCI | Age, history of stroke, educational background, income, number of family members, marital status, and frequency of meeting friends. |
| Chendi Cui ^[14]^ | 2020 | Cohort | 6 y | US, North America | 344 | 77.9 | Overweight: 0.61  Underweight: 0.77  Obese: 0.71 | <20: Underweight,  20–25: Normal weight; >25–30: Overweight; >30: Obese | / | None | Dementia | 3MSE | Age, race, education, APOEε4, diabetes, hypertension, physical activity, triglycerides, current smoking status, alcohol consumption, depression scale, TIA/stroke history, cancer history, diagnosis of COPD. |
| Yixuan Ma ^[68]^ | 2020 | Cohort | 15 y | England, Europe | 5,538 | 63.4 | WC: 1.39  Overweight: 1.27  Obese: 1.31 | 18.5–24.9: Normal weight;  25–29.9: Overweight; ≥30: Obese. | / | WC: >102cm for men and >88cm for women | Dementia | IQCODE and HES data | Age, APOE E4, education, marital status, smoking status, physical activity, hypertension. |
| Amelia Nur Vidyanti  ^[79]^ | 2020 | Cross-sectional | None | Indonesia, Asia | 143 | >60 | Overweight: 1.5  Underweight: 5.312  Obese: 0.424 | <18.5: Underweight; 18.5–22.9: Normal; 23–27.4: Overweight; ≥27.5: Obese. | / | None | Cognitive impairment | MoCA-INA | Sociodemographic and comorbidities. |
| Ji Eun Lee ^[99]^ | 2020 | Cohort | 4.9 y | Korea, Asia | 4,106 | 55.8±10.1 | WC/ dementia: 0.97  WC/ Alzheimer disease: 0.99 | None | / | WC: ≥90 cm for men and ≥85 cm for women | Dementia, AD | MMSE | Age, sex, smoking, alcohol, regular exercise, stroke, depression, and chronic kidney disease. |
| I Chiba ^[108]^ | 2020 | Cross-sectional | None | Japan,  Asia | 6,109 | 74.3±5.12 | None | None | / | VFA | MCI | NCGG-FAT | Age, educational level, comorbidities, GDS scores, smoking and drinking condition, frequency of sports or exercise |
| John A Batsis ^[100]^ | 2021 | Cohort | 8 y | US, North America | 5,822 | ≥65 | WC: 0.99  Obesity: 0.98 | ≥30: Obesity | / | WC: ≥88 cm for women or ≥102 cm for men | Cognitive impairment | AD-8 score and 3 domains: memory, orientation, and executive function | Age category, sex, smoking, status, education, and comorbidities. |
| X Liu ^[109]^ | 2021 | Cross-sectional | None | China, Asia | 3,914 | ≥60 | None | None | / | VFA | MCI | SPMSQ | Ethnic groups, marriage status, education level, occupation and life-style factors (smoking, drinking alcohol and tea), sleeping quality, fertility status, chronic disease comorbidities, depression status |
| Yeo Jin Kim ^[101]^ | 2021 | Cohort | 8 y | Korea, Asia | 84,144 | ≥60 | WC: 1.88 | None | / | WC: >90 cm in males and >80 cm in the female | Dementia | ICD-10-CM | Age, sex, smoking, alcohol, physical inactivity, previous stroke, previous cardiac disease, and metabolic syndrome or 5 metabolic syndrome components. |
| Shanshan Wu ^[82]^ | 2021 | Cohort | 5.9 y | China, Asia | 12,027 | 81.23±10.72 | Overweight: 0.86  Underweight: 1.02  Obese: 1.01 | <18.5: Underweight; 18.5-23.9: Normal weight;  24-27.9: Overweight;  ≥28: Obese. | / | None | Cognitive impairment | MMSE | Type of residence, marital status, education, living arrangement, smoking status, drinking status, regular exercise, vegetables, and fruit intake. |
| Adina Zeki Al Hazzouri ^[86]^ | 2021 | Cohort | 7.9 y | US, North America | 5,104 | 70-89 | Overweight: 0.64  Obese: 0.449 | <25: Normal; 25-30: Overweight;  >30: Obese | / | None | Dementia | 3MS | Age, sex and race/ethnicity, years of education, cohort, and BMI. |
| Zheng Ren ^[76]^ | 2021 | Cohort | 4 y | China, Asia | 5,156 | ≥65 | Underweight: 1.258 | <18.5: Underweight; 18.5-24.0: Normal weight; ≥24.0: Overweight/Obesity | / | WC: ≥85cm for males and ≥80cm for females | Cognitive  impairment | MMSE | BMI, WC, and WHtR. |
| Yemin Yuan ^[85]^ | 2021 | Cross-sectional | None | China, Asia | 3,242 | 70.14±6.17 | Overweight: 0.86  Underweight: 2.08  Obese: 0.55 | <18.5: Underweight; 18.5-24.0: Normal;  24.0-28.0: Overweight; ≥28.0: Obesity. | / | None | MCI | MMSE | Age, gender, education, marital status, household income, cigarette, alcohol consumption, physical activity, activities of daily livings, mental health. |
| Jing-Jing Zhang ^[88]^ | 2021 | Cross-sectional | None | China, Asia | 8,221 | 71.96±5.895 | Overweight: 0.89  Underweight: 1.25  Obese: 1.11 | <18.5: Underweight; 18.5–23.9: Normal weight; 24.0–27.9: Overweight; ≥28: Obesity. | / | None | Cognitive impairment | DSM–IV | Age, sex, education level, physical exercise, smoking, drinking, hypertension, diabetes, and cerebrovascular disease. |
| Michael P Bancks ^[102]^ | 2021 | Cohort | 10 y | US, North America | 977 | 63.5±9.6 | WC: 0.81 | None | / | WC: ≥105 cm for women and ≥110 cm for men | Dementia | ICD-9 and ICD-10 | Age at exam, sex, race/ethnicity, diabetes medication use, field center, educational attainment, family income, smoking status, alcohol use, physical activity, systolic blood pressure, blood pressure medication use, low- density lipoprotein cholesterol, and cholesterol medication use. |
| Shou-En Wu ^[103]^ | 2021 | Cohort | 10 y | China, Asia | 5,693 | 63.6±2.7 | WC/ cognitive impairment: 1.065  WC/ dementia: 1.147 | None | / | WC: >90 for men and >80 cm for women | Dementia,  Cognitive impairment | MMSE | Age, gender, education level, smoking, coronary artery disease, hypertension, and type 2 diabetes. |
| Yue-Ting Deng ^[52]^ | 2022 | Cohort | 8.74 y | England, Europe | 322,336 | 62.24±5.41 | Underweight: 1.35  Overweight: 0.8  Obese: 0.872 | <18.5: Underweight; 18.5–24.9: Normal weight; 25.0–29.9: Overweight; 30.0–34.9: Class I Obesity; 35.0–39.9: Class II Obesity; >40.0: Class III Obesity | 0.37( 0.27, 0.51) | None | Dementia | ICD-9 and ICD-10 | Townsend index, depression status, smoking status, alcohol consumption status. |
| Na Chen ^[45]^ | 2022 | Cross-sectional | None | China, Asia | 9,218 | ≥80 | Underweight: 1.296  Overweight: 1.045  Obese: 1.057 | <18.5: Underweight; 18.5-25: Normal;  25-30: Overweight;  >30: Obese | / | None | Cognitive impairment | MMSE | Age, years of schooling, marital status, economic status, physical activity, smoking, drinking, ADL, hypertension, dyslipidaemia, diabetes, heart disease, stroke, cancer. |
| Su Hwan Cho ^[48]^ | 2022 | Cohort | 10 y | Korea, Asia | 148,534 | ≥65 | Underweight: 1.17  Overweight: 0.9  Obese: 0.83 | <18.5: Underweight;  18.5-22.9: Normal weight; 23.0-24.9: Overweight;  ≥25.0: Obese | / | None | AD | ICD-10 | Smoking, alcohol consumption, regular exercise, low-income status, hypertension, diabetes mellitus, dyslipidemia, and cardiovascular disease. |
| Louis Jacob ^[58]^ | 2022 | Cohort | 14 y | Germany, Europe | 296,767 | 70.2±5.9 | Underweight/ Dementia: 1.21  Overweight /Dementia: 0.94  Obese/ Dementia: 0.98  Underweight/ AD: 1.09  Overweight/ AD: 0.91  Obese/ AD: 0.82 | <18.5: Underweight;  18.5-25: Normal;  25-30: Overweight;  >30: Obese | / | None | Dementia | ICD-10 | None |
| Fang Liang ^[64]^ | 2022 | Cohort | 3-4 y | China, Asia | 4,792 | 80.70±9.58 | Underweight: 1.42  Overweight: 0.79  Obese: 0.72 | <18.5: Underweight;  18.5-24: Normal; 24-28: Overweight;  ≥28: Obese | / | None | Cognitive impairment | MMSE | Sex, age, residence, education, occupation, smoking status, alcohol consumption, regular exercise, financial independence, and health conditions. |
| Wan-Yu Lin ^[65]^ | 2022 | Cross-sectional | None | China, Asia | 30,697 | 60-70 | Underweight: 0.816  Overweight: 1.063  Obese: 1.119  WC: 1.196  WHR: 1.225 | ≥27: Obese | 1.06( 0.97, 1.17) | WC: ≥90 cm for males or ≥80 cm for females; WHR: ≥0.90 for males or ≥0.85 for females | Cognitive impairment | MMSE | Age, smoking status, drinking status, regular exercise, chronic disease status, depression status, blood pressure level, total cholesterol, fasting glucose, and educational attainment. |
| Yuki Someya ^[78]^ | 2022 | Cross-sectional | None | Japan, Asia | 1,615 | 73.1 | Obesity: 1.67 | ≥25: Obesity | / | None | Dementia | MMSE | Age, sex, year of education, physical activity, hypertension, diabetes, dyslipidemia, and depression status. Bold values indicate statistical significance. |
| Wenshuo Dong ^[53]^ | 2023 | Cohort | 7 y | Korea, Asia | 6,311 | 60-93 | Underweight: 1.473  Overweight: 0.874  Obesity: 0.874 | <18.5: underweight;  18.5–23.9: normal weight  ≥24.0: over weight or obese | / | None | Cognitive impairment | Mini-Mental State Examination | Age, gender, education, residence area, marital status, average household income, occupation, smoking status, alcohol consumption, hearing status, participation in social activities, nighttime sleep duration, ADL limitations, and depression. |
| Apisit Manacharoen ^[69]^ | 2023 | Cohort | 5 y | Thailand, Asia | 112 | 60.0 ± 4.3 | Underweight/ Dementia: 2.72 Overweight/ Dementia: 0.9 Obesity/ Dementia: 1.06  Underweight/ MCI: 1.63  Overweight/MCI: 1.49  Obesity/MCI: 2.04 | <18.5:underweight;  18.5–22.9: normal weight;  23.0–24.9: over weight;  ≥25.0: obese | / | None | Dementia, MCI | The Thai version of the MoCA, face-  to-face clinical diagnosis following the Diagnostic and  Statistical Manual of Mental Disorders (DSM-5) criteria | Age, education, type 2 diabetes, Sex, Body mass index, Hypertension, Ischemic heart disease, Thyroid disorder, Stroke, Smoking, Alcohol drinking. |
| Xiaoguang Zhao ^[89]^ | 2023 | Cross-sectional | None | China, Asia | 4,839 | ≥60 | Overweight: 0.82  Obesity: 0.85  Normal weight: 18.5-24.0  Over weight: 24.0-28.0: | ≥28: Obesity | / | None | Cognitive impairment | TICS | Age, education level, marital status, residence, drinking frequency, smoking status, sleep duration, and number of chronic diseases. |
| Shiqi Yuan ^[84]^ | 2022 | Cohort | 10 y | America | 475,813 | 62.0±12.8 | Underweight: 1.585  Overweight: 0.741 | <23: underweight;  23-30: normal weight;  >30: over weight | / | None | AD | tests from the Wechsler Adult Intelligence  Scale, the Halstead-Reitan Neuropsychological Battery, the  Wechsler Memory Scale Revised, and the Mini-Mental State  Examination. | age,education,gender,number of prior examinations, smoking, the ratio of triglycerides (mmol/l) to high density  lipoprotein cholesterol (mmol/l; TG/HDL-C), prevalent  CVD, high sensitivity C-reactive protein (CRP; mg/l),  systolic blood pressure (SBP; mm Hg), depressed mood,  glucose (mg/dl), and physical activity (PA; metabolic  equivalent task (MET)-hours per week). |
| Ginny Natale ^[71]^ | 2023 | cohort | 5 y | America | 6078 | 77.49±7.79 | Underweight/ Dementia: 2.28 Overweight/ Dementia: 0.7 Obesity/ Dementia: 0.44  Underweight/ MCI: 1.62  Overweight/MCI: 1.01  Obesity/MCI: 0.83 | <18.5: underweight;  18.6-24.9:normal weight; 25-29.9: over weight; >30: obese | / | None | Dementia, MCI | The AD8 Dementia Screening Interview | Diabetes, Vigorous Activities, Cardiovascular disease, age, Sex, Race/ethnicity and Education. |

*Note*: BMI, body mass index; AD, Alzheimer’s disease; ICD-9: international classification of diseases, ninth revision; WHR, waist-to-hip ratio; WHtR: waist-to-height ratio; DSM-III-R: diagnostic and statistical manual of mental disorders, 3rd edition, revised; APOE: apolipoprotein E; MI, myocardial infarction; CABG: coronary artery bypass graft surgery; WC: waist circumference; NINCDS-ADRDA: neurological and communicative disorders (NINCDS) and the stroke-Alzheimer's Disease and related disorders association (ADRDA); 3MSE: the modified mini-mental state examination; MCI: mild cognitive impairment, MoCA: Montreal cognitive assessment; DelRec: a verbal delayed word list recall test; RS-I, Rotterdam Study I, original cohort; RS-II, Rotterdam Study II, extended cohort; MMSE: mini-mental state examination; HDL: high-density lipoprotein; CHD: coronary heart disease; LDL-C: low-density lipoprotein cholesterol; PHQ: patient health questionnaire-9; CVD : cardiovascular disease; TIA: transient ischemic attack; COPD: chronic obstructive pulmonary disease; ICD-10: international classification of diseases tenth revision; 7MSK: Korean version of the seven minute screen test; ADL: activity of daily living; TICS: telephone interview for cognitive status; IQCODE: informant questionnaire on cognitive decline in the elderly; HES: Hospital Episodes Statistics data; MoCA-INA: Montreal cognitive assessment-Indonesia version; AD-8: Alzheimer’s Disease 8 score; ICD-10-CM: international classification of disease, tenth revision, clinical modification; K-MMSE: Korean mini-mental state examination; LTCI: long-term care insurance; NCGG-FAT: National Center for Geriatrics and Gerontology-Functional; MMSE-KC: Korean Version of the Mini-Mental State Examination for the Consortium to Establish a Registry for Alzheimer’s Disease Assessment Tool; SPMSQ: Short Portable Mental Status Questionnaire; TYM: Self-administered cognitive screening test; VFA: visceral fat areas: SFA: subcutaneous fat area; CDR: Clinical Dementia Rating; SES: socio-economic status; NACC: National Alzheimer’s Coordinating Center; ARWMC: Age-Related White Matter Changes scale.

Supplementary Table 7. Risk of bias assessment (Newcastle-Ottawa Quality Assessment Scale criteria)

| Study | Selection | | | | | | Comparability | | | | Outcome | | | | | | | Quality  score |
| --- | --- | --- | --- | --- | --- | --- | --- | --- | --- | --- | --- | --- | --- | --- | --- | --- | --- | --- |
|  | Representativeness of the exposed cohort | Selection of the non-exposed cohort | | Ascertainment of exposure | Demonstration that the outcome of interest was not present at start of the study | | Comparability of cohorts on the basis of the design or the analysis | | | | Ascertainment of outcome | | | Was follow-up long enough for outcomes to occur? | | | Adequacy of follow-up of cohorts |  |
| Cohort study | | | | | | | | | | | | | | | | | | |
| F Nourhashémi (2003) ^[73]^ | * | * | | * | * | | * | | * | | | | * | | | * | | 8 |
| Kathleen M Hayden (2006) ^[55]^ | - | * | | * | * | | * | | * | | | | - | | | * | | 6 |
| Majon Muller (2007) ^[92]^ | - | * | | * | * | | * | | * | | | | - | | | * | | 6 |
| José A Luchsinger (2007) ^[67]^ | * | * | | * | * | | * | | * | | | | * | | | * | | 8 |
| Anna Rita Atti (2008) ^[42]^ | * | * | | * | * | | ** | | * | | | | * | | | * | | 9 |
| Annette L. Fitzpatrick (2009) ^[54]^ | - | * | | * | * | | * | | * | | | | * | | | * | | 7 |
| Nancy A West (2009) ^[81]^ | * | * | | * | * | | * | | * | | | | * | | | * | | 8 |
| Christelle Raffaitin (2009) ^[93]^ | * | * | | * | * | | ** | | * | | | | * | | | * | | 9 |
| Paola Forti (2010) [^94]^ | * | * | | * | * | | * | | * | | | | * | | | * | | 8 |
| Christiane Reitz (2010) ^[104]^ | - | * | | * | * | | * | | * | | | | - | | | * | | 6 |
| Vincenzo Solfrizzi (2010) [^95]^ | * | * | | * | * | | ** | | * | | | | - | | | * | | 8 |
| Brian D Power (2011) ^[41]^ | - | * | | * | * | | * | | * | | | | * | | | - | | 6 |
| Renée F A G de Bruijn (2015) [^51]^ | * | * | | * | * | | * | | * | | | | * | | | * | | 8 |
| Efstathios Papachristou (2015) ^[106]^ | - | * | | * | * | | * | | * | | | | * | | | - | | 6 |
| Ilaria Cova (2016) ^[49]^ | * | * | | * | * | | * | | * | | | | * | | | - | | 7 |
| Johannes Baltasar Hessler (2016) ^[56]^ | * | * | | * | * | | * | | * | | | | * | | | - | | 7 |
| Peggy J J Spauwen (2016) ^[107]^ | * | * | | * | * | | ** | | * | | | | - | | | * | | 8 |
| Sujin Kim (2016) ^[60]^ | - | * | | * | * | | * | | * | | | | * | | | - | | 6 |
| Jesper Skov Neergaard (2016) ^[72]^ | - | * | | * | * | | * | | * | | | | * | | | - | | 6 |
| Cecilia Albala (2016) ^[97]^ | - | * | | * | * | | * | | * | | | | * | | | - | | 6 |
| Tze Pin Ng (2016) ^[98]^ | * | * | | * | * | | ** | | * | | | | * | | | * | | 9 |
| Tzeyu L Michaud (2017) ^[70]^ | - | * | | * | * | | * | | * | | | | * | | | - | | 6 |
| Jun Zhang (2017) ^[87]^ | * | * | | * | * | | * | | * | | | | - | | | - | | 7 |
| Ilse A C Arnoldussen (2018) ^[91]^ | * | * | | * | * | | * | | * | | | | * | | | * | | 8 |
| Soo Hyun Joo (2018) ^[59]^ | * | * | | * | * | | ** | | * | | | | * | | | * | | 9 |
| Archana Singh-Manoux (2018) ^[77]^ | * | * | | * | * | | * | | * | | | | * | | | * | | 8 |
| Geum Joon Cho (2019) ^[47]^ | * | * | | * | * | | * | | * | | | | * | | | * | | 8 |
| Yumika Kotaki (2019) ^[61]^ | * | * | | * | * | | * | | * | | | | * | | | - | | 7 |
| Ji-Yeon Lee (2019) ^[62]^ | * | * | | * | * | | * | | * | | | | - | | | * | | 7 |
| Zhi Cao (2020) ^[43]^ | * | * | | * | * | | * | | * | | | | * | | | - | | 7 |
| Hiroshi Yokomichi (2020) ^[83]^ | - | * | | * | * | | * | | * | | | | * | | | - | | 6 |
| Chendi Cui (2020) ^[14]^ | * | * | | * | * | | * | | * | | | | * | | | * | | 8 |
| Yixuan Ma (2020) ^[68]^ | * | * | | * | * | | * | | * | | | | * | | | * | | 8 |
| Ji Eun Lee (2020) ^[99]^ | - | * | | * | * | | * | | * | | | | * | | | - | | 6 |
| John A Batsis (2021) ^[100]^ | * | * | | * | * | | * | | * | | | | * | | | - | | 7 |
| Yeo Jin Kim (2021) ^[101]^ | * | * | | * | * | | * | | * | | | | * | | | - | | 7 |
| Shanshan Wu (2021) ^[82]^ | * | * | | * | * | | ** | | * | | | | * | | | * | | 9 |
| Adina Zeki Al Hazzouri (2021) ^[86]^ | * | * | | * | * | | ** | | * | | | | * | | | * | | 9 |
| Zheng Ren (2021) ^[76]^ | * | * | | * | * | | * | | * | | | | - | | | * | | 6 |
| Michael P Bancks (2021) ^[102]^ | - | * | | * | * | | * | | * | | | | * | | | - | | 6 |
| Shou-En Wu (2021) ^[103]^ | * | * | | * | * | | * | | * | | | | * | | | - | | 7 |
| Yue-Ting Deng (2022) ^[52]^ | * | * | | * | * | | * | | * | | | | * | | | * | | 8 |
| Su Hwan Cho (2022) ^[48]^ | - | * | | * | * | | * | | * | | | | * | | | - | | 6 |
| Louis Jacob (2022) ^[58]^ | - | * | | * | * | | * | | * | | | | * | | | - | | 6 |
| Fang Liang (2022) ^[64]^ | * | * | | * | * | | * | | * | | | | - | | | * | | 7 |
| Xiaoguang Zhao (2023) ^[89]^ | * | * | | * | * | | * | | * | | | | - | | | * | | 8 |
| Apisit Manacharoen (2023) ^[69]^ | * | * | | * | * | | * | | * | | | | - | | | * | | 8 |
| Wenshuo Dong (2023) ^[53]^ | * | * | | * | * | | * | | * | | | | * | | | - | | 7 |
| Shiqi Yuan (2022) ^[84]^ | * | * | | * | * | | * | | * | | | | - | | | * | | 8 |
| Ginny Natale (2023) ^[71]^ | * | * | | * | * | | * | | * | | | | - | | | * | | 8 |
| case-control | | | | | | | | | | | | | | | | | | |
| George Razay (2006) ^[75]^ | * | * | | - | * | | * | | * | | | | * | | | - | | 6 |
| Yen-Ching Chen (2010) ^[46]^ | * | * | | - | * | | ** | | * | | | | * | | | - | | 7 |
| Feng Wang (2017) ^[80]^ | * | * | | - | * | | ** | | * | | | | * | | | - | | 7 |
| Quality assurance tool criteria* | | | | | | | | | | | | | | | | | | |
| Reference | 1 | 2 | 3 | 4 | 5 | 6 | | 7 | | 8 | | 9 | | | 10 | | 11 | Quality score |
| Yan Zhou (2010) ^[90]^ | Yes | Yes | Yes | Yes | Yes | No | | Unclear | | Yes | | Yes | | | Yes | | Yes | 9 |
| Kenji Kamogawa (2010) ^[105]^ | Yes | Yes | Yes | Yes | Yes | Unclear | | Yes | | Yes | | Unclear | | | Yes | | Unclear | 8 |
| Won-Sock Chang (2012) ^[44]^ | Yes | Yes | Yes | Yes | Yes | Unclear | | Yes | | Yes | | Unclear | | | Yes | | Yes | 9 |
| Dae Hyun Yoon (2012) ^[96]^ | Yes | Yes | Yes | Yes | Yes | Unclear | | Yes | | Yes | | Unclear | | | Yes | | Yes | 9 |
| Guo-Hong Cui (2013) ^[50]^ | Yes | Yes | Yes | Yes | Yes | Unclear | | Yes | | Yes | | Unclear | | | Yes | | Unclear | 8 |
| Yanbo Li (2017) ^[63]^ | Yes | Yes | Yes | Yes | Yes | Unclear | | Yes | | Yes | | Yes | | | Yes | | Yes | 10 |
| Zhizhen Liu (2019) ^[66]^ | Yes | Yes | Yes | Yes | Yes | Unclear | | Unclear | | Yes | | Unclear | | | Yes | | Yes | 8 |
| Supa Pengpid (2019) ^[74]^ | Yes | Yes | Yes | Yes | Yes | Unclear | | Unclear | | Yes | | Yes | | | Yes | | No | 8 |
| Qingtao Hou (2019) ^[57]^ | Yes | Yes | Yes | Yes | Yes | Yes | | Unclear | | Yes | | Unclear | | | Yes | | Yes | 8 |
| Amelia Nur Vidyanti (2020) ^[79]^ | Yes | Yes | Yes | Yes | Yes | Unclear | | Yes | | Yes | | Unclear | | | Yes | | Unclear | 8 |
| I Chiba (2020) ^[108]^ | Yes | Yes | Yes | Yes | Yes | Unclear | | Yes | | Yes | | Unclear | | | Yes | | Unclear | 8 |
| X Liu (2021) ^[109]^ | Yes | Yes | Yes | Yes | Yes | Unclear | | Yes | | Yes | | Unclear | | | Yes | | Unclear | 8 |
| Yemin Yuan (2021) ^[85]^ | Yes | Yes | Yes | Yes | Yes | Unclear | | Yes | | Yes | | Yes | | | Yes | | Yes | 10 |
| Jing-Jing Zhang (2021) ^[88]^ | Yes | Yes | Yes | Yes | Yes | Unclear | | Yes | | Yes | | Unclear | | | Yes | | Yes | 9 |
| Na Chen (2022) ^[45]^ | Yes | Yes | Yes | Yes | Yes | Unclear | | Unclear | | Yes | | Unclear | | | Yes | | Yes | 8 |
| Wan-Yu Lin (2022) ^[65]^ | Yes | Yes | Yes | Yes | Yes | Unclear | | Unclear | | Yes | | Unclear | | | Yes | | Yes | 8 |
| Yuki Someya (2022) ^[78]^ | Yes | Yes | Yes | Yes | Yes | Unclear | | Yes | | Yes | | Unclear | | | Yes | | Yes | 9 |

*Note:* *1. Was the research question or objective in this paper clearly stated?

2. Was the study population clearly specified and defined?

3. Was the participation rate of eligible persons at least 50%?

4. Were all the subjects selected or recruited from the same or similar populations (including the same time period)?

5. Were inclusion and exclusion criteria for being in the study prespecified and applied uniformly to all participants?

6. Was a sample size justification, power description, or variance and effect estimates provided?

7. For exposures that can vary in amount or level, did the study examine different levels of the exposure as related to the outcome (e.g., categories of exposure, or exposure measured as continuous variable)?

8. Were the exposure measures (independent variables) clearly defined, valid, reliable, and implemented consistently across all study participants?

9. Was the exposure(s) assessed more than once over time?

10. Were the outcome measures (dependent variables) clearly defined, valid, reliable, and implemented consistently across all study participants?

11. Were key potential confounding variables measured and adjusted statistically for their impact on the relationship between exposure(s) and outcome(s)?

Supplementary Figure 1. Sensitivity analysis of association between General, Central, Visceral Obesity and Body Fat Percentage with Cognitive disorders

（A）Sensitivity test for the impact of underweight (measured by BMI) on cognitive impairment.

（B）Sensitivity test for the impact of overweight (measured by BMI) on cognitive impairment.

（C）Sensitivity test for the impact of obesity (measured by BMI) on cognitive impairment.

（D）Sensitivity test for the impact of WC on cognitive impairment.

（E）Sensitivity test for the impact of WHR on cognitive impairment.

（F）Sensitivity test for the impact of visceral obesity on cognitive impairment.

（G）Sensitivity test for the impact of body fat percentage on cognitive impairment.

*Note*: BMI, body mass index; WC, waist circumference; WHR, waist-to-hip ratio.

Supplementary Figure 2. Funnel plot for assessment of publication bias


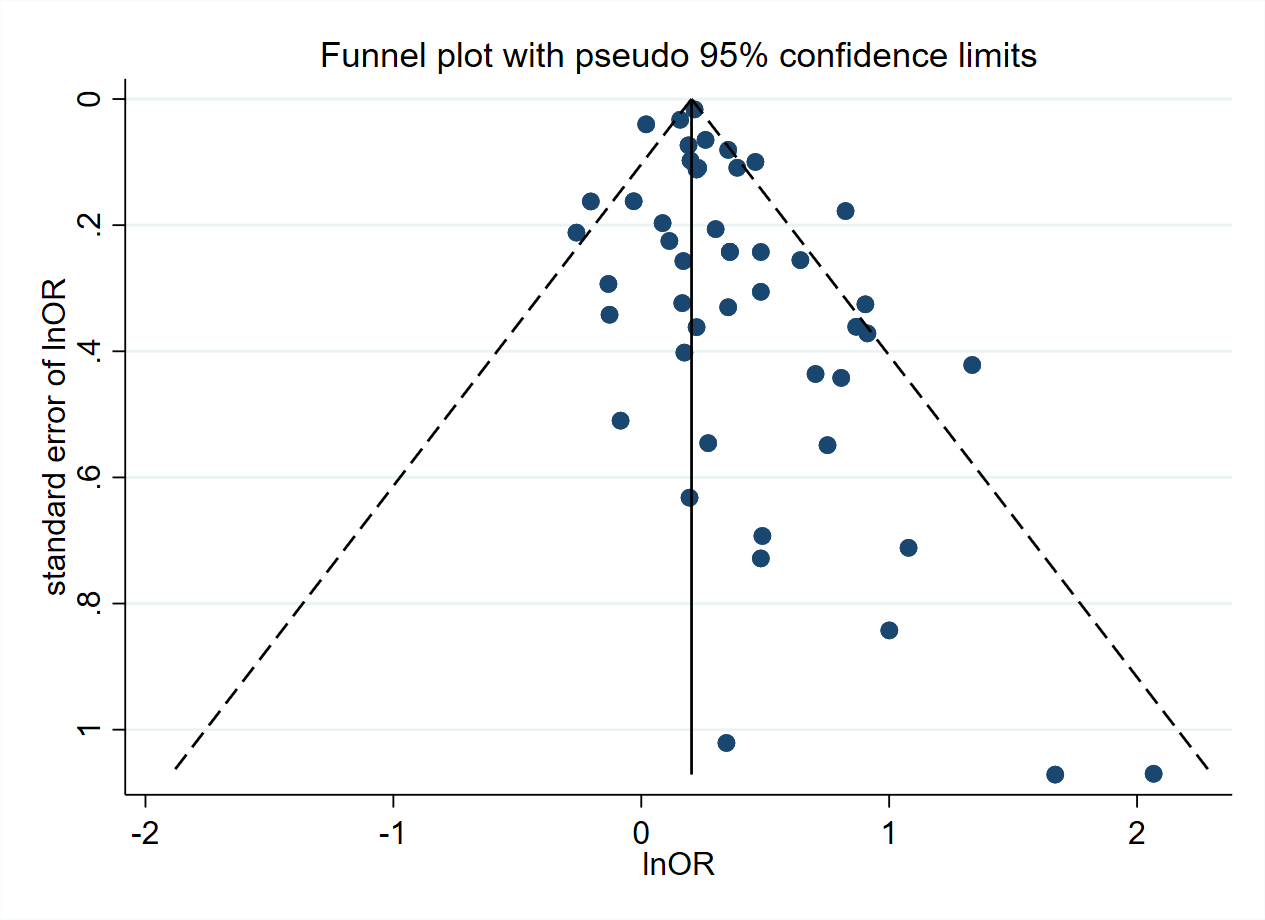


（A）Funnel plot of the impact of underweight (measured by BMI) and cognitive disorders.


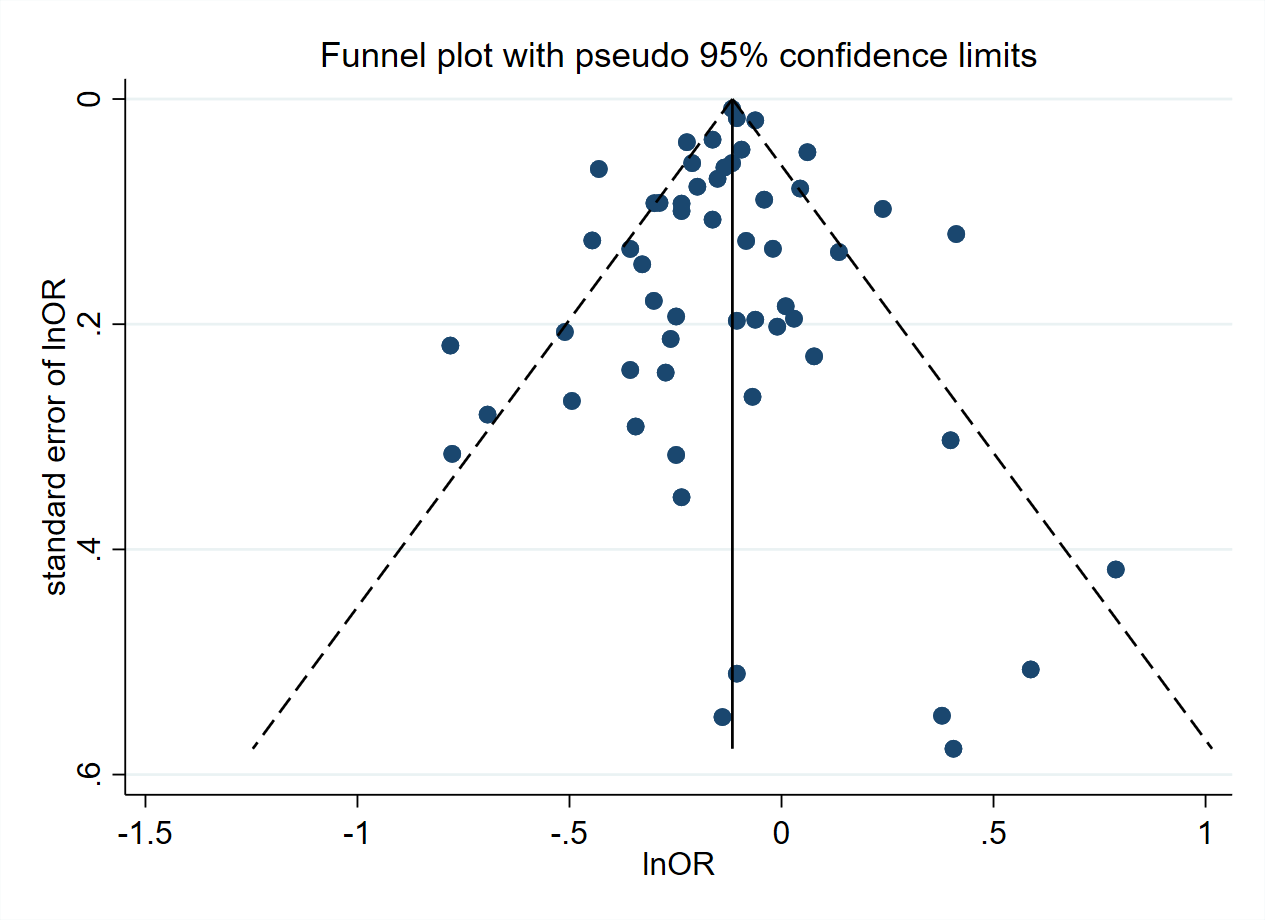


（B）Funnel plot of the impact of overweight (measured by BMI) and cognitive disorders.


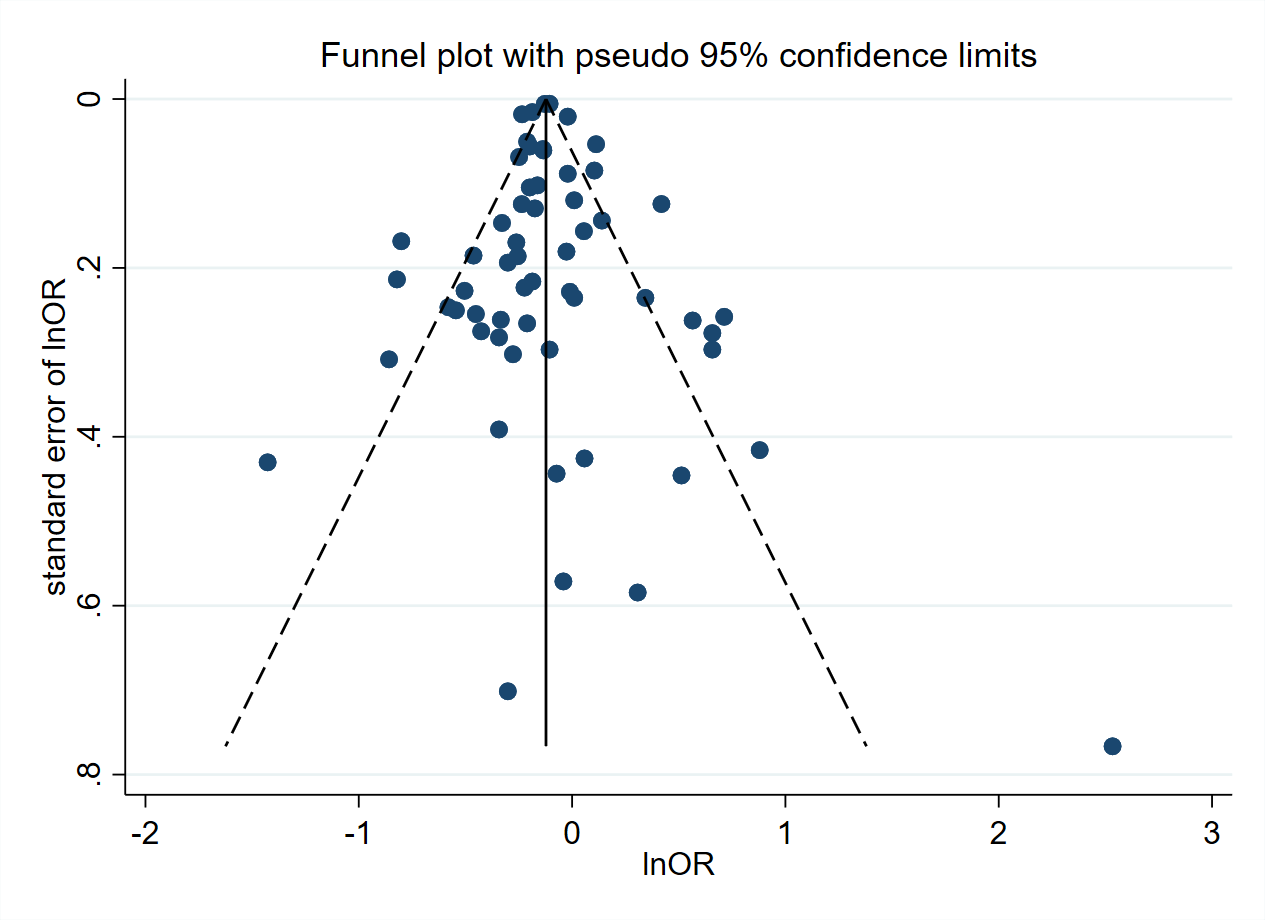


（C）Funnel plot of the impact of obesity (measured by BMI) and cognitive disorders.


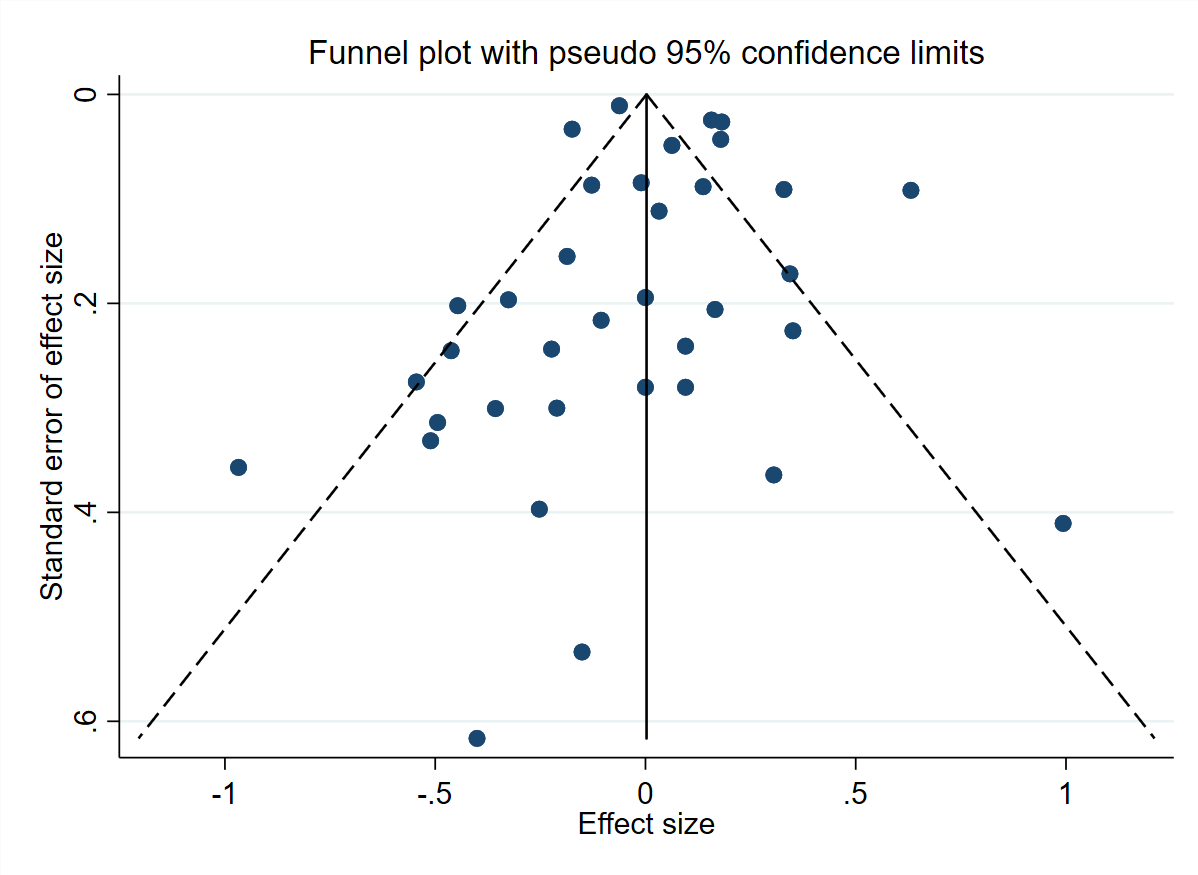


（D）Funnel plot of the impact of WC and cognitive disorders.


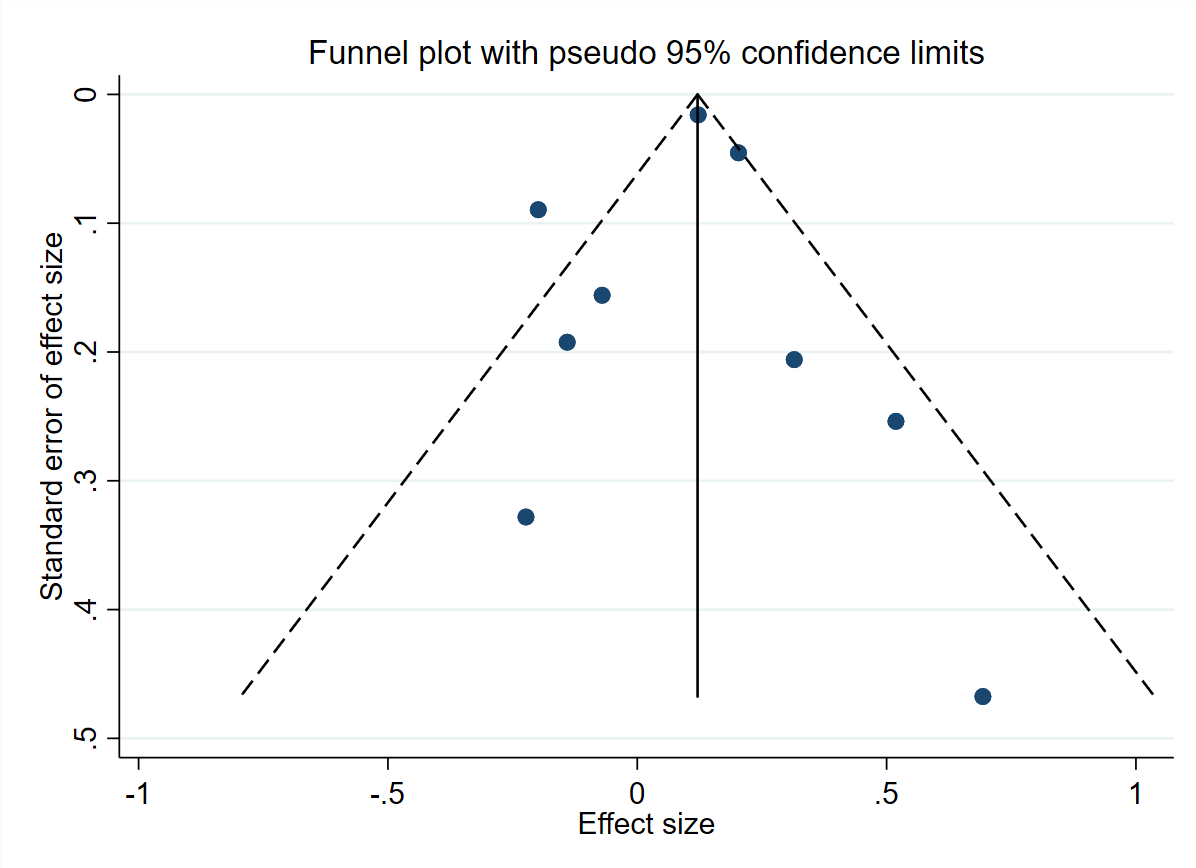


（E）Funnel plot of the impact of WHR and cognitive disorders.


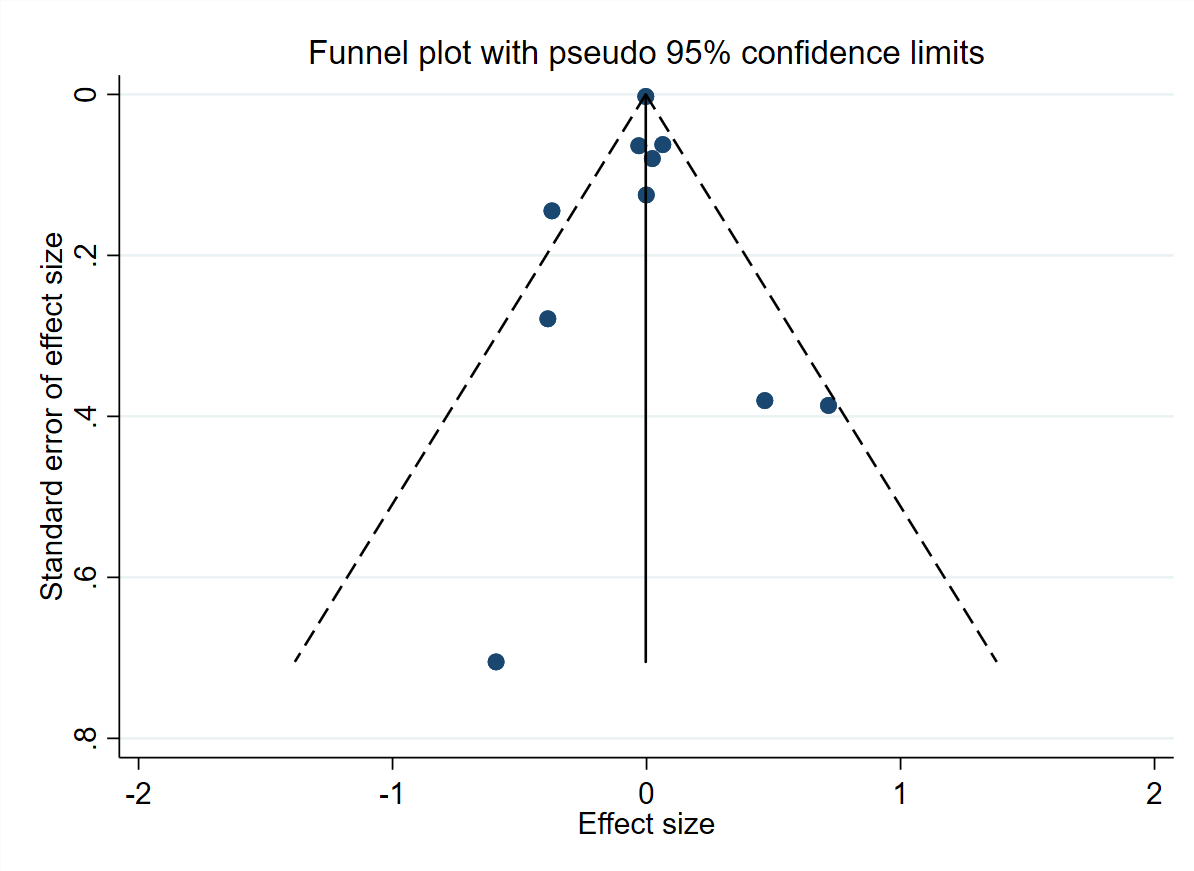


（F）Funnel plot of the impact of visceral obesity and cognitive disorders.


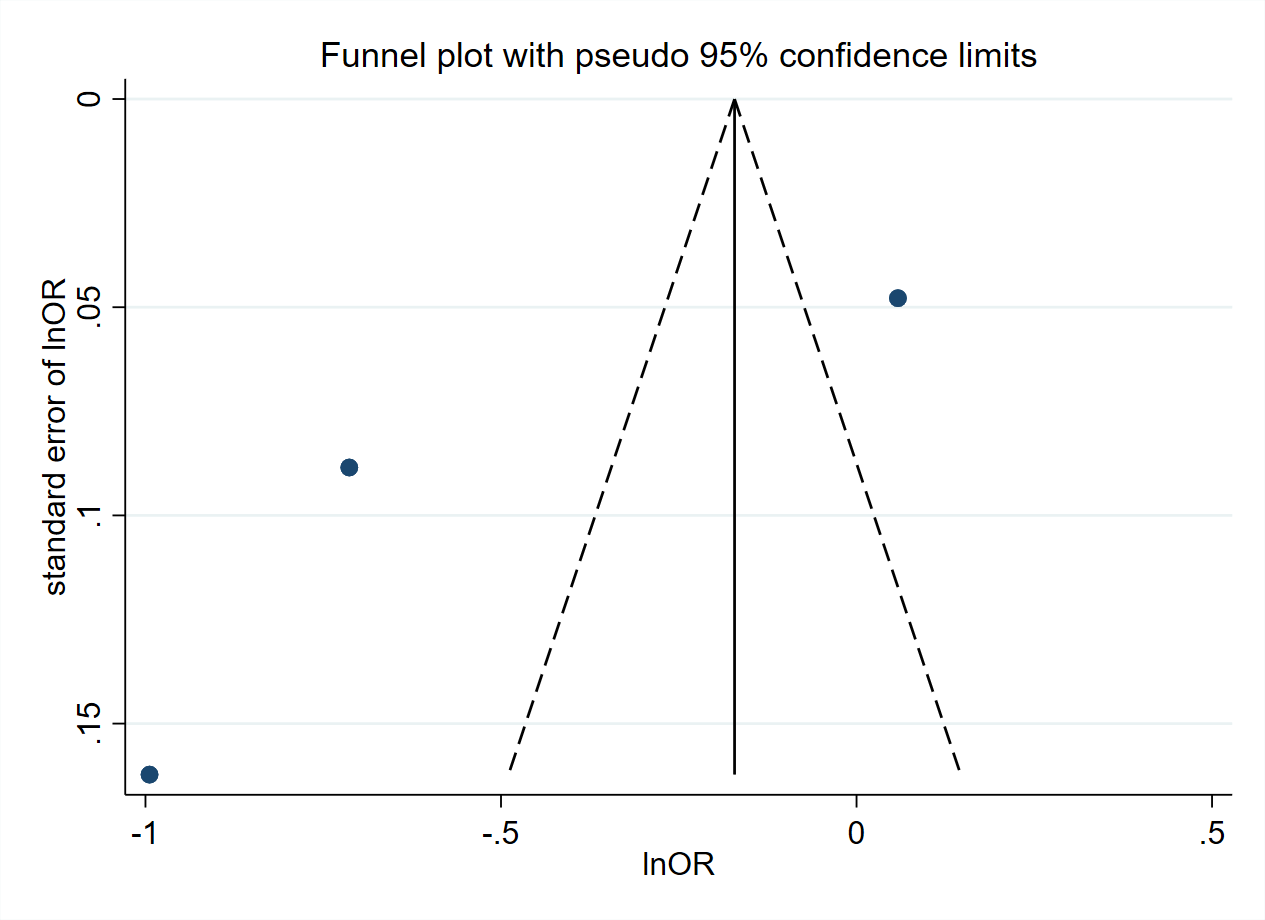


（G）Funnel plot of the relationship between body fat percentage and the impact of cognitive impairment.

*Note*: BMI, body mass index; WC, waist circumference; WHR, waist-to-hip ratio.

Supplementary Figure 3. Egger test for identifying publication bias

（A）The Egger test for the impact of underweight (measured by BMI) on cognitive impairment.

（B）The Egger test for the impact of overweight (measured by BMI) on cognitive impairment.

（C）The Egger test for the impact of obesity (measured by BMI) on cognitive impairment.

（D）Egger test for the influence of WC on cognitive impairment.

（E）Egger test for the influence of WHR on cognitive impairment.

（F）Egger test for the impact of visceral obesity on cognitive impairment.

（G）Egger test for the influence of body fat percentage on cognitive impairment.

*Note*: BMI, body mass index; WC, waist circumference; WHR, waist-to-hip ratio.

Supplementary Figure 4. Scatter plot of causal relationships between general obesity, central obesity, and cognitive impairment

(A) BMI and Cognitive performance  (B) BMI and Cognitive function


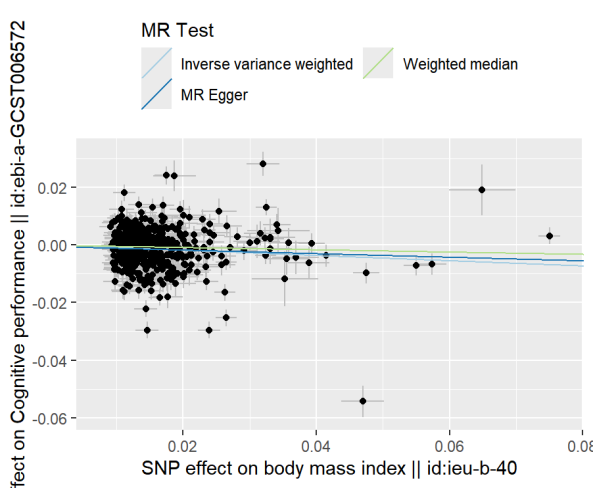

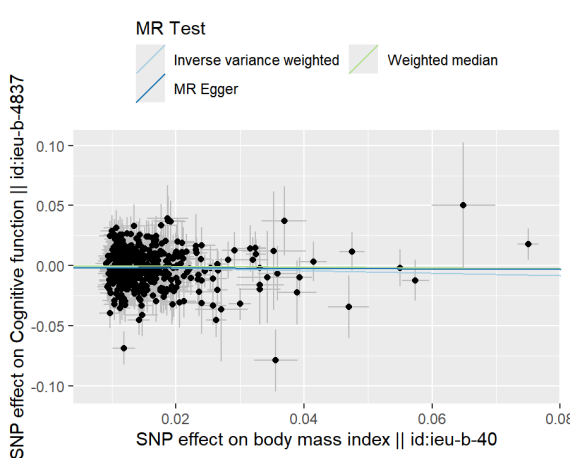


(C) BMI and AD (D) BMI and Dementia


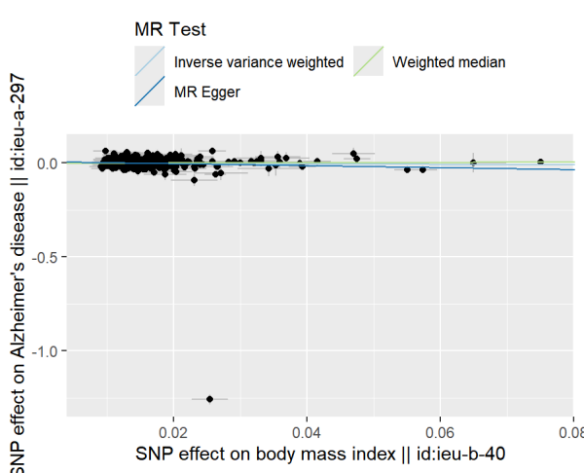

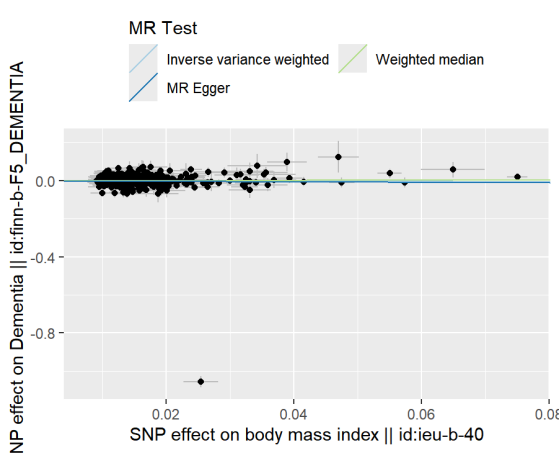


(E) WHR and Cognitive performance (F) WHR and Cognitive function


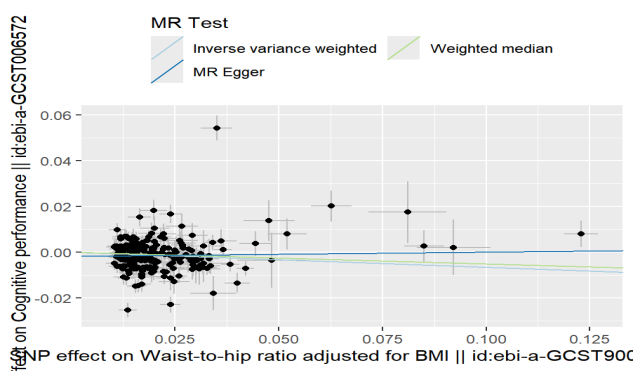

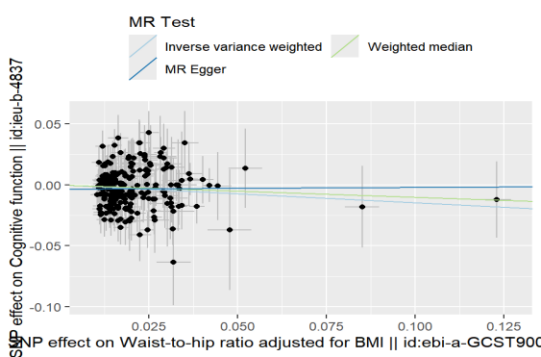


(G) WHR and AD (H) WHR and Dementia


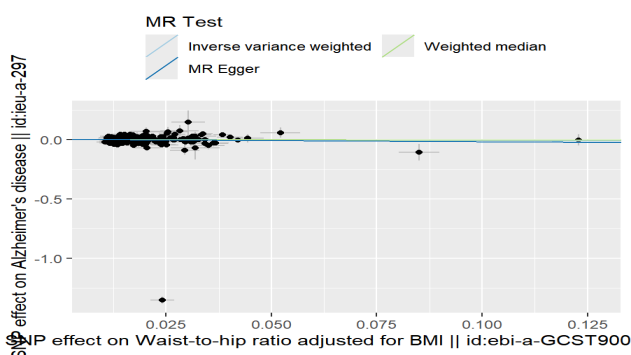

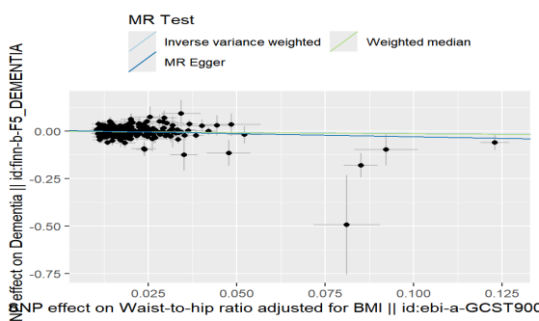


(I) WC and Cognitive performance (J) WC and Cognitive function


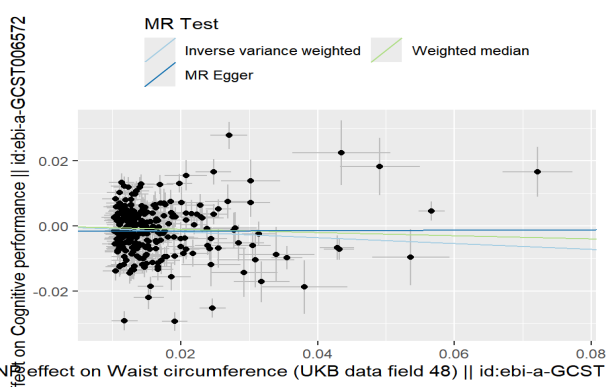

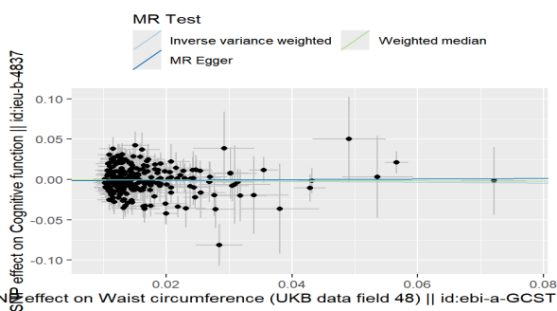


(G) WC and AD (H) WC and Dementia


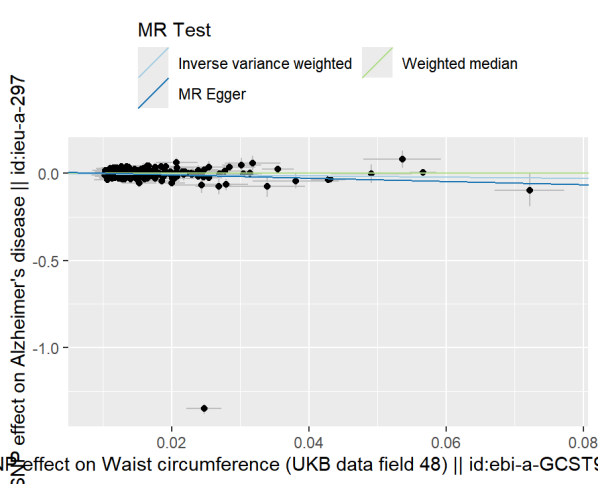

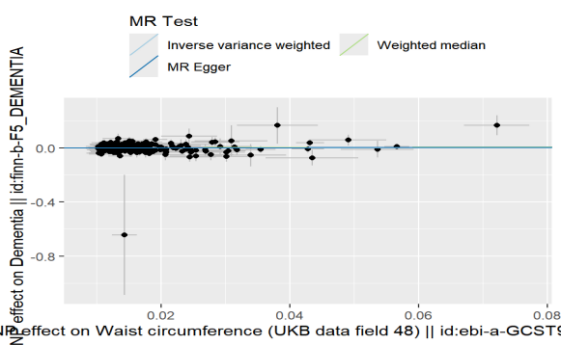


(I) VAT and Cognitive performance (J) VAT and Cognitive function


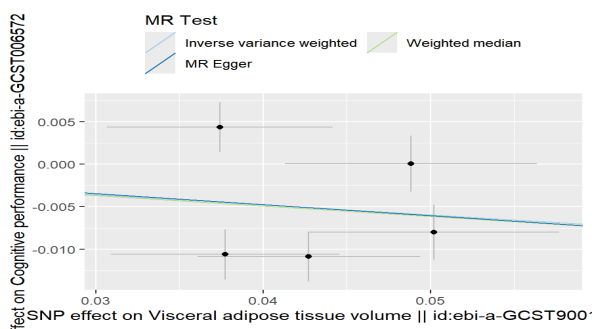

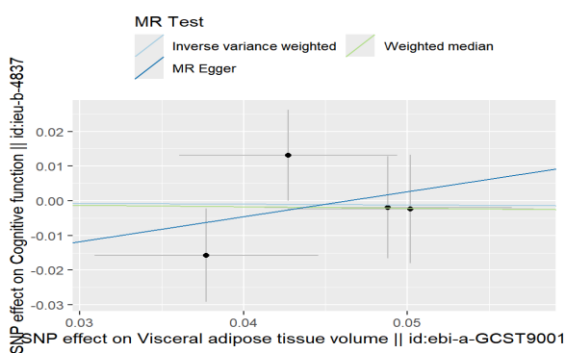


(K) VAT and AD (L) VAT and Dementia


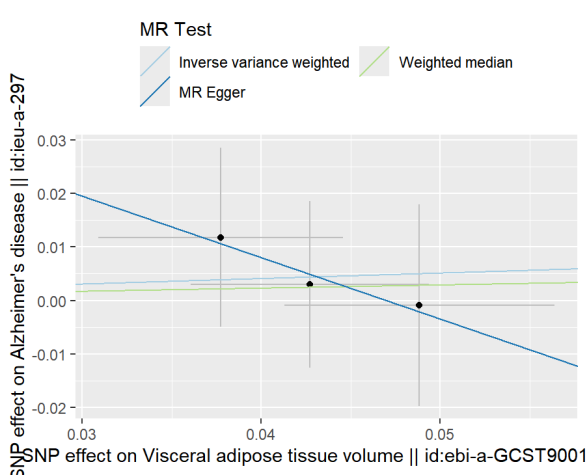

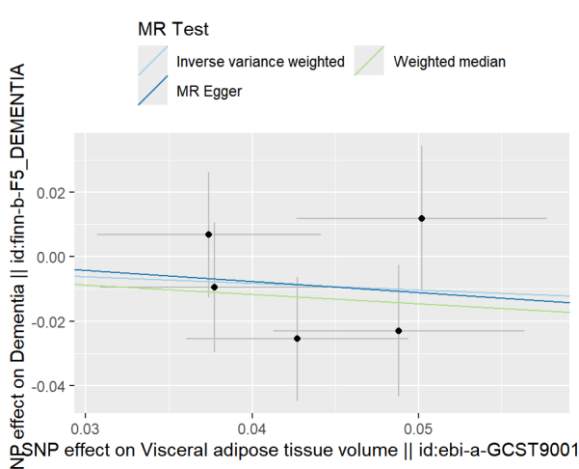


(M) BFP and Cognitive performance (N) BFP and Cognitive function


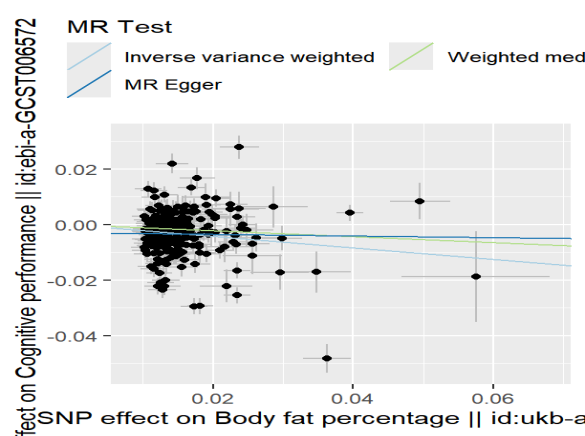

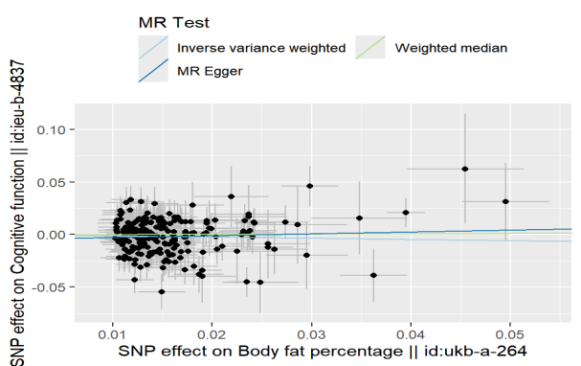


(O) BFP and AD (P) BFP and Dementia


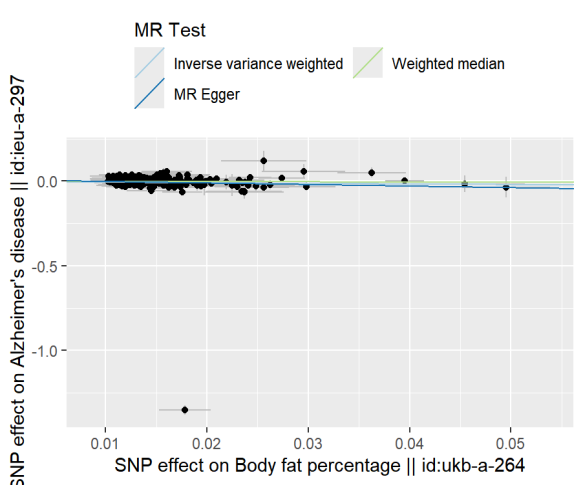

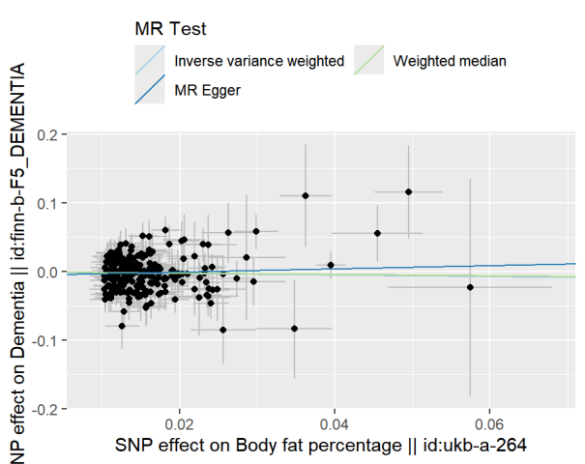


*Note*: BMI, body mass index; WC, waist circumference; WHR, waist-to-hip ratio; VAT, Visceral adipose tissue; BFP, Body fat percentage; AD, Alzheimer's disease.

Supplementary Figure 5. A funnel plot of the causal relationship between general obesity, central obesity, and visceral obesity and cognitive impairment.

(A) BMI and CI (B) BMI and Cognitive function


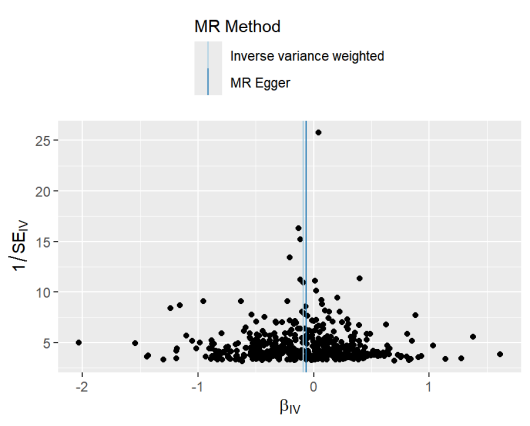

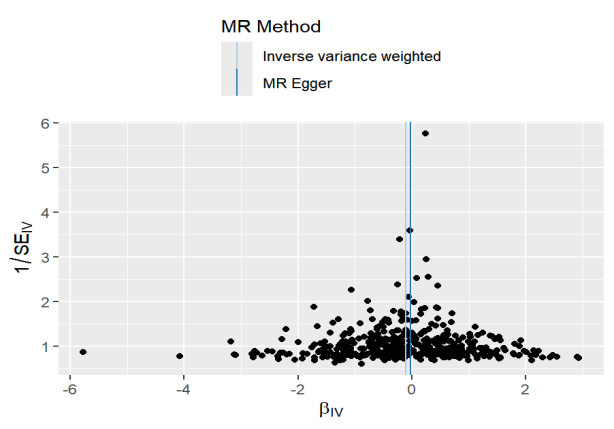


(C) BMI and AD (D) BMI and Dementia


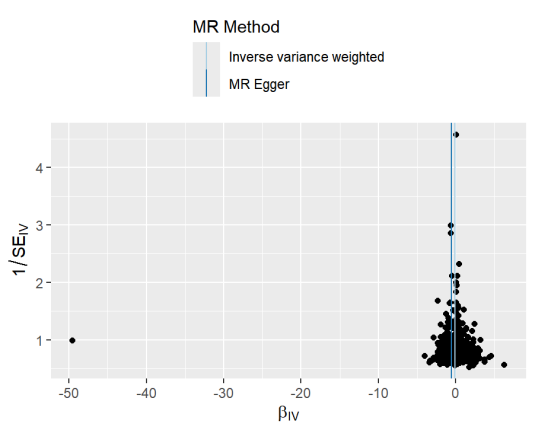

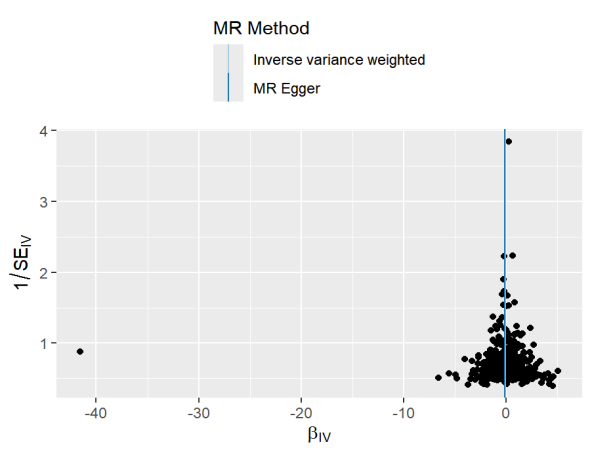


(E) WC and CI (F) WC and Cognitive function


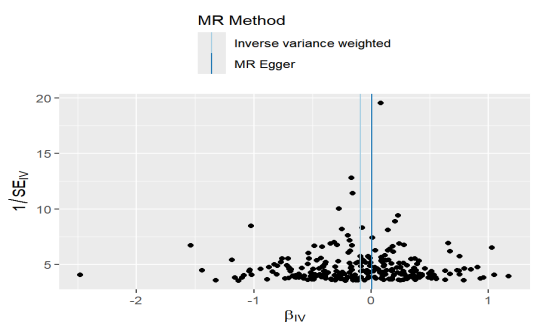

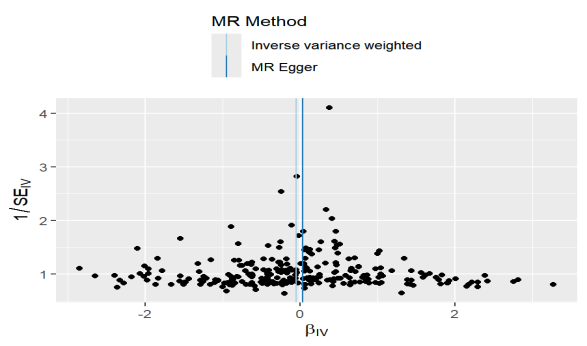


(G) WC and AD (H) WC and Dementia


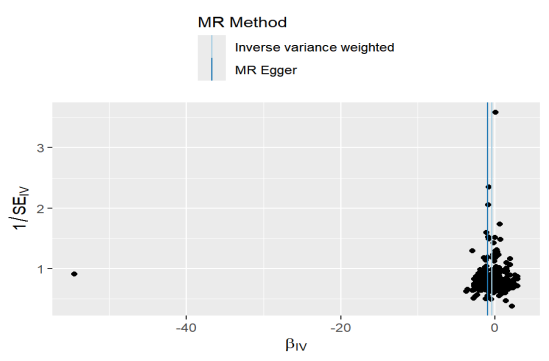

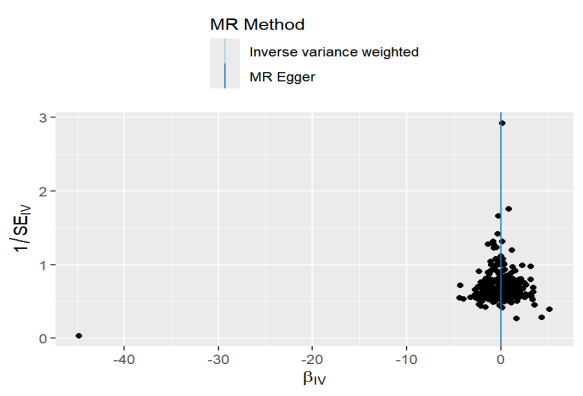


(I) WHR and Cognitive function (J) WHR and Cognitive function


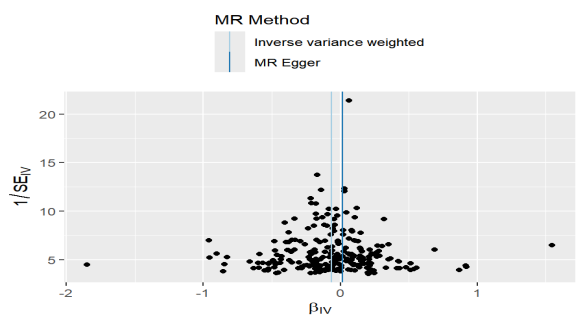

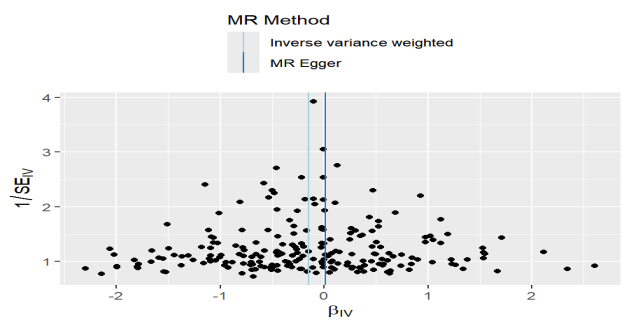


(K) WHR and AD (L) WHR and Dementia


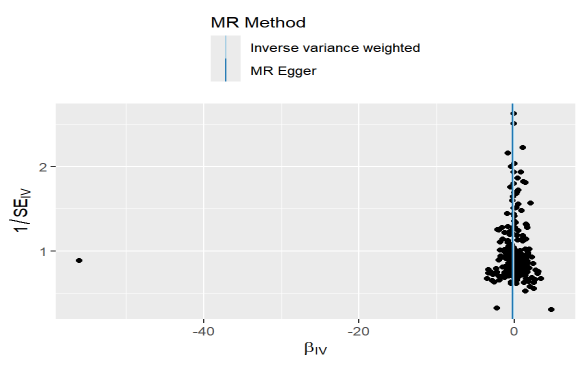

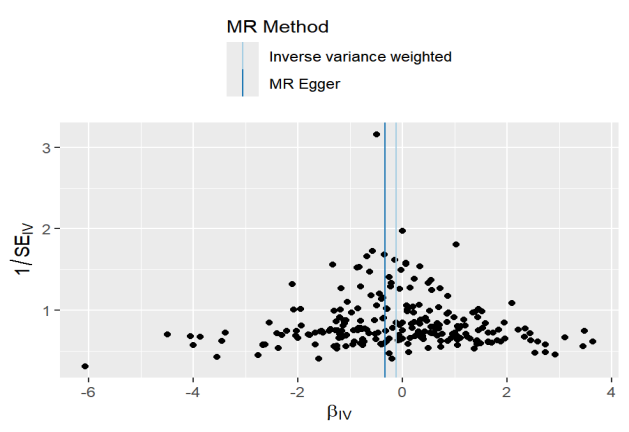


(M) VAT and Cognitive function (N) VAT and Cognitive function


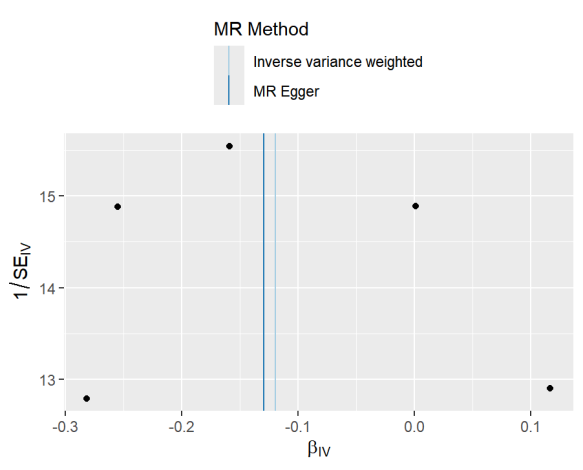

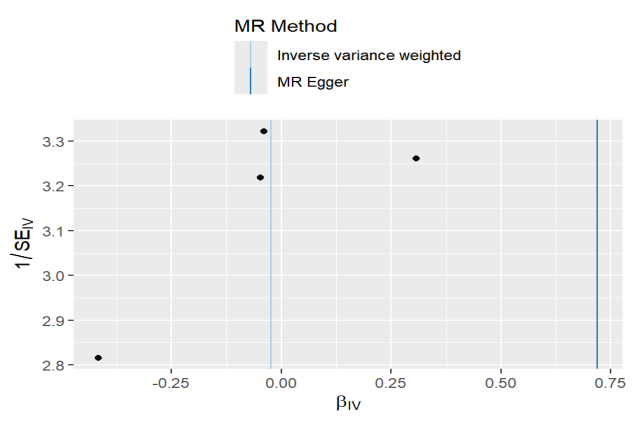


(O) VAT and AD (P) VAT and Dementia


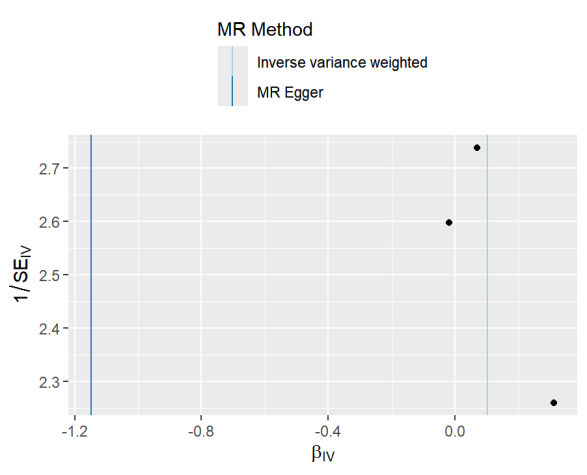

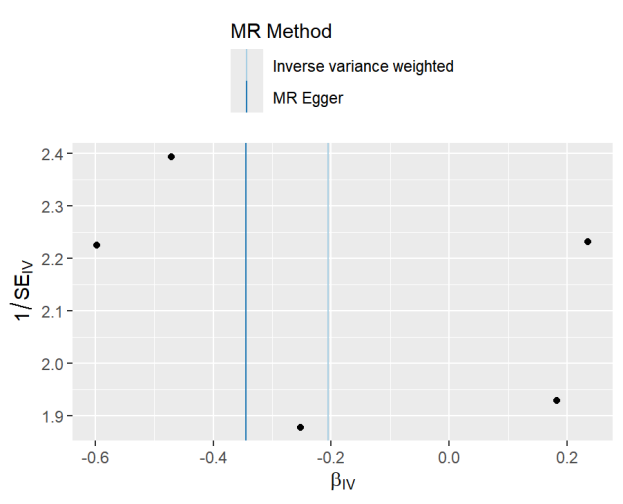


(Q) BFP and Cognitive function (R) BFP and Cognitive function


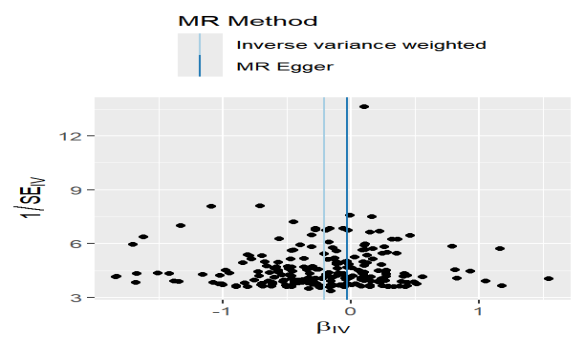

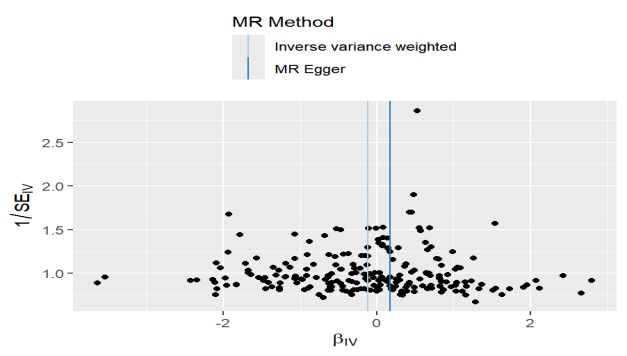


(S) BFP and AD (T) BFP and Dementia


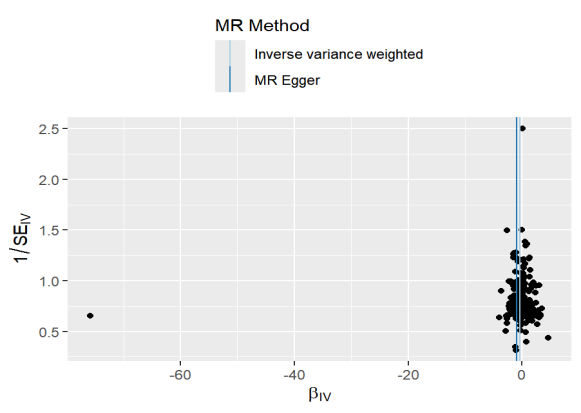

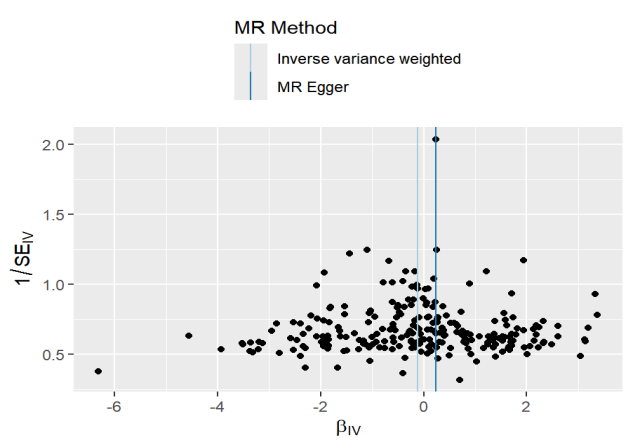


*Note*: BMI, body mass index; WC, waist circumference; WHR, waist-to-hip ratio; VAT, Visceral adipose tissue; BFP, Body fat percentage; AD, Alzheimer's disease.

Supplementary Figure 6. Leave-one-out sensitivity analysis of MR for general obesity, central obesity, and visceral obesity and cognitive impairment

1. BMI and Cognitive function (B) BMI and Cognitive performance


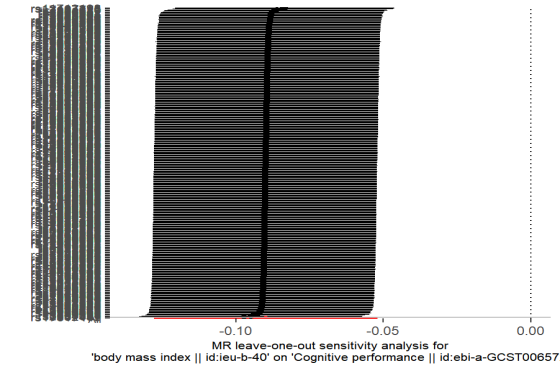

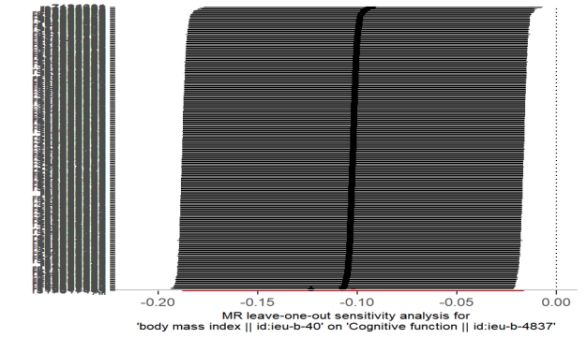


(C) BMI and AD (D) BMI and Dementia


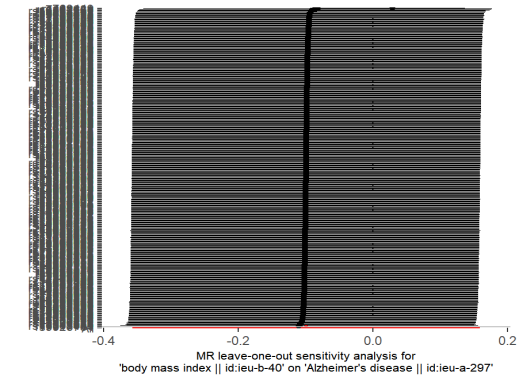

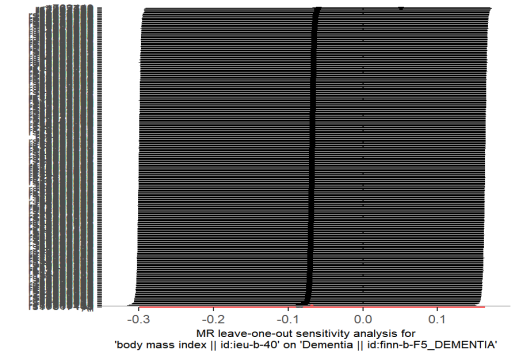


(E) WC and Cognitive function (F) WC and Cognitive performance


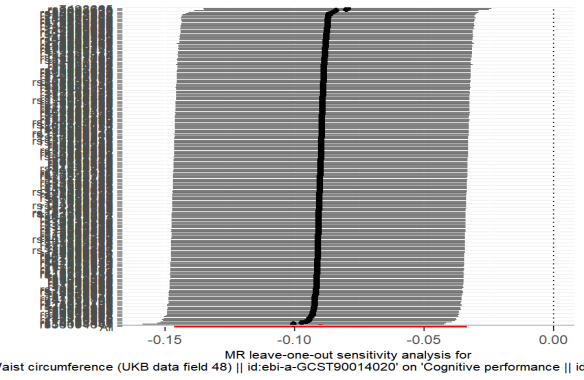

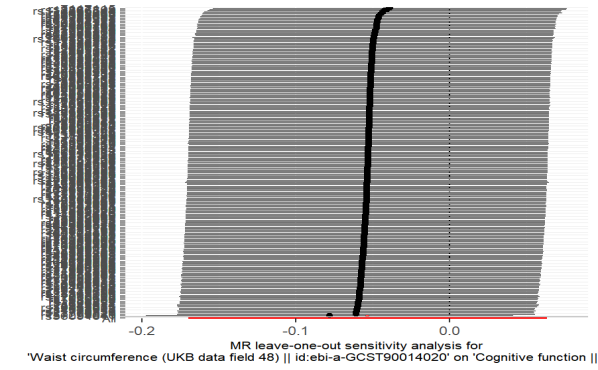


(G) WC and AD (H) WC and Dementia


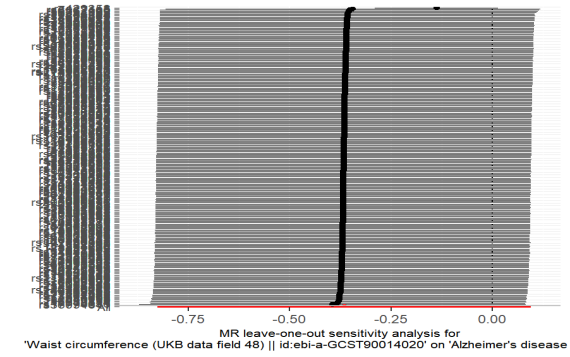

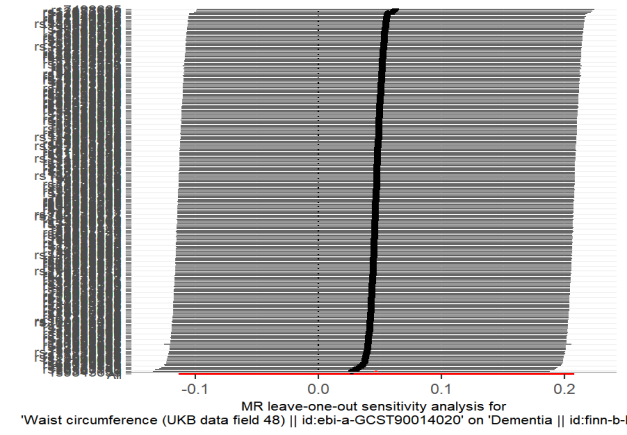


(I) WHR and Cognitive function (J) WHR and Cognitive performance


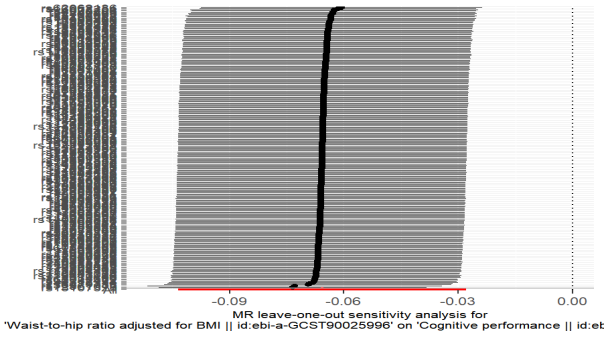

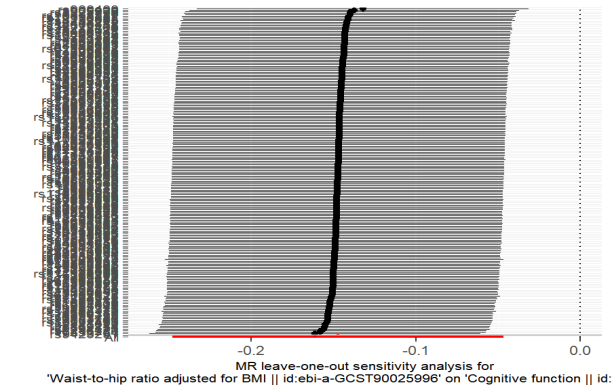


(K) WHR and AD (L) WHR and Dementia


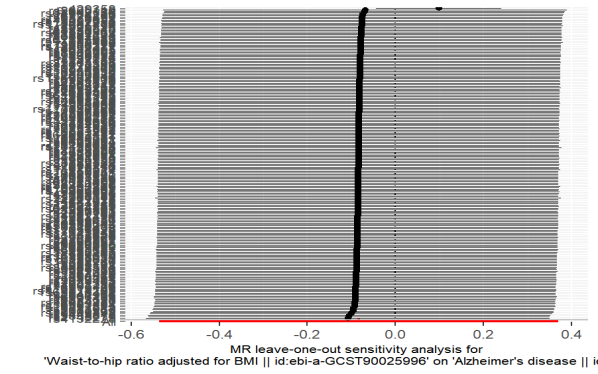

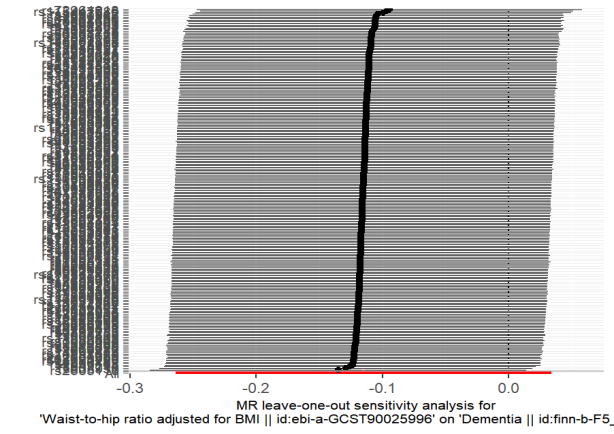


(M) VAT and Cognitive function (N) VAT and Cognitive performance


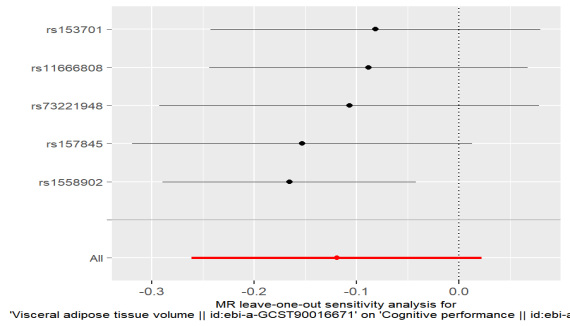

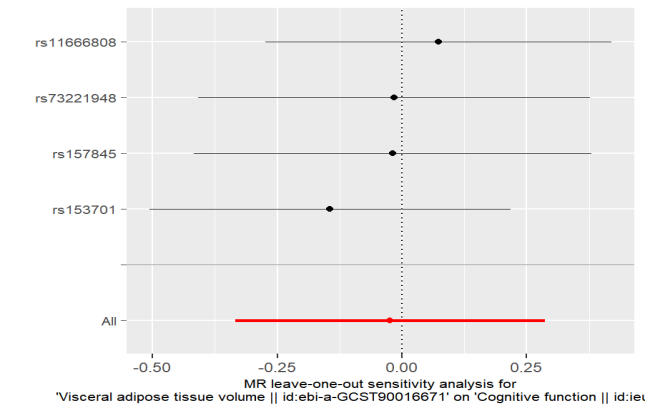


(O) VAT and AD (P) VAT and Dementia


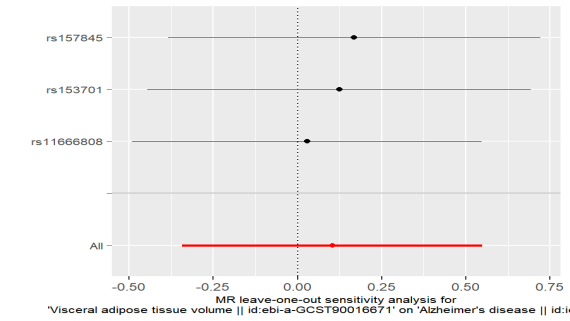

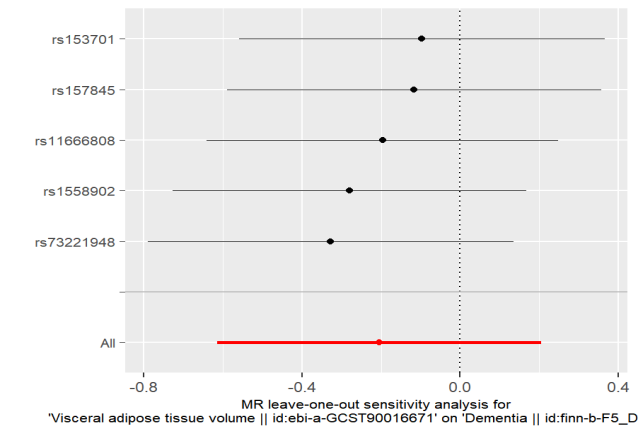


(Q) BFP and Cognitive function (R) BFP and Cognitive performance


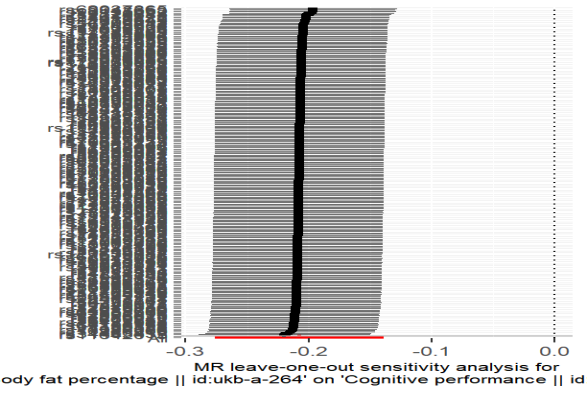

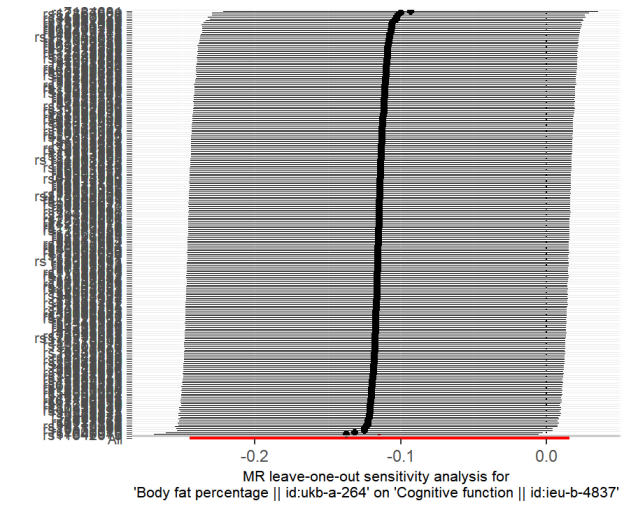


(S) BFP and AD (T) BFP and Dementia


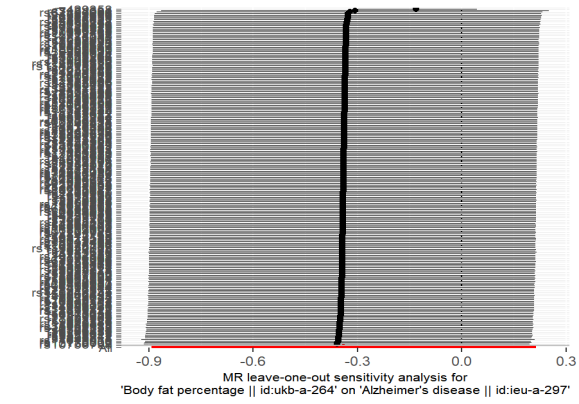

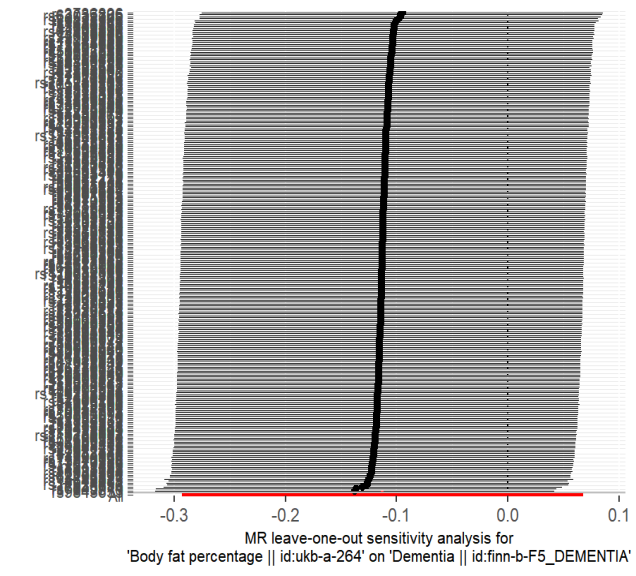


*Note*: BMI, body mass index; WC, waist circumference; WHR, waist-to-hip ratio; VAT, Visceral adipose tissue; BFP, Body fat percentage; AD, Alzheimer's disease.

Supplementary Figure 7. Detailed results of sensitivity analysis on MR
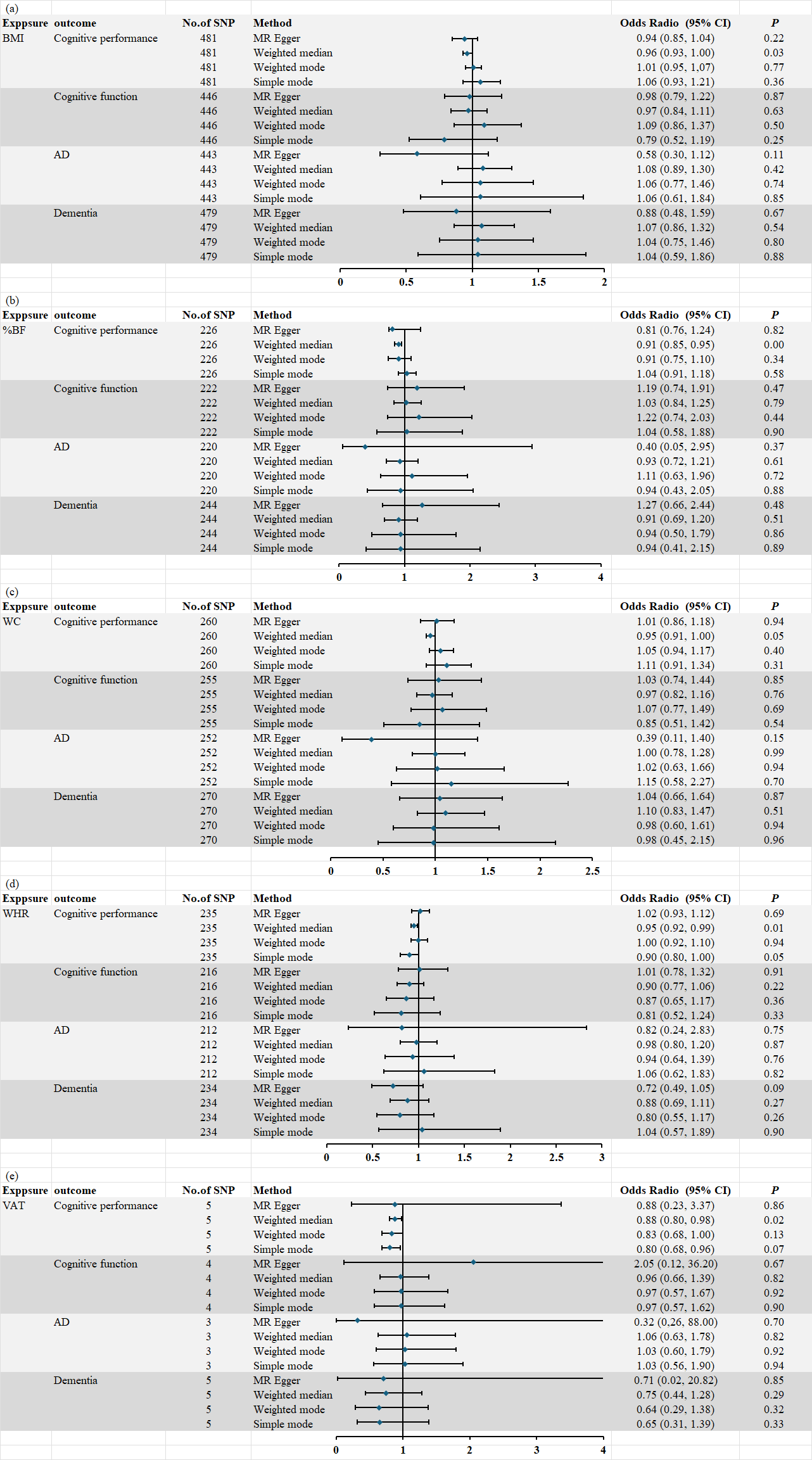


*Note*: BMI, body mass index; WC, waist circumference; WHR, waist-to-hip ratio; %BF, Percentage of body fat; VAT, visceral adiposity tissue; AD, Alzheimer's disease.
